# Supplementary material for: Structure and Anti-Inflammatory Activity Relationship of Ergostanes and Lanostanes in Antrodia cinnamomea
Source: Foods. 2022 Jun 22;11(13):1831. doi: 10.3390/foods11131831 (PMC9266224; doi:10.3390/foods11131831)
Supplement: Supplementary file 1 [file foods-11-01831-s001.zip › foods-1760315-Supplementary material.pdf]

Support Information

Structural Characterizations and Anti-inflammatory Activity of Ergostanes and Lanostanes in *Antrodia cinnamomea*

Xin Yang*,[†] Xiang Wang,[†] Jiachen Lin,[†] Sophie Lim,[†] Yujia Cao,[†] Siyu Chen,[†] Pingkang Xu,[†] Chunyuhang Xu,[†] Hongling Zheng,[†] Kuo-Chang Fu[‡], Chian-Liang Kuo[‡], Dejian Huang[*,†,§]

Affiliations:

[†]Department of Food Science and Technology, National University of Singapore, 2 Science Drive 2, Singapore 117542, Republic of Singapore

[‡]AgriGADA Biotech Pte Ltd, 8 Eu Tong Sen Street #17-82, The Central, Singapore 059818

[§]National University of Singapore (Suzhou) Research Institute, 377 Linquan Street,

Suzhou, Jiangsu 215123, China

*.. Corresponding authors: Xin Yang (yangxin@nus.edu.sg) and Dejian Huang (fsthdj@nus.edu.sg)

1. Materials and methods

Spectroscopic data of isolated compounds

Compound 1, 2,3,4-trimethoxy-6-methylphenol.

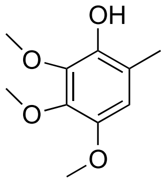

^{1}H NMR (500 MHz, DMSO-d_6) δ 8.43 (s, 1H), 6.50 (s, 1H), 3.73 (s, 3H), 3.71 (s, 3H), 3.66 (s, 3H), 2.10 (s, 3H). ^{13}C NMR (126 MHz, DMSO-d_6) δ 145.64, 144.82, 141.87, 138.53, 119.79, 109.05, 101.22, 60.61, 56.53, 15.78. HRMS (ESI-TOF) calcd for $C_{10}H_{14}O_4$ = 197.0819, found at 197.8081.

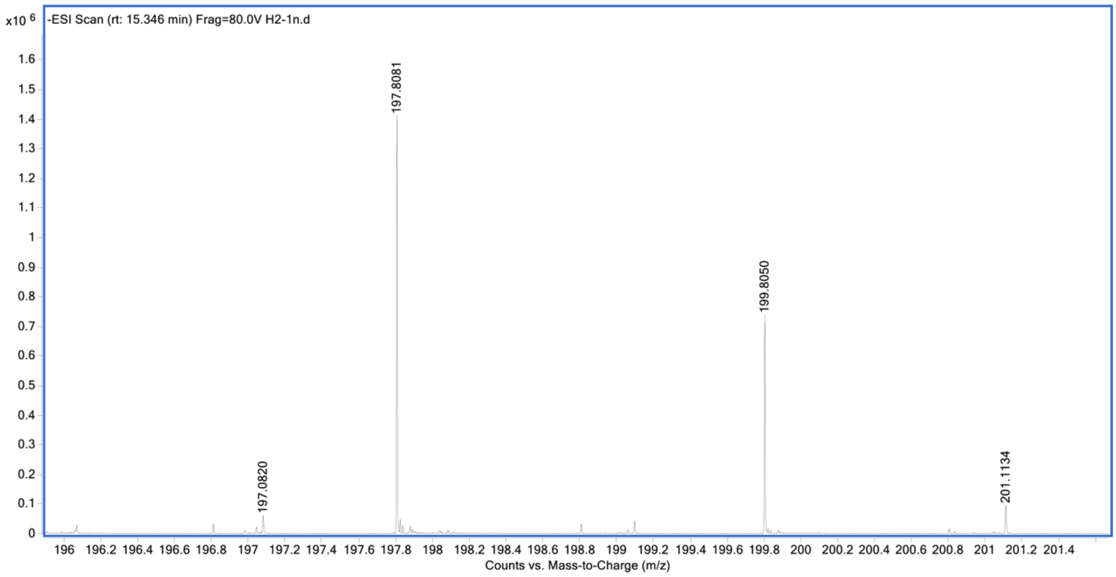

Compound 4, (25S)-Antcin K, (2*S*,6*R*)-2-methyl-3-methylene-6-((3*R*,4*R*,5*R*,7*S*,10*S*,13*R*,14*R*,17*R*)-3,4,7-trihydroxy-4,10,13-trimethyl-11-oxo-2,3,4,5,6,7,10,11,12,13,14,15,16,17-tetradecahydro-1*H*-cyclopenta[*a*]phenanthren-17-yl)heptanoic acid.

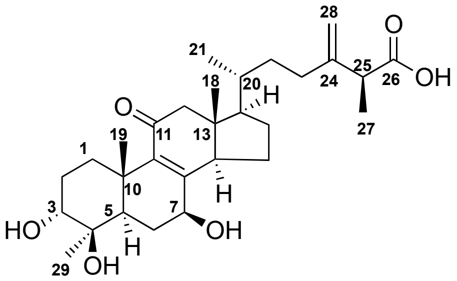

^{1}H NMR (400 MHz, DMSO-d_6) δ 4.85 (s, 1H), 4.82 (s, 1H), 4.62 (s, 1H), 4.38 (s, 1H), 4.15 (s, 1H), 3.91 (s, 1H), 3.22 (s, 1H), 3.03 (q, *J* = 7.0 Hz, 1H), 2.63 (dd, *J* = 12.4, 6.3 Hz, 1H), 2.53 (d, *J* = 13.8 Hz, 1H), 2.39 (d, *J* = 13.8 Hz, 1H), 2.26 (dt, *J* = 13.0, 3.8 Hz, 1H), 2.18 – 1.71 (m, 7H), 1.56 (m, 2H), 1.45 – 0.95 (m, 17H), 0.87 (d, *J* = 5.4 Hz, 3H), 0.65 (s, 3H). ^{13}C NMR (126 MHz, DMSO-d_6) δ 201.20, 175.72, 154.00, 143.08, 110.70, 110.57, 73.27, 73.01, 69.94, 58.28, 54.01, 53.24, 47.63, 45.32, 42.52, 37.73, 35.71, 33.94, 31.29, 29.11, 28.83, 27.90, 27.43, 25.64, 24.96, 20.47, 18.71, 16.69, 12.43. HRMS (ESI-TOF) calcd for $C_{29}H_{44}O_6$ = 487.3065, found at 487.3058.

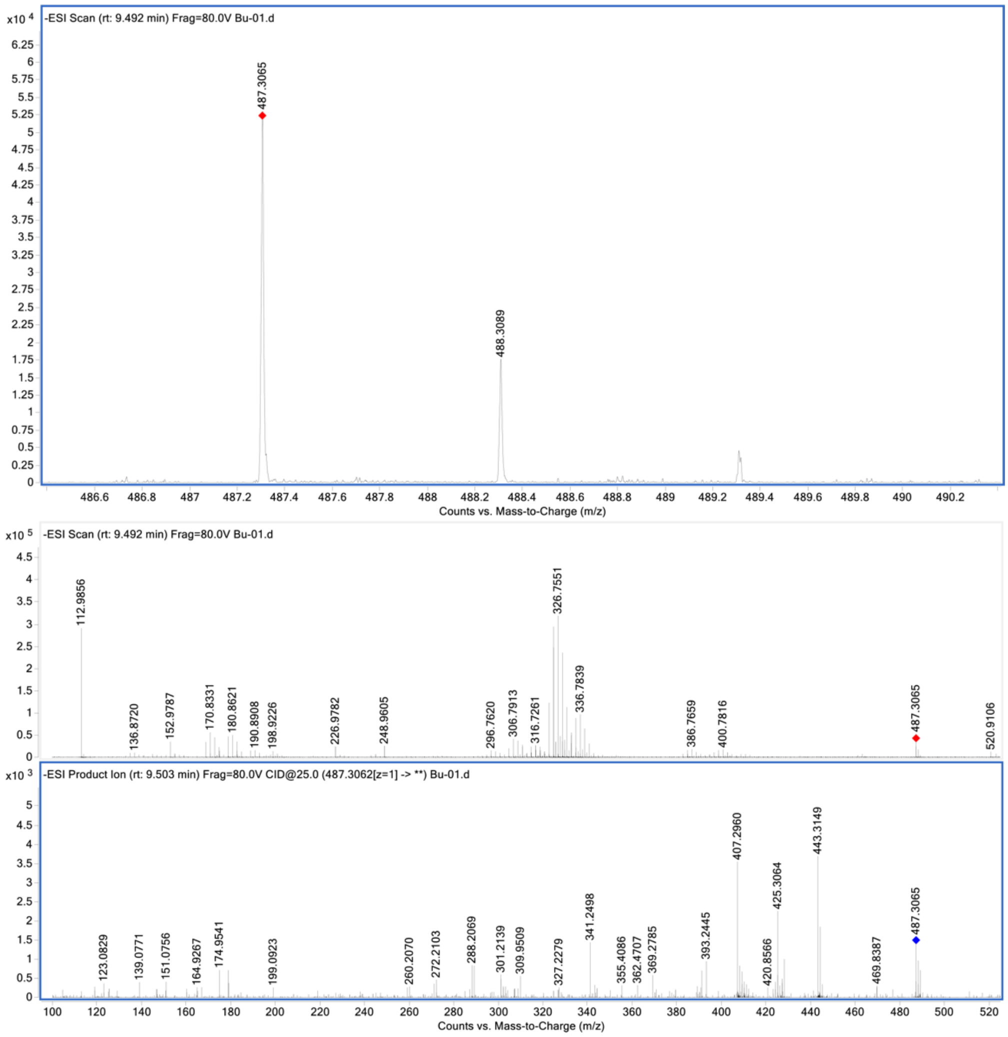

Compound 5, (25R)-Antcin K, (2*R*,6*R*)-2-methyl-3-methylene-6-((3*R*,4*R*,5*R*,7*S*,10*S*,13*R*,14*R*,17*R*)-3,4,7-trihydroxy-4,10,13-trimethyl-11-oxo-2,3,4,5,6,7,10,11,12,13,14,15,16,17-tetradecahydro-1*H*-cyclopenta[*a*]phenanthren-17-yl)heptanoic acid.

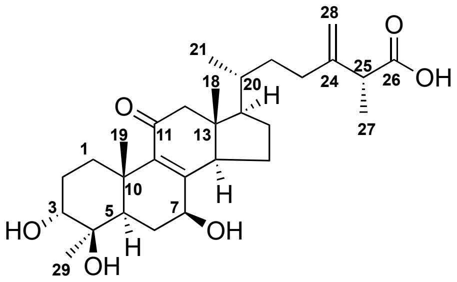

^{1}H NMR (500 MHz, DMSO-d_6) δ 4.84 (s, 1H), 4.81 (s, 1H), 4.62 (s, 1H), 4.37 (s, 1H), 4.16 (t, *J* = 8.7 Hz, 1H), 3.91 (s, 1H), 3.23 (s, 1H), 3.02 (q, *J* = 7.0 Hz, 1H), 2.63 (dd, *J* = 12.5, 6.4 Hz, 1H), 2.53 (d, *J* = 13.45Hz, 1H), 2.38 (d, *J* = 13.5 Hz, 1H), 2.26 (dd, *J* = 13.0, 3.6 Hz, 1H), 2.17 – 1.73 (m, 8H), 1.57 (m, 3H), 1.45 – 1.02 (m, 16H), 0.87 (d, *J* = 5.3 Hz, 3H), 0.65 (s, 3H). ^{13}C NMR (126 MHz, DMSO) δ 201.19, 175.74, 153.96, 149.51, 143.10, 110.55, 73.28, 73.03, 69.96, 58.29, 54.05, 53.25, 47.63, 45.56, 42.52, 37.73, 35.73, 34.05, 31.18, 29.11, 28.82, 27.86, 27.42, 25.64, 24.96, 20.46, 18.75, 16.88, 12.42. HRMS (ESI-TOF) calcd for $C_{29}H_{44}O_6$ = 487.3065, found at 487.3063.

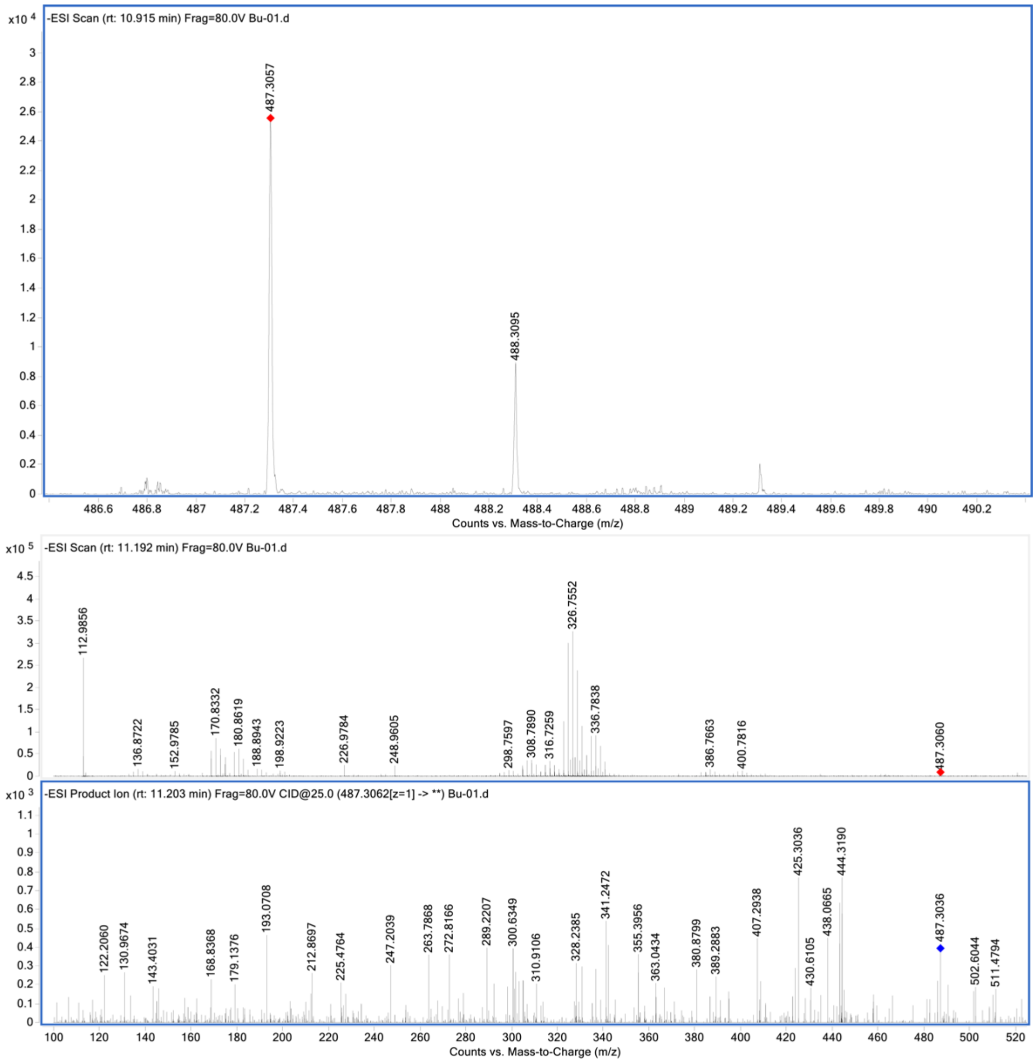

Compound 8, Antcin G, (2*R*,6*R*)-6-((4*S*,7*R*,10*S*,13*R*,14*R*,17*R*)-7-acetoxy-4,10,13-trimethyl-3,11-dioxo-2,3,4,5,6,7,10,11,12,13,14,15,16,17-tetradecahydro-1*H*-cyclopenta[*a*]phenanthren-17-yl)-2-methyl-3-methyleneheptanoic acid

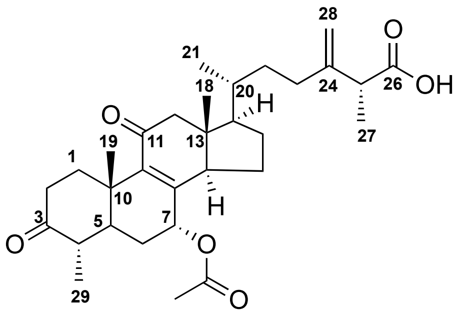

^{1}H NMR (500 MHz, DMSO-d_6) δ 5.28 (d, *J* = 3.3 Hz, 1H), 4.84 (s, 1H), 4.80 (s, 1H), 3.01 (q, *J* = 6.9 Hz, 1H), 2.98 – 2.90 (m, 1H), 2.72 (dd, *J* = 12.3, 7.5 Hz, 1H), 2.63 (d, *J* = 14.6 Hz, 1H), 2.48 (d, *J* = 2.5 Hz, 1H), 2.38 (dd, *J* = 12.6, 6.4 Hz, 1H), 2.22 – 2.14 (m, 1H), 2.08 (dq, *J* = 10.6, 5.6 Hz, 2H), 1.97 – 1.82 (m, 3H), 1.68 (dd, *J* = 6.6, 2.8 Hz, 2H), 1.59 – 1.44 (m, 6H), 1.25 (s, 6H), 1.18 – 1.06 (m, 7H), 0.88 (t, *J* = 8.4 Hz, 10H), 0.65 (s, 3H). ^{13}C NMR (126 MHz, DMSO-d_6) δ 200.19, 175.63, 170.04, 149.28, 142.28, 110.60, 68.29, 57.36, 54.44, 50.64, 47.45, 45.56, 45.39, 45.32, 43.14, 37.40, 37.09, 35.54, 34.76, 33.80, 31.22, 27.75, 27.39, 22.70, 21.96, 21.28, 18.52, 16.83, 16.65, 16.36, 16.29, 12.07, 12.02, 11.89. HRMS (ESI-TOF) calcd for $C_{31}H_{44}O_6$ = 511.3065, found at 511.3425.

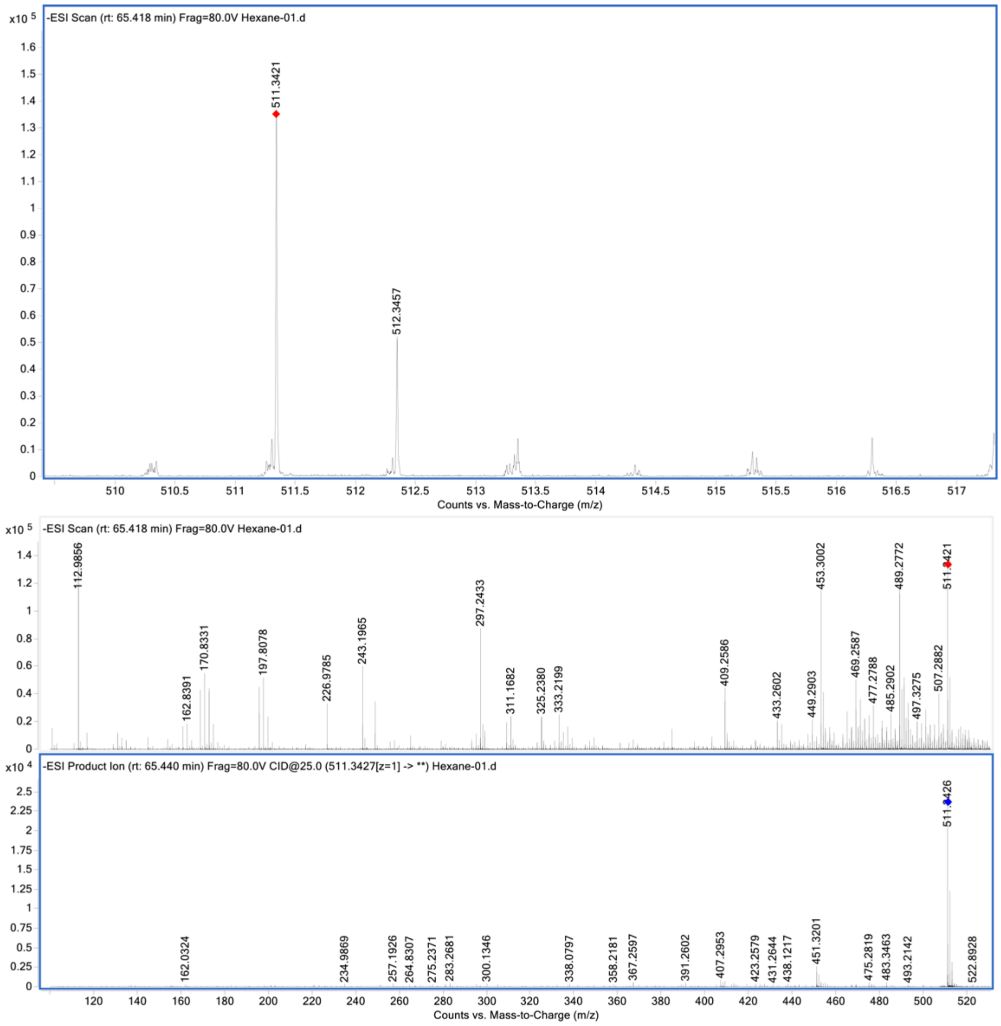

Compound 10, (25R)-Antcin A, (2*R*)-2-methyl-3-methylene-6-((4*S*,5*S*,10*S*,13*R*,14*R*,17*R*)-4,10,13-trimethyl-3,11-dioxo-2,3,4,5,6,7,10,11,12,13,14,15,16,17-tetradecahydro-1*H*-cyclopenta[*a*]phenanthren-17-yl)heptanoic acid.

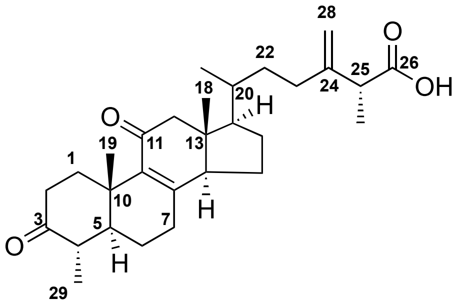

^{1}H NMR (500 MHz, DMSO-d_6) δ 4.85 (d, *J* = 3.7 Hz, 1H), 4.82 (d, *J* = 5.0 Hz, 1H), 3.07 – 2.98 (m, 2H), 2.66 (dd, *J* = 12.6, 7.2 Hz, 1H), 2.60 – 2.28 (m, 9H), 2.15 (dddd, *J* = 35.4, 17.6, 14.6, 6.9 Hz, 5H), 1.99 – 1.83 (m, 4H), 1.80 – 1.65 (m, 4H), 1.58 – 1.11 (m, 21H), 0.93 (d, *J* = 6.6 Hz, 5H), 0.87 (d, *J* = 6.4 Hz, 5H), 0.65 (s, 3H). ^{13}C NMR (126 MHz, DMSO-d_6) δ 212.31, 199.44, 175.68, 157.76, 149.30, 138.12, 110.71, 110.59, 57.57, 54.69, 52.62, 50.33, 47.13, 45.31, 44.01, 37.69, 36.59, 35.61, 35.58, 35.08, 33.92, 33.82, 31.29, 31.14, 30.05, 27.50, 23.54, 20.88, 18.60, 18.54, 17.71, 16.85, 16.68, 12.12. HRMS (ESI-TOF) calcd for $C_{29}H_{42}O_4$ = 453.3010, found at 453.3007.

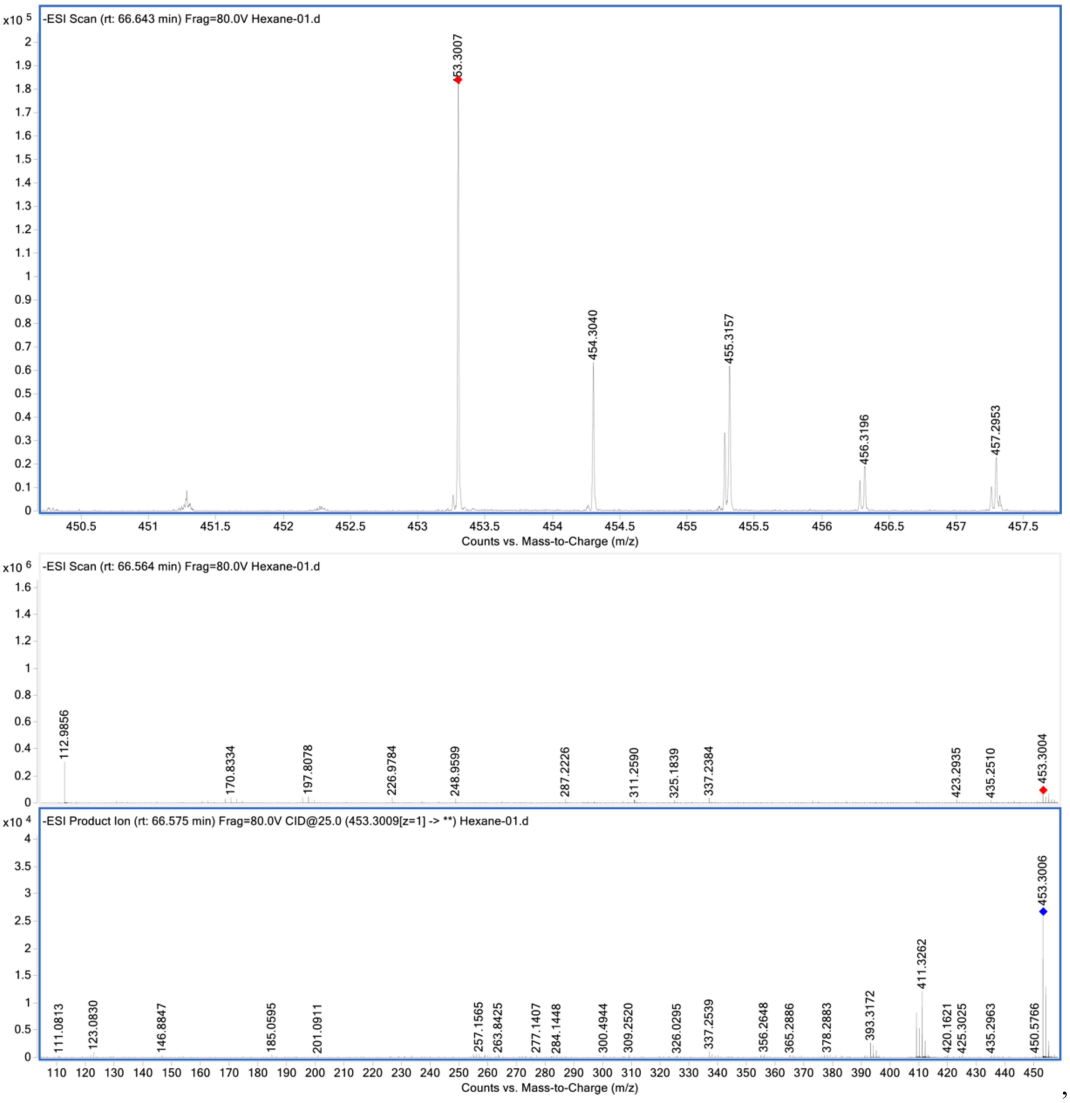

,

Compound 11, Versisponic acid D. 2-((3*S*,5*R*,10*S*,13*R*,14*R*,17*R*)-15-acetoxy-3-hydroxy-4,4,10,13,14-pentamethyl-2,3,4,5,6,7,10,11,12,13,14,15,16,17-tetradecahydro-1*H*-cyclopenta[*a*]phenanthren-17-yl)-6-methyl-5-methyleneheptanoic acid.

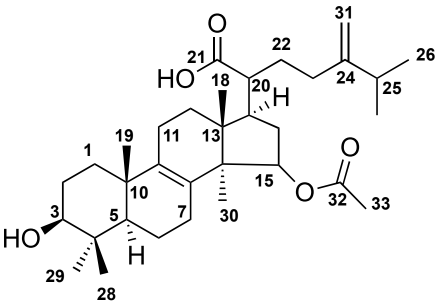

^{1}H NMR (500 MHz, DMSO-d_6) δ 5.58 – 5.30 (m, 1H), 4.93 (td, *J* = 9.9, 5.5 Hz, 1H), 4.72 (s, 1H), 4.64 (s, 1H), 4.34 (d, *J* = 36.0 Hz, 1H), 3.00 (dd, *J* = 11.0, 5.4 Hz, 1H), 2.29 – 1.85 (m, 16H), 1.82 (s, 2H), 1.74 – 1.59 (m, 5H), 1.49 (d, *J* = 7.3 Hz, 7H), 1.25 (d, *J* = 6.6 Hz, 4H), 1.02 – 0.93 (m, 10H), 0.91 (d, *J* = 6.7 Hz, 6H), 0.77 (s, 3H), 0.70 (s, 2H), 0.65 (s, 1H). ^{13}C NMR (126 MHz, DMSO-d_6) δ 177.34, 170.70, 155.21, 135.80, 132.40, 107.24, 77.20, 75.26, 50.60, 50.26, 47.67, 45.74, 44.52, 38.98, 37.10, 35.57, 35.36, 33.68, 31.89, 31.08, 28.58, 27.96, 26.26, 22.06, 21.99, 21.51, 21.46, 20.53, 19.32, 18.60, 18.27, 16.41, 16.27. HRMS (ESI-TOF) calcd for $C_{32}H_{50}O_5$ = 527.3742, found at 527.3737

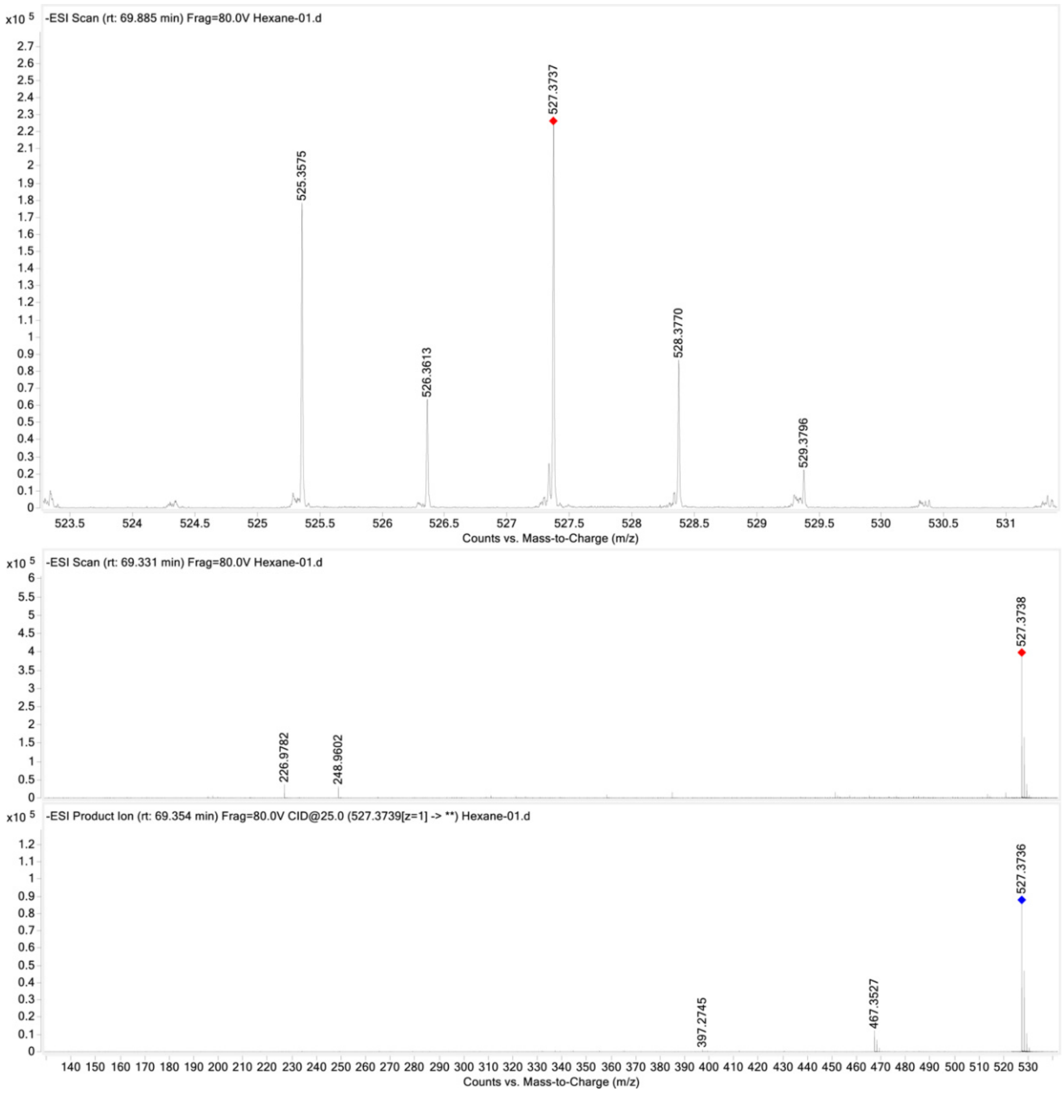

Compound 14, Eburicoic acid. (2*R*)-2-((5*R*,10*S*,13*R*,14*R*,17*R*)-3-hydroxy-4,4,10,13,14-pentamethyl-2,3,4,5,6,7,10,11,12,13,14,15,16,17-tetradecahydro-1*H*-cyclopenta[*a*]phenanthren-17-yl)-6-methyl-5-methyleneheptanoic acid.

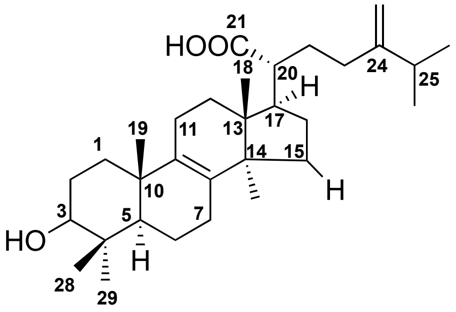

^{1}H NMR (400 MHz, DMSO-d_6) δ 6.43 (d, *J* = 8.5 Hz, 1H), 6.22 (d, *J* = 8.4 Hz, 1H), 5.19 (m, 1H), 4.72 (s, 1H), 4.64 (s, 1H), 4.35 (s, 1H), 3.00 (t, *J* = 8.0 Hz, 1H), 2.33 – 1.80 (m, 10H), 1.77 – 1.33 (m, 12H), 0.88 (ddt, *J* = 44.8, 29.3, 10.1 Hz, 16H), 0.70 (d, *J* = 4.4 Hz, 3H). ^{13}C NMR (126 MHz, DMSO-d_6) δ 177.51, 155.22, 136.04, 134.74, 133.82, 132.01, 130.52, 107.24, 77.25, 65.10, 51.69, 51.33, 50.50, 49.44, 48.04, 47.02, 44.27, 42.49, 39.20, 38.99, 37.30, 37.03, 35.68, 34.93, 34.13, 33.68, 32.93, 31.99, 31.10, 30.47, 30.35, 29.51, 29.19, 28.83, 28.55, 27.99, 26.95, 26.42, 24.49, 23.28, 22.56, 22.06, 21.97, 21.15, 20.76, 20.21, 19.90, 19.41, 18.33, 17.76, 16.25, 16.06, 14.39, 13.00. HRMS (ESI-TOF) calcd for $C_{29}H_{41}O_5$ = 469.2959, found at 469.3684.

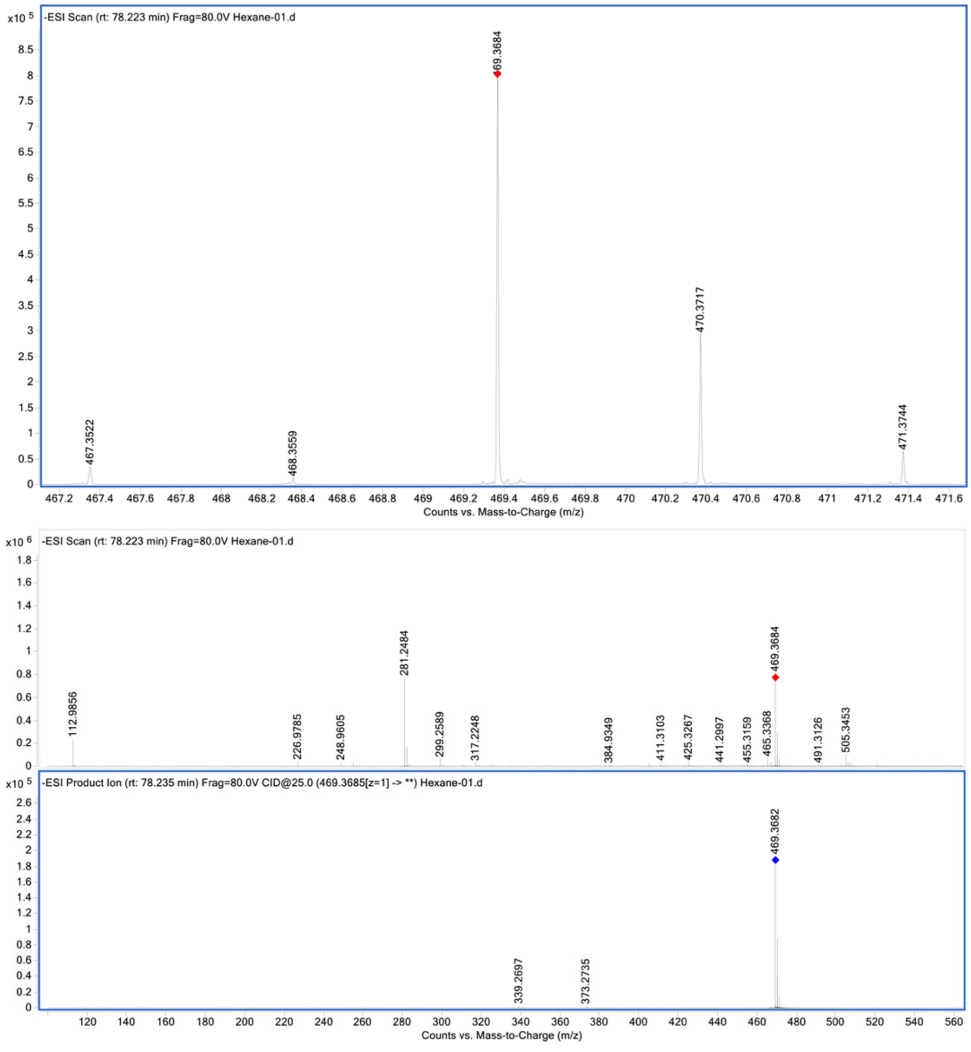

Compound 15, Antcin N, (2*S*,6*R*)-2-methyl-3-methylene-6-((3*R*,4*S*,5*S*,7*S*,10*S*,12*R*,13*R*,14*R*,17*R*)-3,7,12-trihydroxy-4,10,13-trimethyl-11-oxo-2,3,4,5,6,7,10,11,12,13,14,15,16,17-tetradecahydro-1*H*-cyclopenta[*a*]phenanthren-17-yl)heptanoic acid.

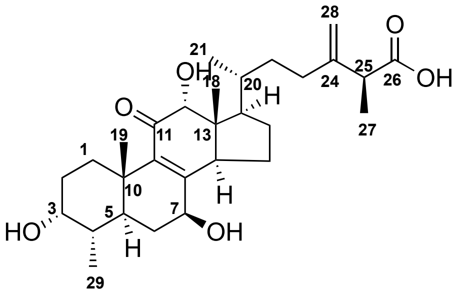

^{1}H NMR (400 MHz, DMSO-d_6) δ 4.85 (s, 1H), 4.82 (s, 1H), 4.09 (t, *J* = 8.5 Hz, 1H), 3.68 (s, 1H), 3.17 (s, 1H), 3.07 – 3.01 (m, 1H), 2.86 (dd, *J* = 12.9, 6.1 Hz, 1H), 2.75 – 2.58 (m, 1H), 2.58 (s, 1H), 2.39 (d, *J* = 13.4 Hz, 1H), 2.31 – 2.20 (m, 1H), 2.16 – 2.04 (m, 4H), 1.98 – 1.91 (m, 2H), 1.84 – 1.73 (m, 2H), 1.64 – 1.27 (m, 9H), 1.15 (dd, *J* = 10.6, 4.7 Hz, 9H), 0.94 – 0.82 (m, 6H), 0.63 (d, *J* = 17.8 Hz, 3H). ^{13}C NMR (126 MHz, DMSO-d_6) δ 175.75, 153.22, 149.57, 140.01, 110.49, 80.59, 69.11, 54.00, 53.22, 49.82, 46.28, 45.61, 45.36, 44.84, 42.50, 36.70, 35.60, 34.52, 34.18, 31.81, 31.11, 30.02, 29.74, 28.81, 27.07, 24.94, 24.63, 18.72, 18.05, 17.90, 17.43, 16.90, 16.85, 16.67, 12.41, 11.92. HRMS (ESI-TOF) calcd for $C_{29}H_{44}O_6$ = 487.3065, found at 487.3061.

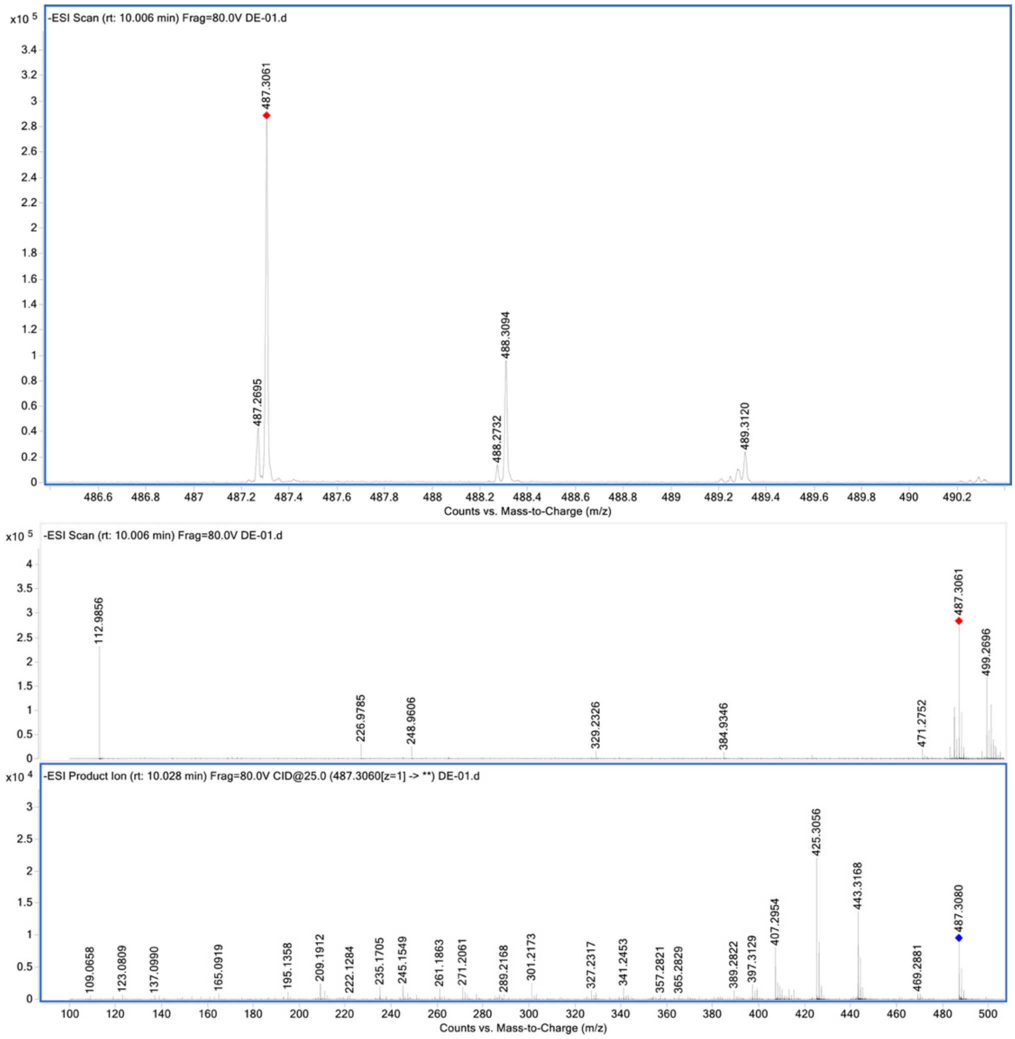

Compound 16, Antcamphin E. (2*S*,6*R*)-6-((4*R*,5*R*,10*S*,13*R*,14*R*,17*R*)-4,7-dihydroxy-4,10,13-trimethyl-3,11-dioxo-2,3,4,5,6,7,10,11,12,13,14,15,16,17-tetradecahydro-1*H*-cyclopenta[*a*]phenanthren-17-yl)-2-methyl-3-methyleneheptanoic acid.

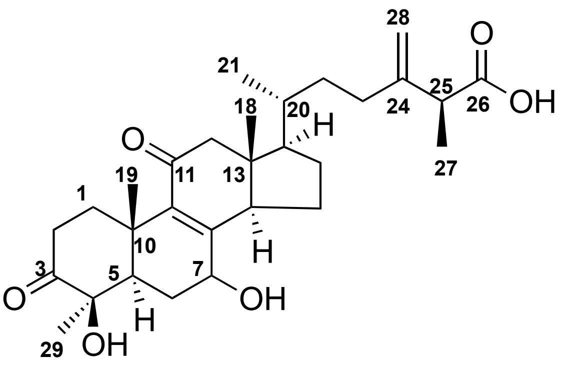

^{1}H NMR (400 MHz, DMSO-d_6) δ 4.84 (s, 1H), 4.81 (s, 1H), 4.15 (t, *J* = 8.3 Hz, 1H), 3.71 (s, 1H), 3.17 (s, 1H), 3.02 (q, *J* = 7.0 Hz, 2H), 2.87 (dd, *J* = 12.7, 6.2 Hz, 1H), 2.68 (ddd, *J* = 13.0, 6.6, 2.6 Hz, 1H), 2.35 (dq, *J* = 13.0, 6.5 Hz, 1H), 2.18 – 1.75 (m, 8H), 1.60 – 1.21 (m, 9H), 1.20 – 1.03 (m, 6H), 0.91 (d, *J* = 6.4 Hz, 6H), 0.63 (s, 3H). ^{13}C NMR (126 MHz, DMSO-d_6) δ 211.98, 175.81, 154.42, 138.16, 110.55, 80.38, 68.64, 49.79, 48.06, 46.20, 45.73, 45.47, 44.86, 43.58, 37.71, 36.52, 35.64, 35.61, 34.18, 32.96, 31.25, 27.06, 24.58, 18.08, 17.96, 16.90, 16.72, 11.87. HRMS (ESI-TOF) calcd for $C_{29}H_{42}O_6$ = 485.2909, found at 485.2903.

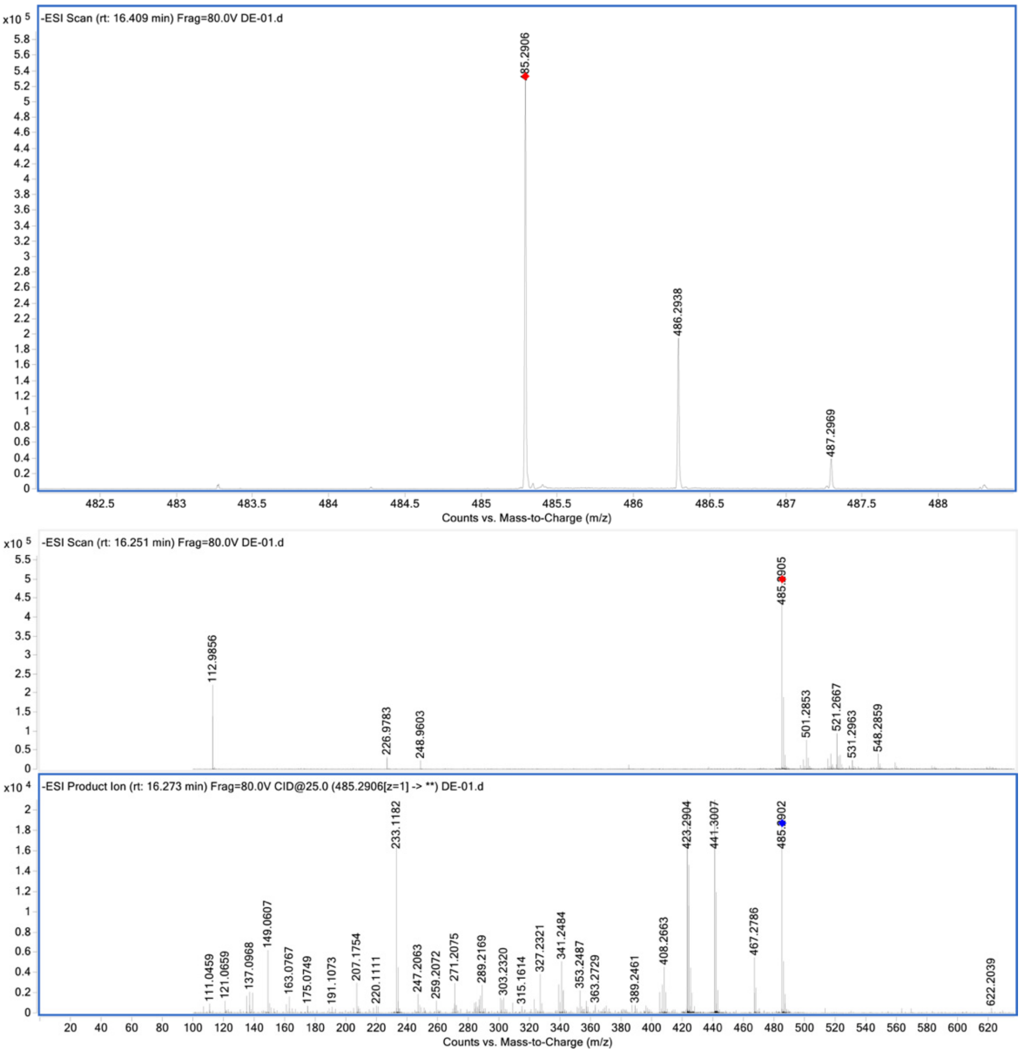

Compound 17, Methyl antcinate K*. methyl (2*R*,6*R*)-2-methyl-3-methylene-6-((3*R*,4*R*,5*R*,7*S*,10*S*,13*R*,14*R*,17*R*)-3,4,7-trihydroxy-4,10,13-trimethyl-11-oxo-2,3,4,5,6,7,10,11,12,13,14,15,16,17-tetradecahydro-1*H*-cyclopenta[*a*]phenanthren-17-yl)heptanoate.

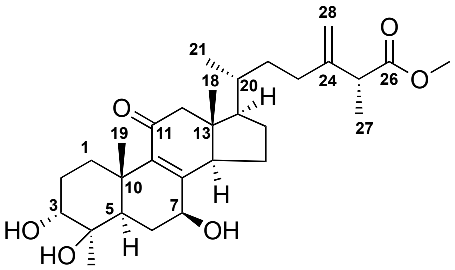

^{1}H NMR (400 MHz, DMSO-d_6) δ 4.83 (s, 1H), 4.79 (s, 1H), 4.14 (t, *J* = 5.9 Hz, 1H), 2.99 (d, *J* = 7.2 Hz, 2H), 2.69 (dd, *J* = 8.7, 3.6 Hz, 1H), 2.66 – 2.56 (m, 2H), 2.51 (s, 6H), 2.45 – 2.36 (m, 2H), 2.10 (d, *J* = 10.5 Hz, 6H), 1.97 – 1.75 (m, 5H), 1.58 – 1.46 (m, 3H), 1.43 – 1.31 (m, 4H), 1.21 (s, 4H), 1.14 (d, *J* = 6.3 Hz, 6H), 0.88 (d, *J* = 5.3 Hz, 6H), 0.71 (s, 3H). HRMS (ESI-TOF) calcd for $C_{30}H_{46}O_6$ = 501.3222, found at 501.2854.

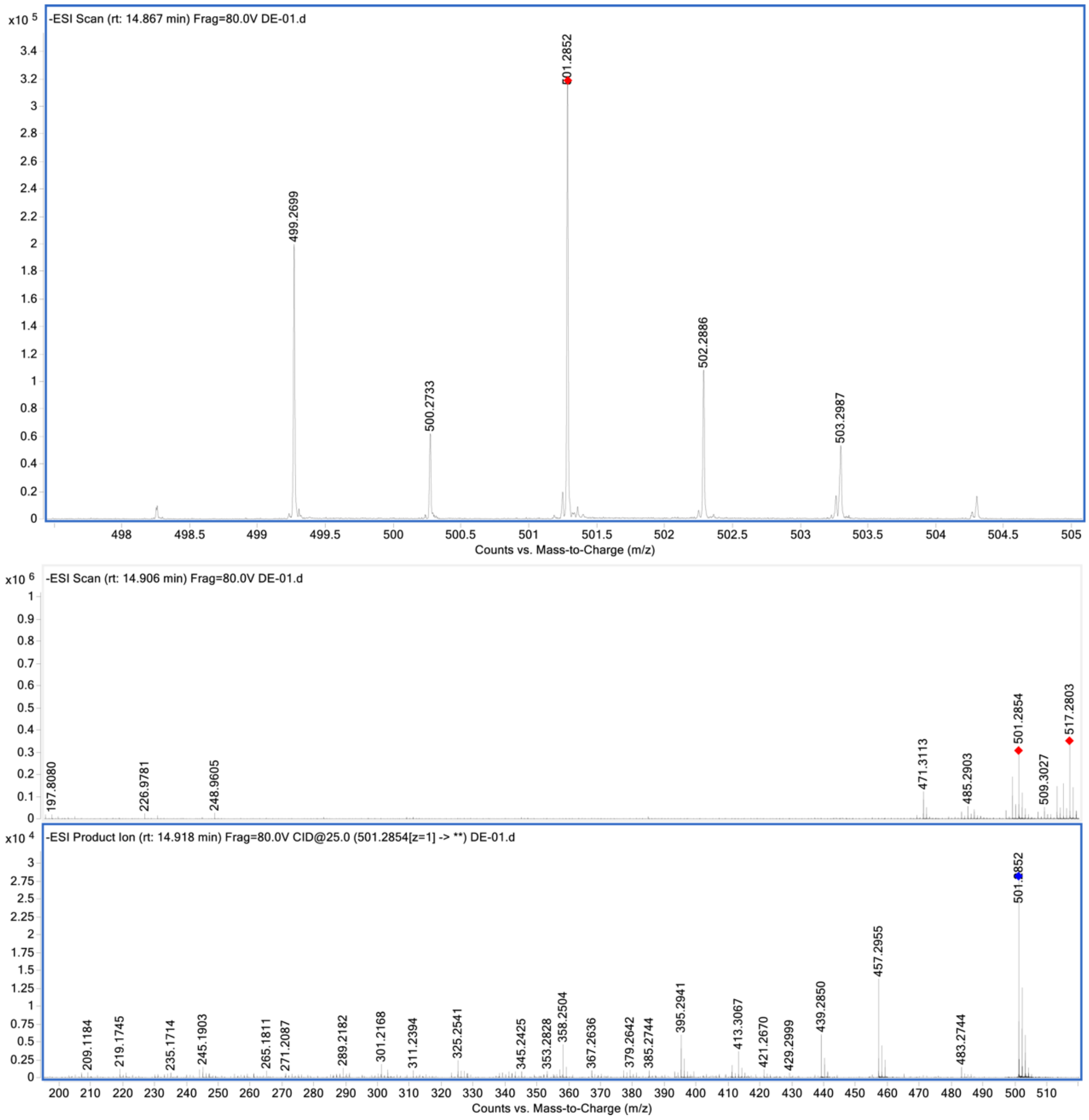

Compound 18, Camphoratin D*. (2*R*,6*R*)-6-((4*S*,7*S*,10*S*,13*R*,14*S*,17*R*)-7,14-dihydroxy-4,10,13-trimethyl-3,11-dioxo-2,3,4,5,6,7,10,11,12,13,14,15,16,17-tetradecahydro-1*H*-cyclopenta[*a*]phenanthren-17-yl)-2-methyl-3-methyleneheptanoic acid

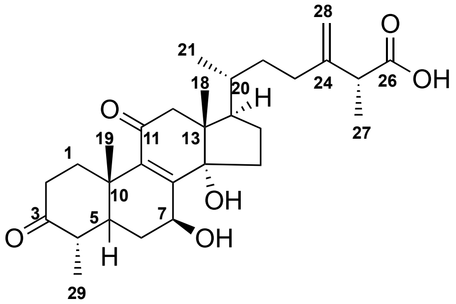

^{1}H NMR (500 MHz, DMSO-d_6) δ 4.85 (s, 1H), 4.79 (s, 1H), 3.08 (s, 1H), 3.03 (t, J = 7.0 Hz, 1H), 2.75 – 2.34 (m, 4H), 2.31 – 2.00 (m, 5H), 1.99 – 1.82 (m, 3H), 1.80 – 1.65 (m, 1H), 1.61 – 1.47 (m, 1H), 1.47 – 0.99 (m, 10H), 0.88 (dt, J = 5.8, 2.6 Hz, 3H), 0.78 – 0.56 (m, 3H). ^{13}C NMR (126 MHz, DMSO-d_6) δ 210.03, 200.28, 175.73, 175.14, 155.85, 149.56, 149.40, 137.34, 110.68, 110.56, 66.17, 57.03, 54.42, 52.29, 50.70, 45.96, 45.57, 45.33, 38.47, 35.64, 35.57, 33.97, 33.88, 33.18, 31.55, 31.29, 31.15, 29.71, 27.92, 24.06, 22.79, 18.68, 16.89, 16.69, 12.69. HRMS (ESI-TOF) calcd for $C_{29}H_{42}O_6$ = 485.2909, found at 485.2906.

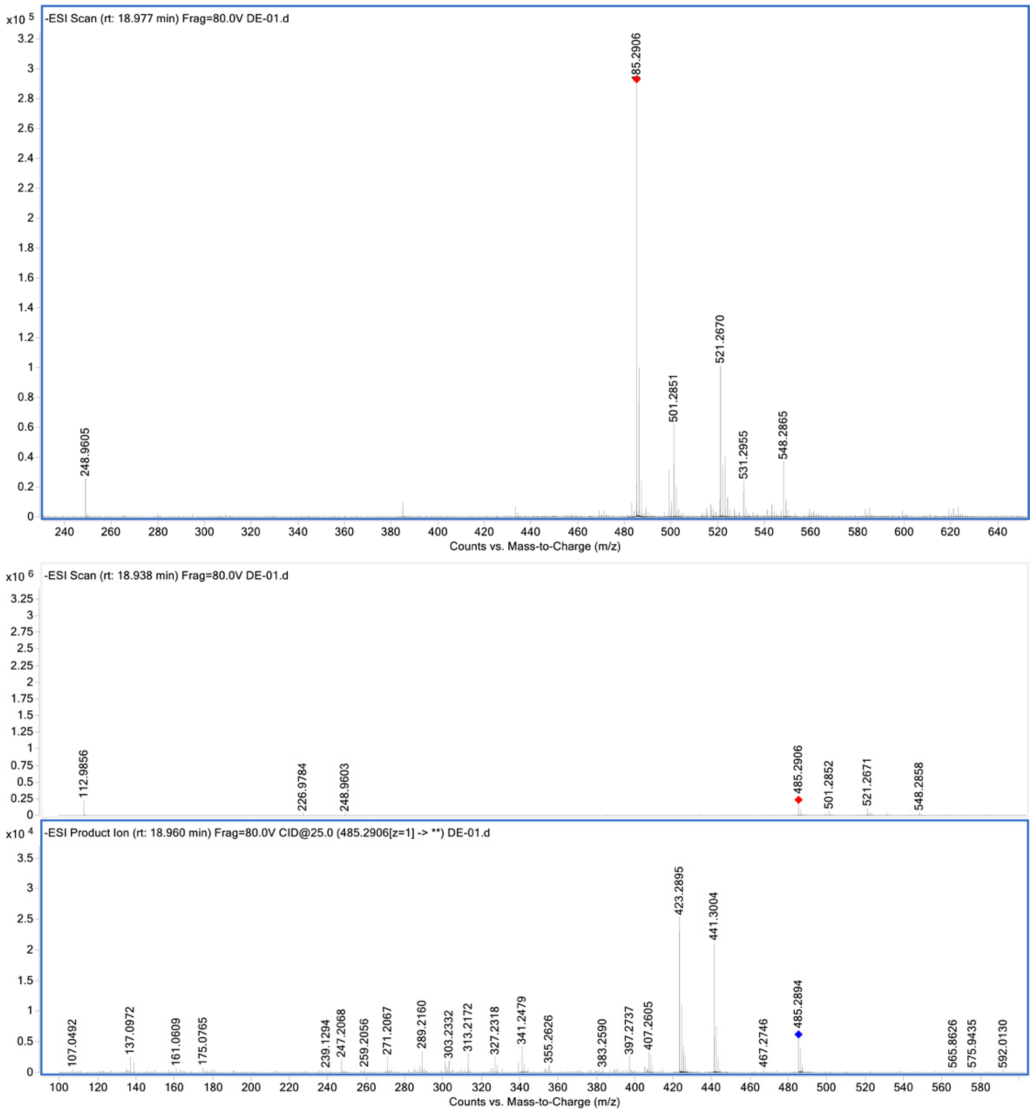

Compound 19, (25S)-Antcin H. (2*S*,6*R*)-6-((4*S*,10*S*,13*R*,14*R*,17*R*)-3,12-dihydroxy-4,10,13-trimethyl-7,11-dioxo-2,3,4,5,6,7,10,11,12,13,14,15,16,17-tetradecahydro-1*H*-cyclopenta[*a*]phenanthren-17-yl)-2-methyl-3-methyleneheptanoic acid.

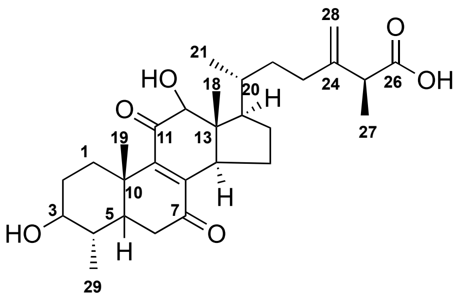

^{1}H NMR (400 MHz, DMSO-d_6) δ 4.98 (s, 1H), 4.85 (s, 1H), 4.82 (s, 1H), 4.23 (t, *J* = 8.5 Hz, 1H), 3.01 (t, *J* = 7.0 Hz, 1H), 2.67 (ddt, *J* = 56.6, 32.2, 12.2 Hz, 4H), 2.37 (dd, *J* = 14.4, 6.3 Hz, 1H), 2.23 (dd, *J* = 13.7, 6.1 Hz, 1H), 2.10 (d, *J* = 10.6 Hz, 8H), 1.62 – 1.05 (m, 16H), 0.93 – 0.82 (m, 3H), 0.67 (s, 3H). ^{13}C NMR (126 MHz, DMSO-d_6) δ 213.91, 201.03, 175.74, 155.16, 149.30, 110.49, 75.68, 69.63, 57.92, 54.12, 52.92, 49.83, 47.32, 45.28, 36.94, 36.19, 35.67, 33.92, 33.80, 31.27, 29.41, 27.87, 24.93, 23.72, 20.14, 18.52, 16.49, 12.28. HRMS (ESI-TOF) calcd for $C_{29}H_{42}O_6$ = 485.2909, found at 485.2903.

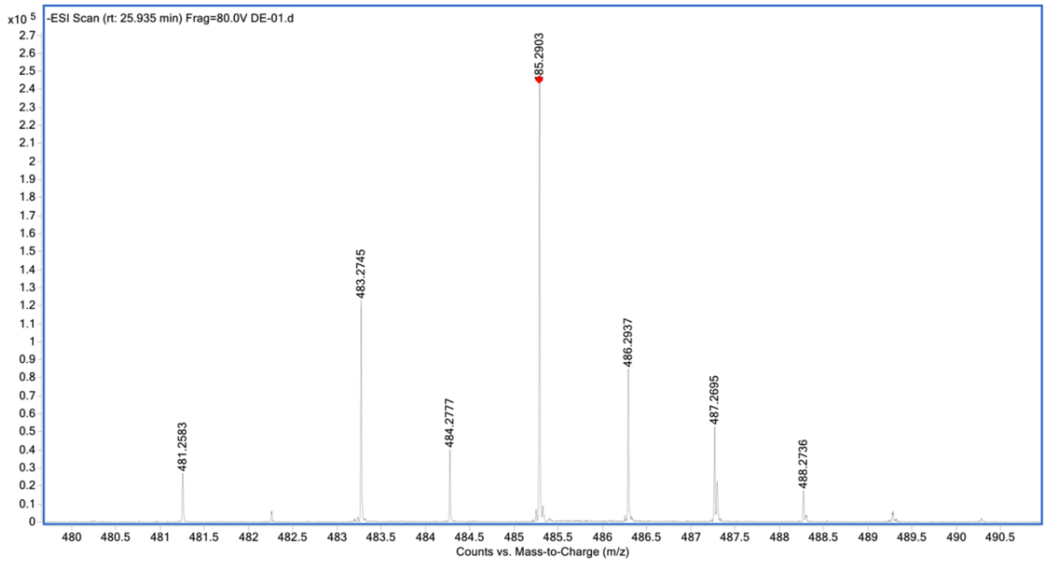

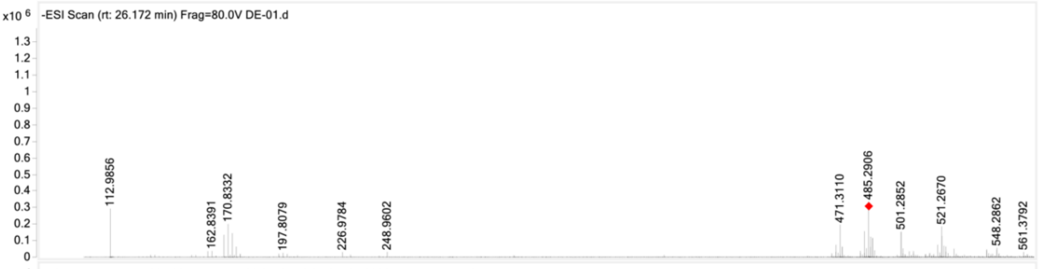

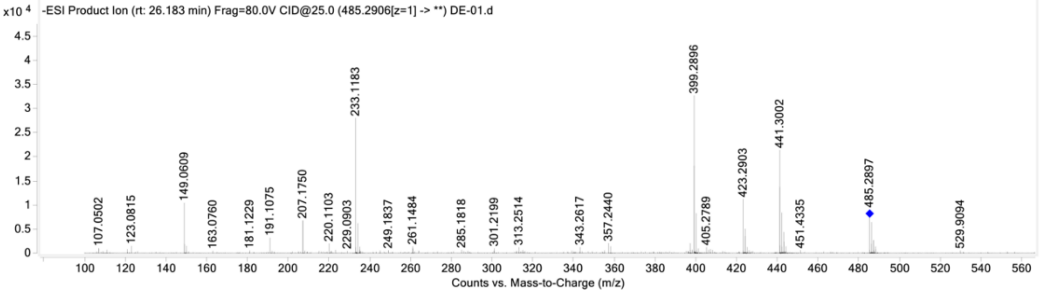

Compound 20, (25R)-Antcin H. (2*R*,6*R*)-6-((4*S*,10*S*,13*R*,14*R*,17*R*)-3,12-dihydroxy-4,10,13-trimethyl-7,11-dioxo-2,3,4,5,6,7,10,11,12,13,14,15,16,17-tetradecahydro-1*H*-cyclopenta[*a*]phenanthren-17-yl)-2-methyl-3-methyleneheptanoic acid.

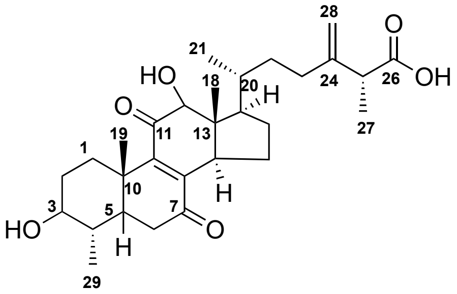

^{1}H NMR (400 MHz, DMSO-d_6) δ 4.98 (s, 1H), 4.84 (s, 1H), 4.81 (s, 0H), 4.23 (t, *J* = 8.6 Hz, 1H), 3.03 (t, *J* = 7.3 Hz, 1H), 2.67 (ddt, *J* = 56.7, 32.6, 12.5 Hz, 3H), 2.37 (d, *J* = 13.9 Hz, 1H), 2.31 – 2.17 (m, 1H), 2.06 (q, *J* = 13.6, 12.9 Hz, 3H), 1.98 – 1.65 (m, 3H), 1.65 – 1.20 (m, 7H), 1.14 (d, *J* = 8.7 Hz, 6H), 0.97 – 0.74 (m, 4H), 0.67 (s, 3H). ^{13}C NMR (126 MHz, DMSO-d_6) δ 213.81, 201.00, 175.73, 155.28, 149.47, 141.11, 110.55, 75.72, 69.61, 57.98, 54.14, 52.93, 49.95, 45.54, 37.00, 36.27, 35.71, 34.03, 31.20, 29.49, 27.90, 24.97, 23.78, 20.28, 18.75, 16.86, 12.48. HRMS (ESI-TOF) calcd for $C_{29}H_{42}O_6$ = 485.2909, found at 485.2902.

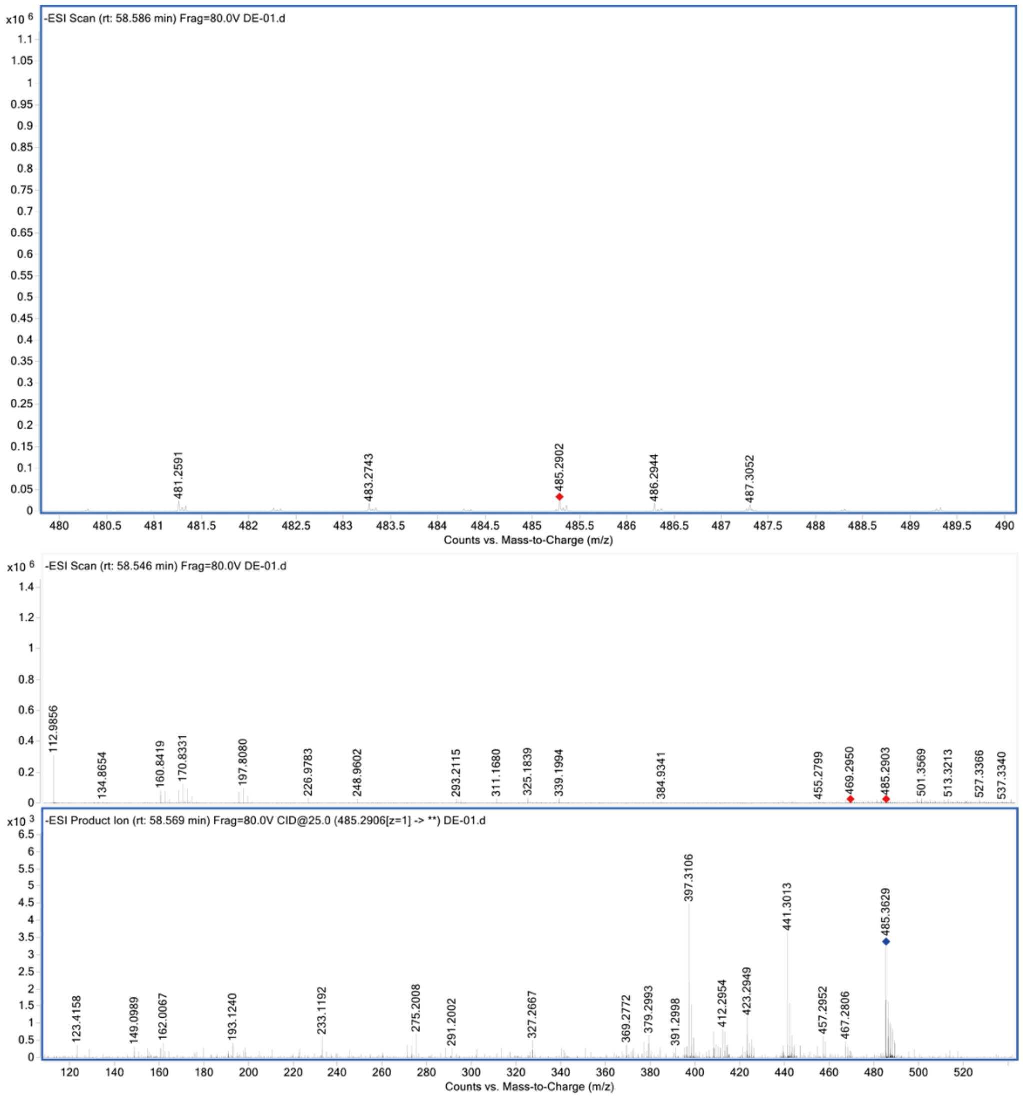

Compound 21, Camphoratin G. (2*R*,6*R*)-6-((4*S*,7*R*,10*S*,13*R*,14*R*,17*R*)-7-hydroxy-4,10,13-trimethyl-3,11-dioxo-2,3,4,5,6,7,10,11,12,13,14,15,16,17-tetradecahydro-1*H*-cyclopenta[*a*]phenanthren-17-yl)-2-methyl-3-methyleneheptanoic acid.

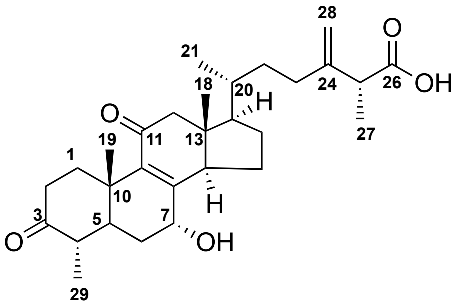

^{1}H NMR (400 MHz, DMSO-d_6) δ 4.85 (s, 1H), 4.82 (s, 1H), 4.18 (t, *J* = 8.4 Hz, 1H), 3.03 (q, *J* = 7.1 Hz, 1H), 2.82 (ddt, *J* = 9.4, 7.0, 2.6 Hz, 1H), 2.74 – 2.53 (m, 3H), 2.50 – 2.28 (m, 2H), 2.16 – 1.69 (m, 7H), 1.60 – 1.08 (m, 13H), 0.89 (dd, *J* = 11.7, 6.0 Hz, 6H), 0.64 (d, *J* = 40.6 Hz, 3H). ^{13}C NMR (126 MHz, DMSO-d_6) δ 212.01, 175.69, 155.87, 149.30, 110.73, 68.75, 57.98, 54.06, 52.95, 48.19, 47.61, 45.29, 43.61, 37.75, 37.01, 35.76, 35.68, 35.52, 33.94, 32.75, 31.31, 27.90, 24.85, 18.71, 17.65, 16.85, 16.67, 12.50, 12.15, 11.91. HRMS (ESI-TOF) calcd for $C_{29}H_{42}O_5$ = 469.2959, found at 469.2957.

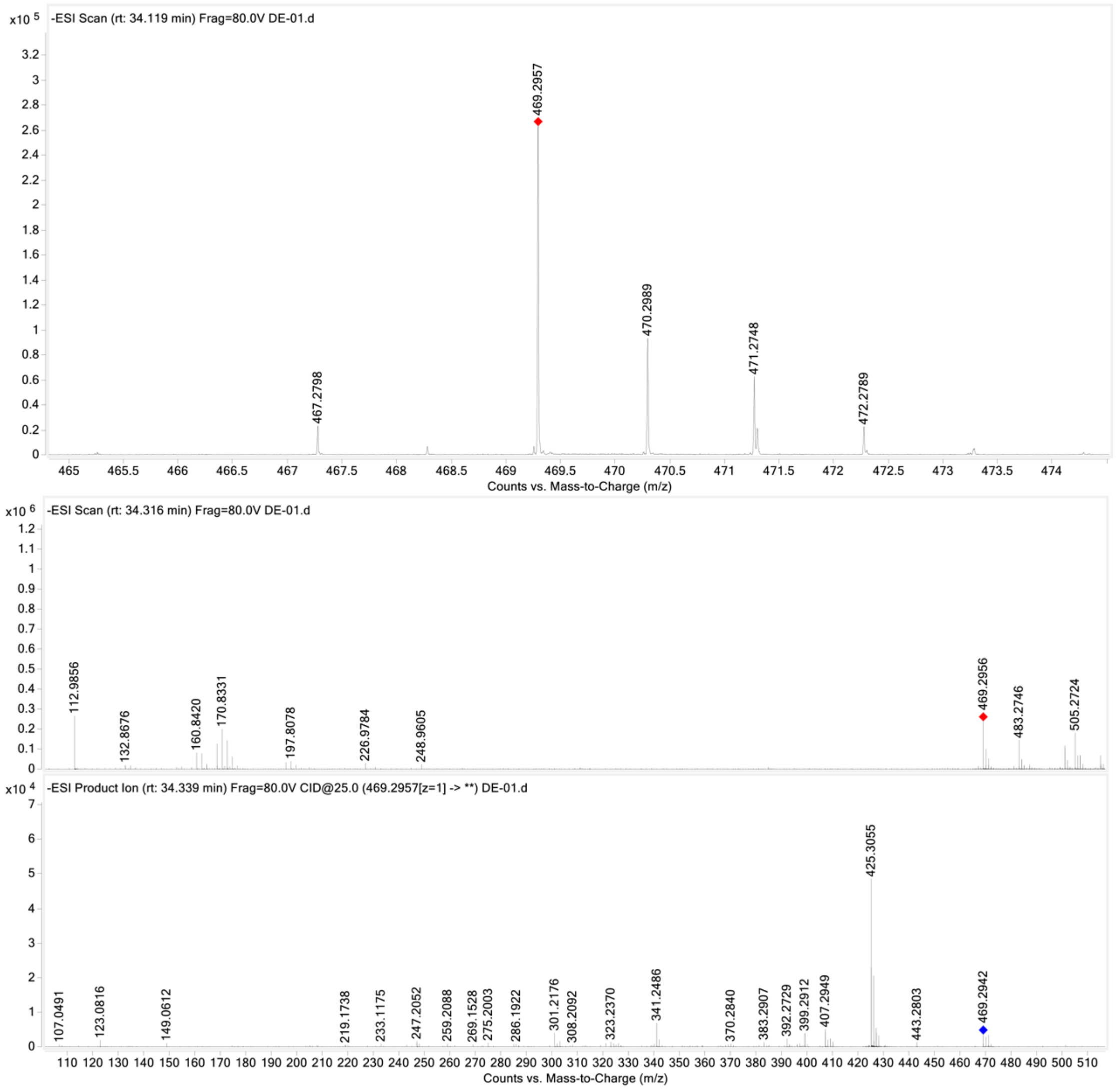

Compound 22, (25R)-Antcin C**,** (2*R*,6*R*)-6-((4*S*,7*S*,10*S*,13*R*,14*R*,17*R*)-7-hydroxy-4,10,13-trimethyl-3,11-dioxo-2,3,4,5,6,7,10,11,12,13,14,15,16,17-tetradecahydro-1*H*-cyclopenta[*a*]phenanthren-17-yl)-2-methyl-3-methyleneheptanoic acid:

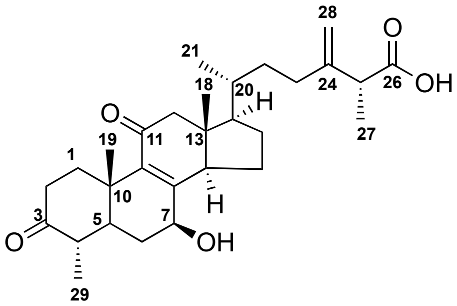

^{1}H NMR (500 MHz, DMSO-d_6) δ 4.85 (s, 1H), 4.82 (s, 1H), 4.18 (t, *J* = 8.5 Hz, 1H), 3.10 – 2.96 (m, 1H), 2.82 (dd, *J* = 12.8, 6.6 Hz, 1H), 2.68 (dd, *J* = 12.8, 6.6 Hz, 1H), 2.59 (d, *J* = 13.9 Hz, 1H), 2.55 – 2.29 (m, 4H), 2.28-2.07 (m, 4H), 1.99 – 1.90 (m, 1H), 1.90 – 1.73 (m, 2H), 1.68 – 1.02 (m, 14H), 0.88 (dt, *J* = 13.8, 7.4 Hz, 6H), 0.68 (s, 3H). ^{13}C NMR (126 MHz, DMSO) δ 211.88, 201.19, 175.67, 155.81, 149.31, 140.23, 110.66, 68.75, 58.00, 54.11, 52.97, 48.22, 47.60, 45.30, 43.60, 37.75, 37.01, 35.77, 35.70, 33.96, 32.75, 31.33, 27.91, 24.85, 18.75, 18.71, 17.62, 16.86, 16.67, 12.48, 11.89. HRMS (ESI-TOF) calcd for $C_{29}H_{42}O_5$ = 469.2959, found 469.3319.

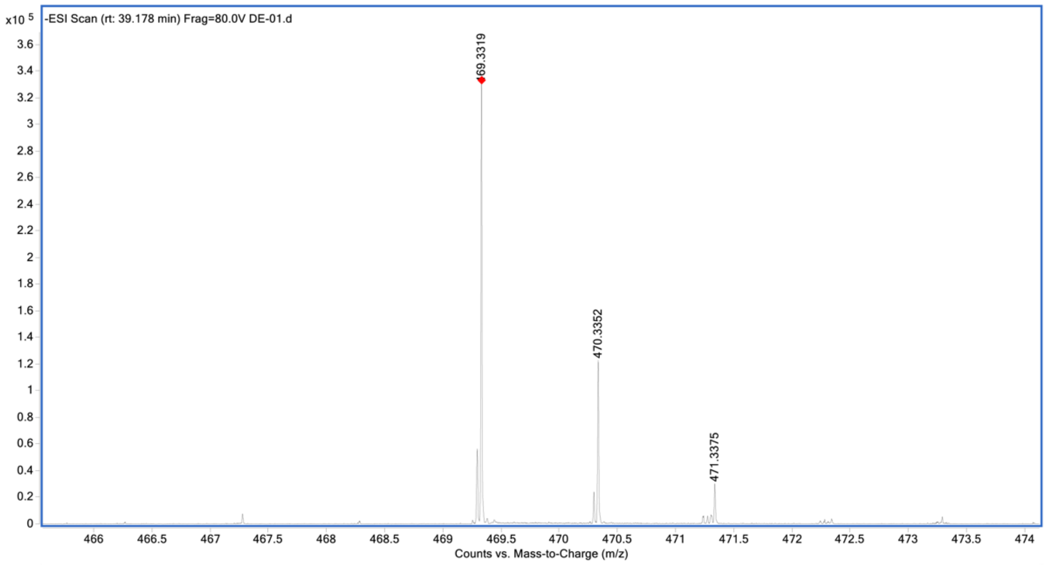

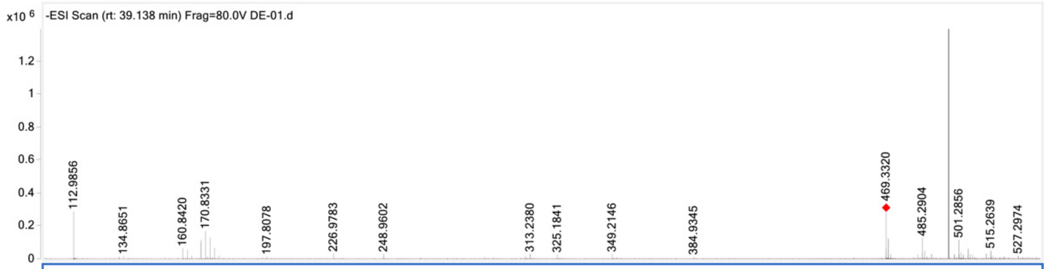

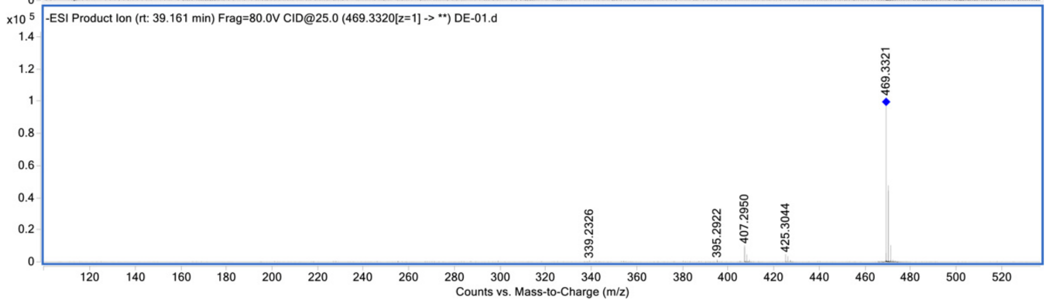

Compound 23, (25S)-Antcin C. (2*S*,6*R*)-6-((4*S*,7*S*,10*S*,13*R*,14*R*,17*R*)-7-hydroxy-4,10,13-trimethyl-3,11-dioxo-2,3,4,5,6,7,10,11,12,13,14,15,16,17-tetradecahydro-1*H*-cyclopenta[*a*]phenanthren-17-yl)-2-methyl-3-methyleneheptanoic acid:

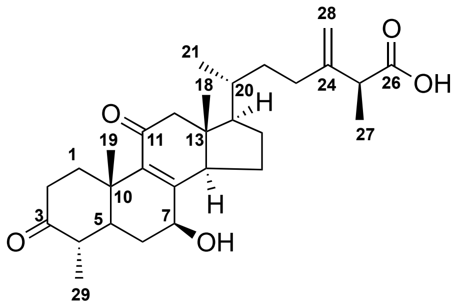

^{1}H NMR (500 MHz, DMSO-d_6) δ 4.84 (s, 1H), 4.81 (s, 1H), 4.17 (t, *J* = 8.5 Hz, 1H), 3.02 (q, *J* = 7.0 Hz, 1H), 2.82 (ddd, *J* = 13.3, 6.6, 2.6 Hz, 1H), 2.68 (dd, *J* = 12.5, 6.3 Hz, 1H), 2.59 (d, *J* = 13.8 Hz, 1H), 2.53 – 2.30 (m, 3H), 2.22 – 1.98 (m, 4H), 1.98 – 1.73 (m, 3H), 1.62 – 1.06 (m, 15H), 0.89 (dd, *J* = 14.6, 5.8 Hz, 6H), 0.68 (s, 3H). ^{13}C NMR (126 MHz, DMSO-d_6) δ 211.82, 201.15, 175.65, 155.81, 149.46, 110.55, 68.75, 58.01, 54.13, 52.98, 48.24, 47.60, 45.51, 43.60, 37.76, 37.01, 35.77, 35.71, 34.04, 32.76, 31.20, 27.87, 24.86, 18.75, 17.62, 16.85, 12.48, 11.89. HRMS (ESI-TOF) calcd for $C_{29}H_{42}O_5$ = 469.2959, found at 469.2953.

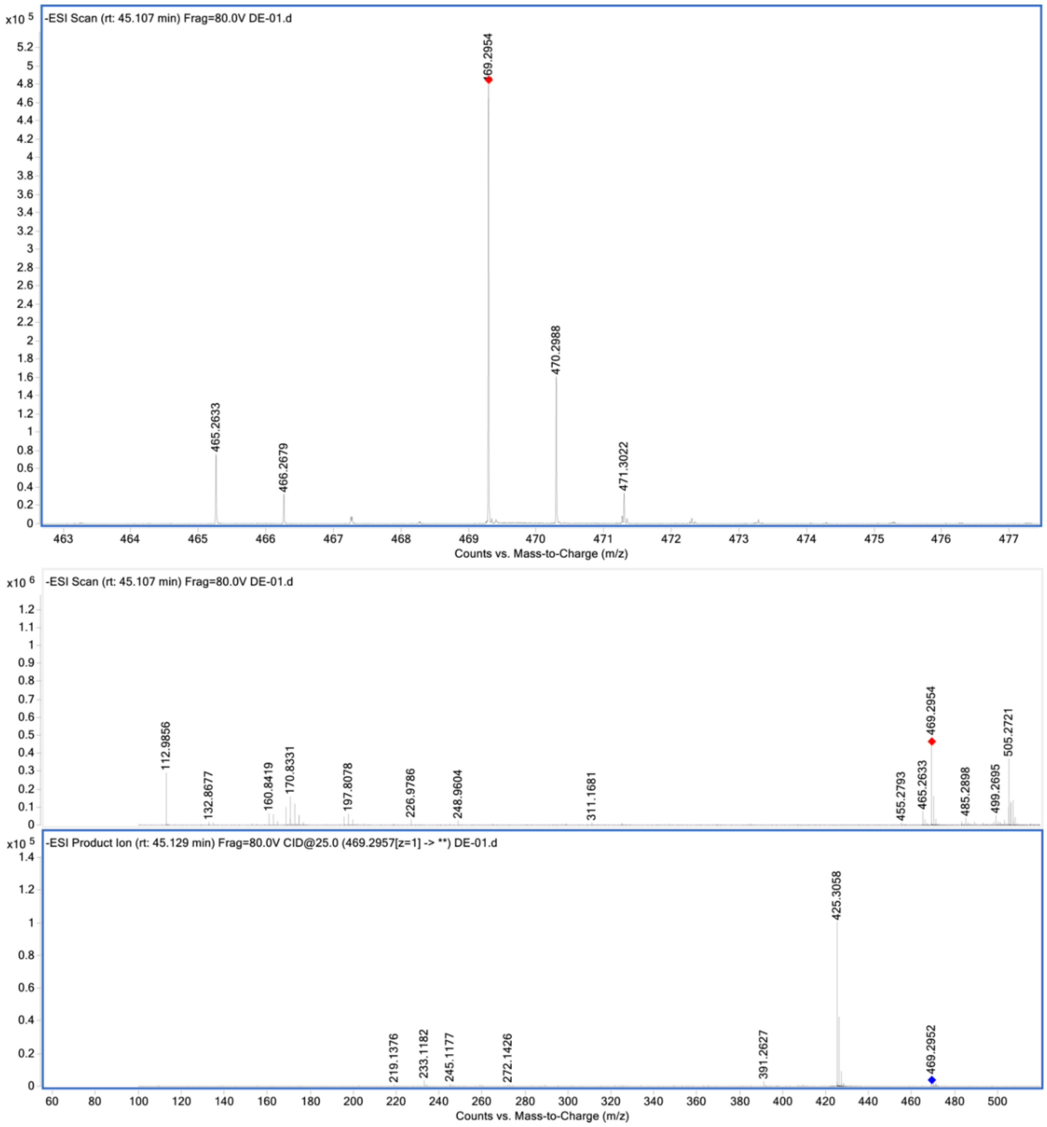

Compound 24, Camphoratin C*. (2*R*,6*R*)-6-((3*R*,4*R*,10*S*,13*R*,14*R*,17*R*)-3,4-dihydroxy-4,10,13-trimethyl-7,11-dioxo-2,3,4,5,6,7,10,11,12,13,14,15,16,17-tetradecahydro-1*H*-cyclopenta[*a*]phenanthren-17-yl)-2-methyl-3-methyleneheptanoic acid:

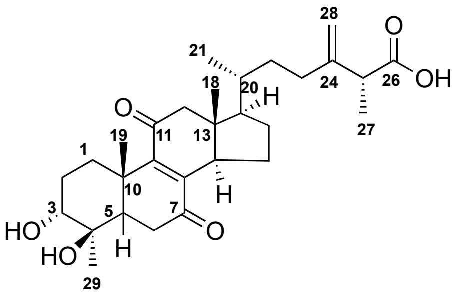

^{1}H NMR (400 MHz, DMSO-d_6) δ 4.84 (s, 1H), 4.81 (s, 1H), 3.83 (s, 2H), 3.61 – 3.52 (m, 1H), 3.02 (q, *J* = 7.1 Hz, 1H), 2.88 (dd, *J* = 12.6, 7.3 Hz, 1H), 2.36 (dd, *J* = 12.1, 5.7 Hz, 1H), 2.28 (d, *J* = *15*.2 Hz, 1H), 2.24 – 2.14 (m, 2H), 2.14 – 1.77 (m, 7H), 1.75 – 1.45 (m, 6H), 1.36 (td, *J* = 14.1, 13.6, 4.2 Hz, 4H), 1.24 (s, 5H), 1.15 (d, *J* = 6.9 Hz, 4H), 0.92 (d, *J* = 6.5 Hz, 5H), 0.82 (d, *J* = 6.6 Hz, 3H), 0.54 (s, 3H). ^{13}C NMR (126 MHz, DMSO-d_6) δ 202.99, 202.03, 175.86, 152.54, 143.53, 110.49, 80.18, 68.54, 49.60, 45.79, 45.53, 45.27, 41.91, 41.06, 38.36, 38.18, 35.32, 34.55, 34.08, 31.14, 29.40, 28.01, 26.92, 24.03, 18.11, 16.87, 16.71, 16.32, 11.51. HRMS (ESI-TOF) calcd for $C_{29}H_{42}O_6$ = 485.2909, found at 485.2906.

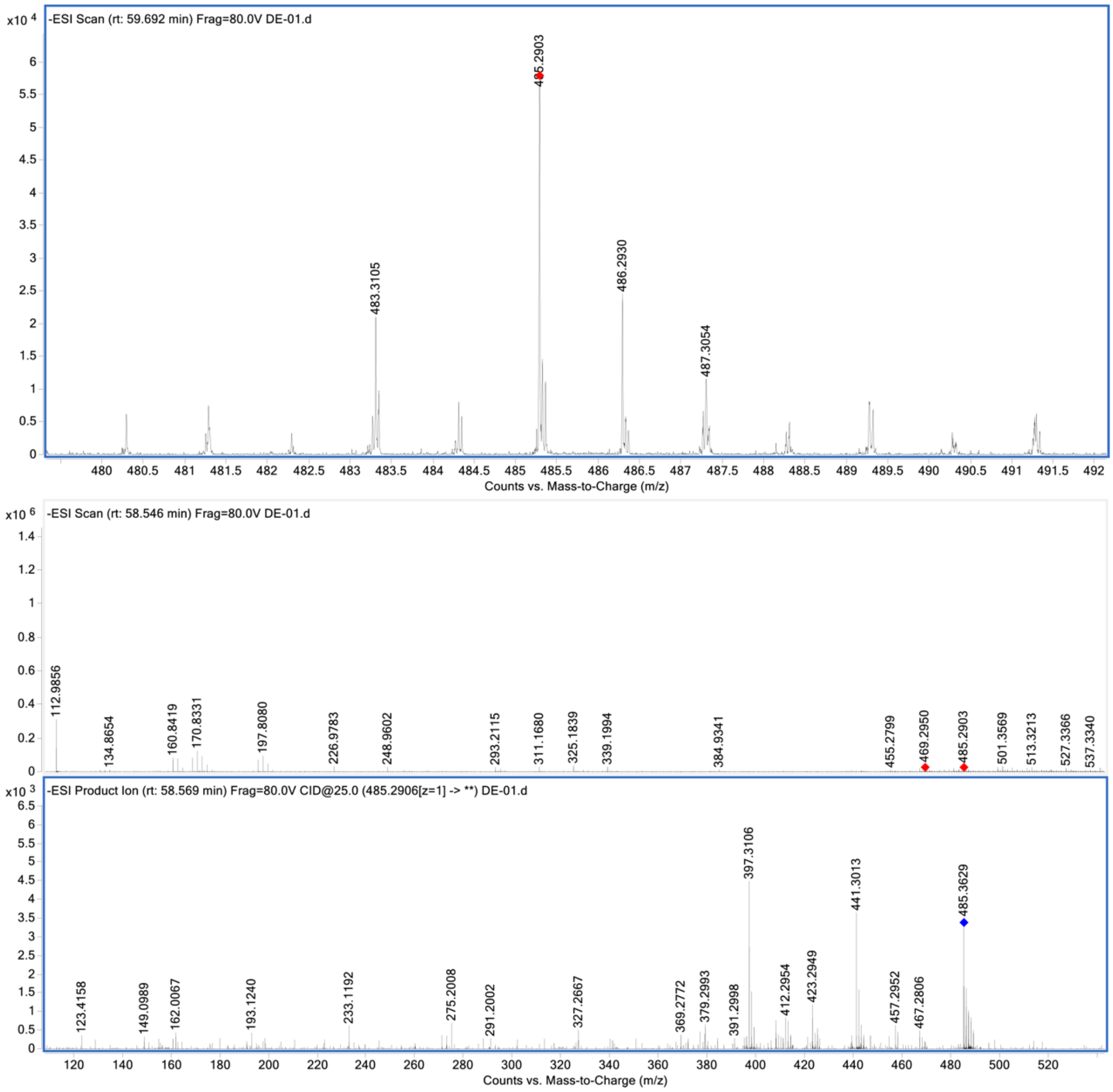

Compound 25, 24-Methylenelanost-8-ene-3β,15α,21-triol. (10*S*,13*R*,14*R*,17*R*)-17-((*R*)-1-hydroxy-6-methyl-5-methyleneheptan-2-yl)-4,4,10,13,14-pentamethyl-2,3,4,5,6,7,10,11,12,13,14,15,16,17-tetradecahydro-1*H*-cyclopenta[*a*]phenanthrene-3,15-diol:

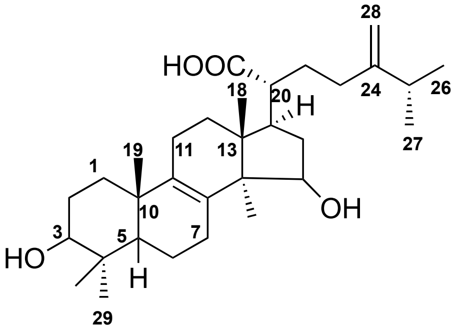

^{1}H NMR (400 MHz, DMSO-d_6) δ 5.88 (d, J = 6.3 Hz, 1H), 5.27 (d, J = 6.1 Hz, 1H), 4.72 (s, 1H), 4.64 (s, 1H), 3.01 (dt, J = 10.4, 4.5 Hz, 1H), 2.26 – 2.14 (m, 1H), 2.06 (td, J = 22.7, 22.1, 9.0 Hz, 4H), 1.97 – 1.85 (m, 3H), 1.78 (q, J = 10.3 Hz, 1H), 1.62 (d, J = 11.8 Hz, 2H), 1.50 (t, J = 11.4 Hz, 4H), 1.24 (s, 2H), 0.96 (q, J = 5.1, 4.3 Hz, 5H), 0.90 (d, J = 4.8 Hz, 5H), 0.79 (d, J = 17.5 Hz, 4H), 0.71 (d, J = 3.9 Hz, 3H), 0.59 (s, 1H). ^{13}C NMR (126 MHz, DMSO-d_6) δ 177.72, 155.26, 134.36, 121.57, 115.59, 107.22, 77.25, 72.74, 71.51, 50.46, 49.24, 47.93, 47.75, 45.78, 45.53, 44.57, 38.96, 38.54, 38.33, 37.08, 35.90, 35.71, 33.71, 31.94, 31.14, 29.49, 28.80, 28.57, 28.00, 26.98, 22.09, 22.00, 20.59, 19.37, 18.37, 17.92, 16.59, 16.48, 16.32. HRMS (ESI-TOF) calcd for $C_{31}H_{50}O_4$ = 485.3636, found at 485.2906.

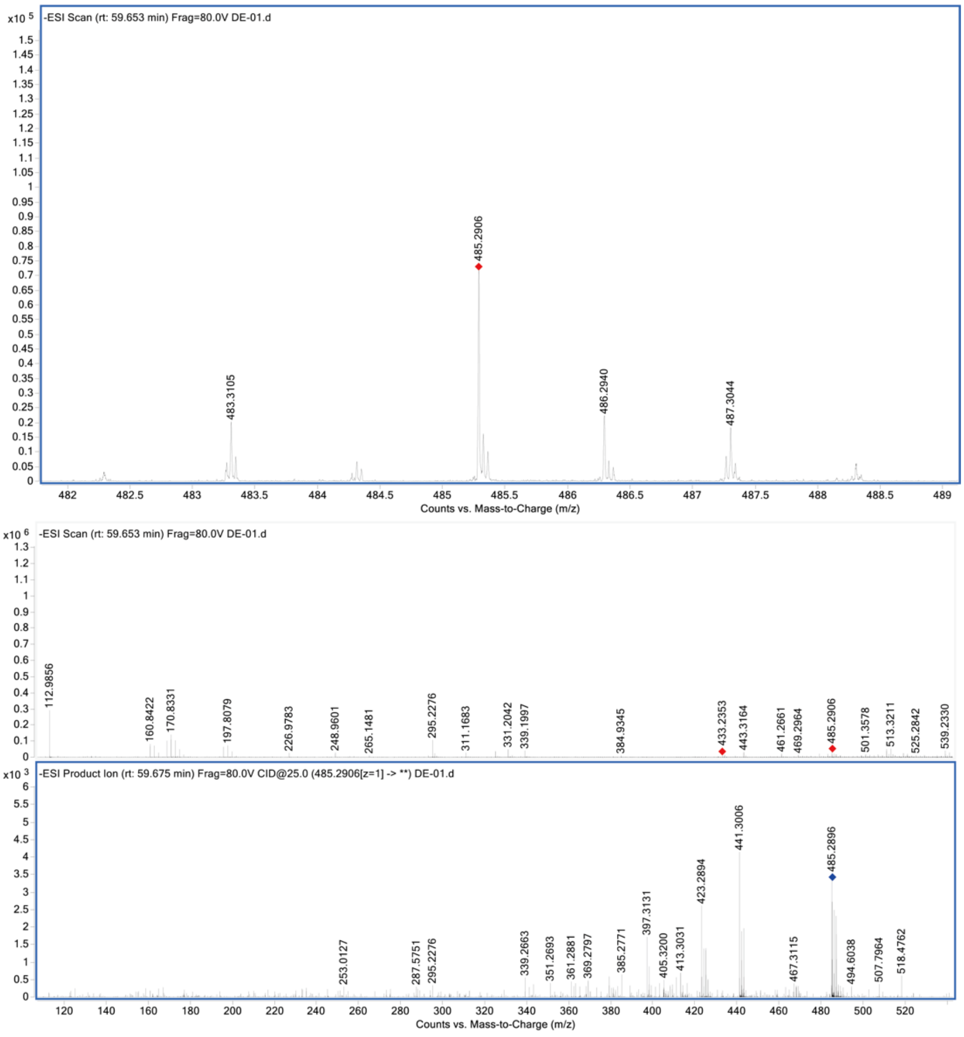

Compound 26, Sulphurenic acid. (2*R*)-2-((10*S*,13*R*,14*R*,17*R*)-3,15-dihydroxy-4,4,10,13,14-pentamethyl-2,3,4,5,6,7,10,11,12,13,14,15,16,17-tetradecahydro-1*H*-cyclopenta[*a*]phenanthren-17-yl)-6-methyl-5-methyleneheptanoic acid:

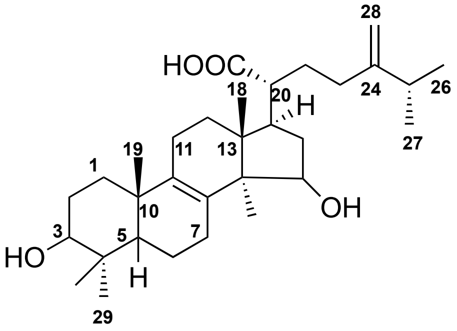

^{1}H NMR (400 MHz, DMSO-d_6) δ 5.28 (s, 1H), 4.83 (d, *J* = 13.7 Hz, 1H), 4.73 (s, 1H), 4.64 (s, 1H), 4.01 (dd, *J* = 9.5, 5.5 Hz, 1H), 3.00 (dd, *J* = 10.4, 5.6 Hz, 2H), 2.28 – 1.85 (m, 13H), 1.73 – 1.42 (m, 11H), 1.25 (d, *J* = 6.4 Hz, 4H), 0.96 (q, *J* = 5.6 Hz, 6H), 0.90 (d, *J* = 8.9 Hz, 7H), 0.82 (s, 3H), 0.77 (s, 1H), 0.71 (d, *J* = 3.7 Hz, 3H), 0.65 (s, 1H), 0.59 (s, 1H). ^{13}C NMR (126 MHz, DMSO-d_6) δ 177.67, 134.43, 134.36, 107.27, 77.25, 71.50, 51.27, 50.47, 47.87, 45.80, 44.58, 38.97, 38.33, 37.09, 35.71, 33.71, 31.93, 31.13, 29.50, 28.58, 28.03, 26.99, 22.09, 22.01, 20.59, 19.38, 18.38, 17.93, 16.48, 16.32, 11.94. HRMS (ESI-TOF) calcd for $C_{31}H_{50}O_4$ = 485.3636, found at 485.2901.

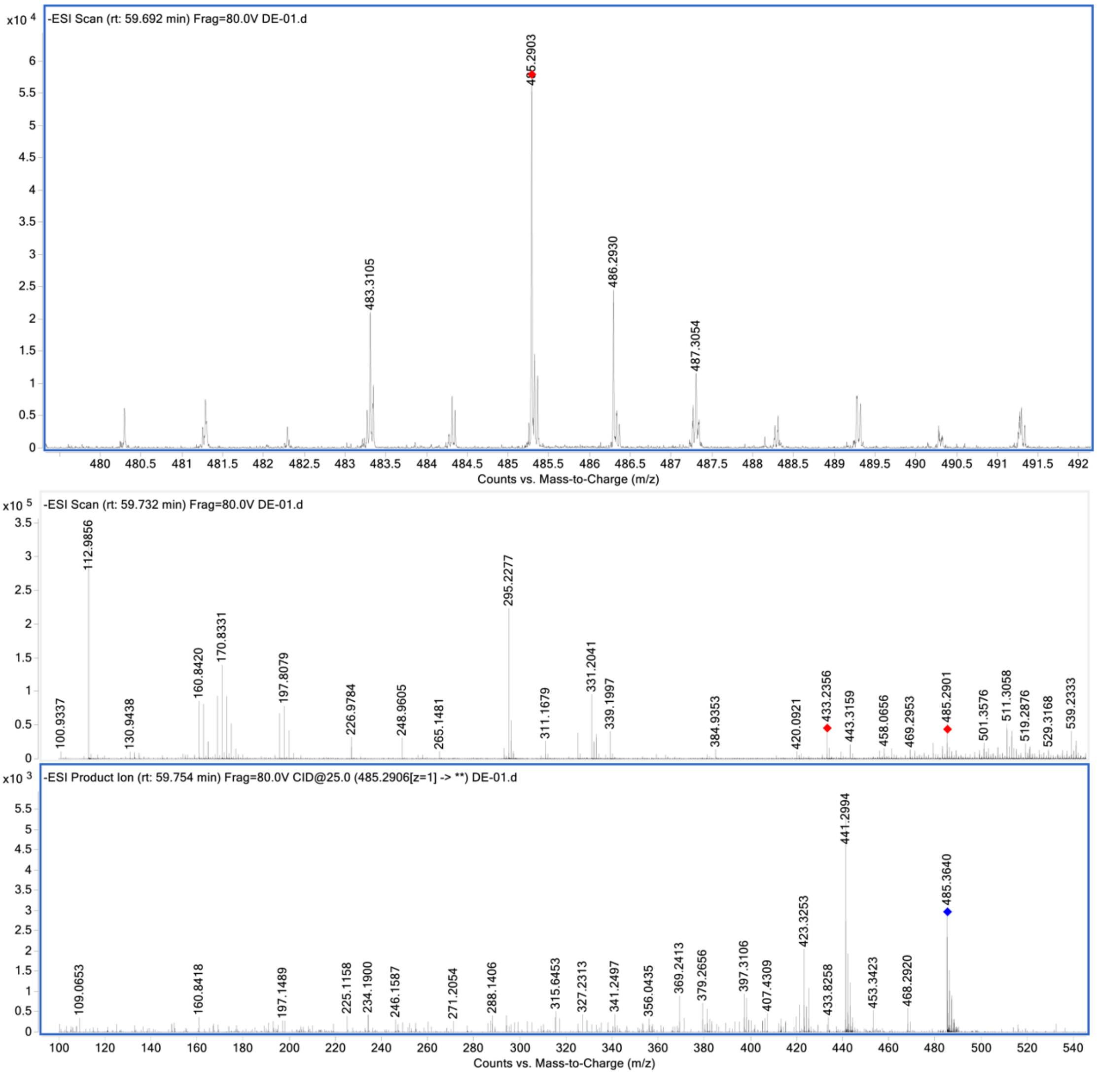

Compound 27, 15α-Acetyl-dehydrosulphurenic acid. (2*R*)-2-((10*S*,13*R*,14*R*,17*R*)-15-acetoxy-3-hydroxy-4,4,10,13,14-pentamethyl-2,3,4,5,6,10,12,13,14,15,16,17-dodecahydro-1*H*-cyclopenta[*a*]phenanthren-17-yl)-6-methyl-5-methyleneheptanoic acid:

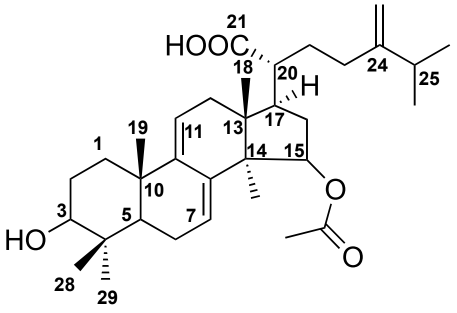

^{1}H NMR (500 MHz, DMSO-d_6) δ 5.49 (d, *J* = 6.5 Hz, 1H), 5.33 (d, *J* = 6.3 Hz, 1H), 4.94 (dd, *J* = 9.6, 5.5 Hz, 1H), 4.72 (s, 1H), 4.64 (s, 1H), 3.01 (dd, *J* = 10.1, 5.6 Hz, 1H), 2.26 – 1.86 (m, 12H), 1.87 – 1.76 (m, 2H), 1.74 – 1.59 (m, 2H), 1.58 – 1.42 (m, 4H), 1.35 – 1.12 (m, 4H), 1.05 – 0.84 (m, 12H), 0.76 (s, 3H), 0.67 (d, *J* = 24.8 Hz, 3H). ^{13}C NMR (126 MHz, DMSO-d_6) δ 155.29, 146.13, 140.41, 121.64, 115.98, 107.21, 77.20, 76.69, 49.06, 47.61, 45.47, 43.98, 38.78, 37.52, 35.90, 35.84, 35.64, 33.69, 31.94, 31.14, 28.81, 27.96, 23.09, 23.01, 22.07, 22.01, 21.52, 18.83, 16.59, 16.26. HRMS (ESI-TOF) calcd for $C_{33}H_{50}O_5$ = 525.3585, found at 525.3577.

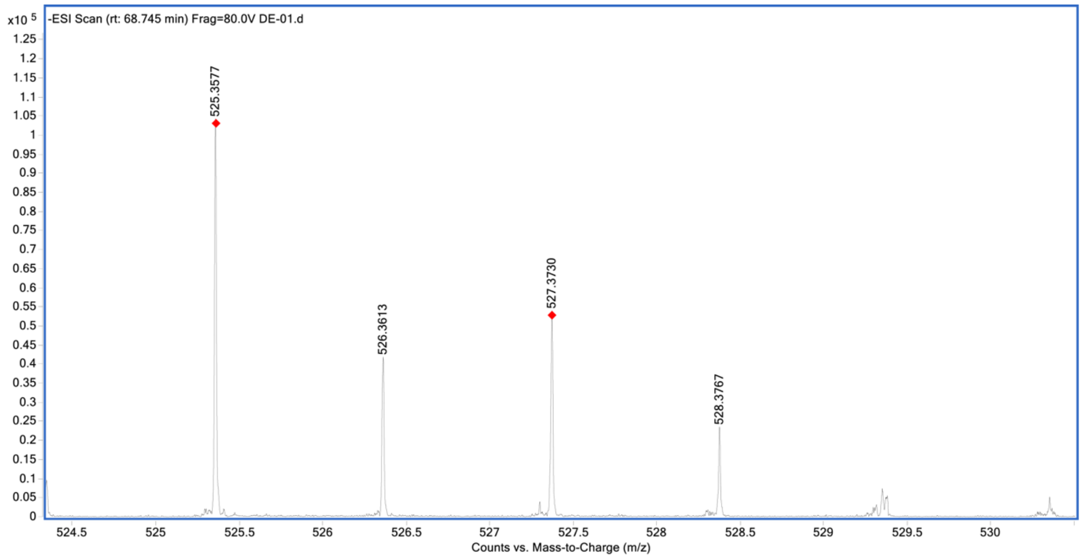

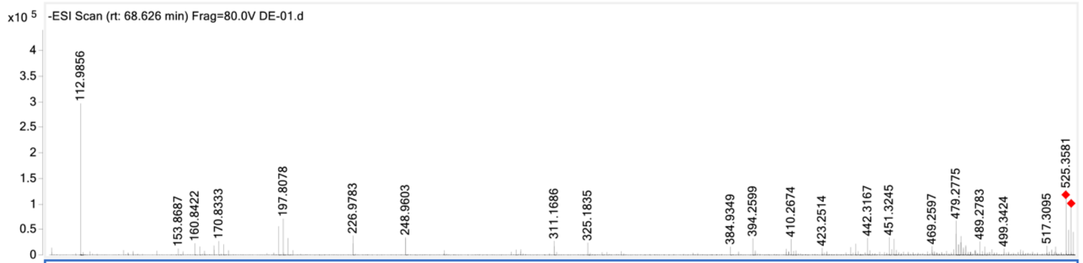

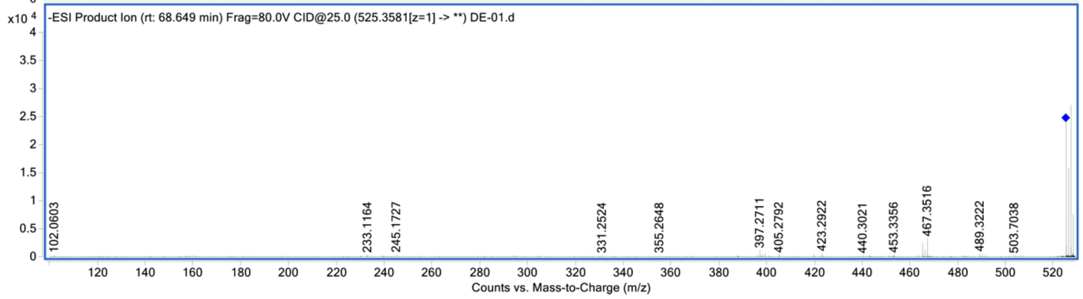

Compound 28, Dehydroeburicoic acid. (2*R*)-2-((10*S*,13*R*,14*R*,17*R*)-3-hydroxy-4,4,10,13,14-pentamethyl-2,3,4,5,6,10,12,13,14,15,16,17-dodecahydro-1*H*-cyclopenta[*a*]phenanthren-17-yl)-6-methyl-5-methyleneheptanoic acid:

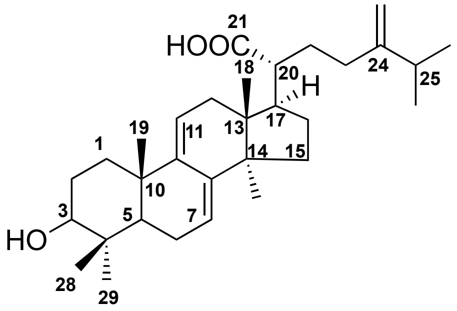

^{1}H NMR (500 MHz, DMSO-d_6) δ 5.48 (d, *J* = 6.3 Hz, 1H), 5.32 (d, *J* = 6.3 Hz, 1H), 4.73 (s, 1H), 4.65 (s, 1H), 3.01 (dd, *J* = 9.9, 5.7 Hz, 1H), 2.23 – 1.90 (m, 13H), 1.82 (dd, *J* = 17.7, 6.4 Hz, 1H), 1.55 (tdd, *J* = 14.1, 10.9, 9.7, 4.1 Hz, 5H), 1.44 – 1.21 (m, 7H), 1.05 – 0.75 (m, 21H), 0.57 (s, 3H). ^{13}C NMR (126 MHz, DMSO-d_6) δ 146.23, 142.44, 120.92, 116.08, 107.27, 77.26, 50.04, 49.34, 48.03, 47.45, 43.63, 38.81, 37.47, 35.87, 35.43, 33.69, 32.04, 31.21, 28.80, 27.99, 26.75, 25.87, 23.11, 23.00, 22.08, 22.00, 16.59, 15.92. HRMS (ESI-TOF) calcd for $C_{31}H_{49}O_3$ = 467.3531, found at 467.3526.

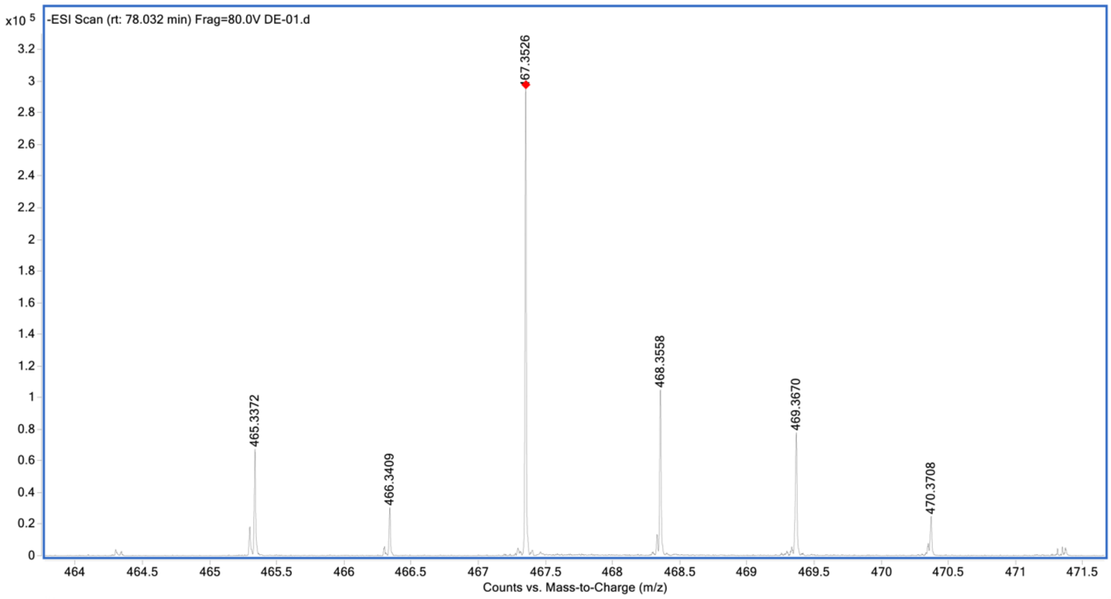

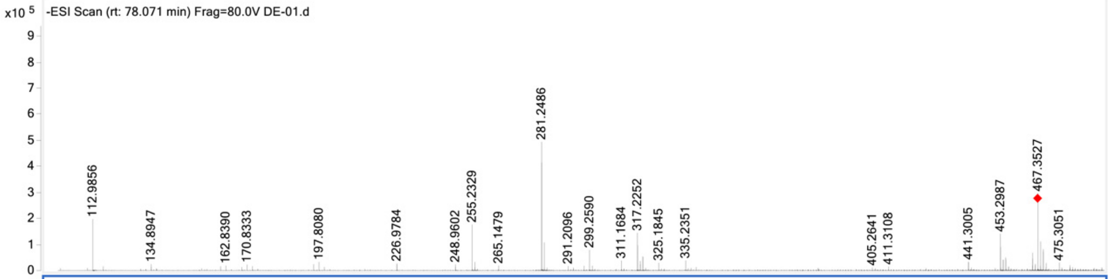

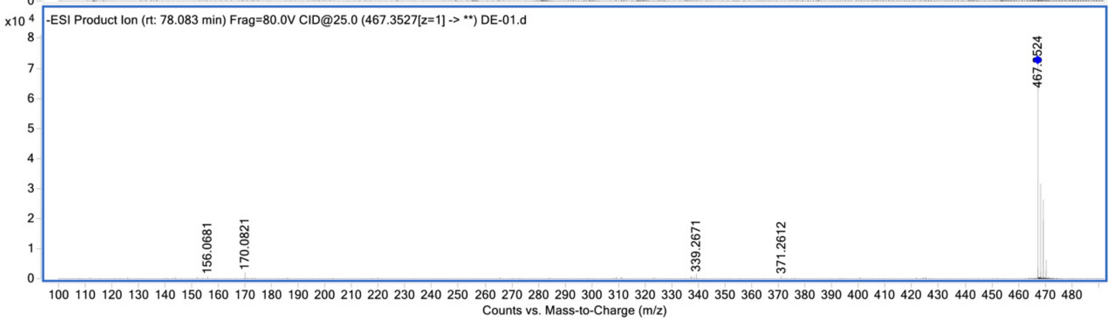

2. X-RAY CRYSTALLOGRAPHIC DATA

a. X-ray crystallographic data for **Compound 5** ((25R)-Antcin K):

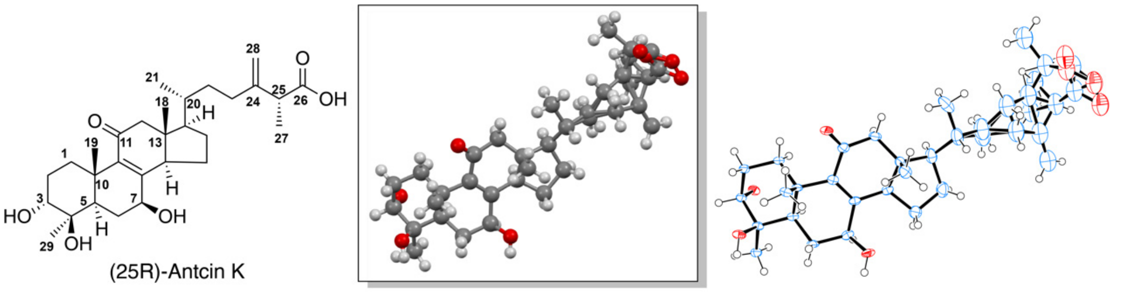

Crystals of **Compound 5**: suitable for X-ray analysis were obtained by slow evaporation from MeOH. A specimen of $C_{29}H_{44}O_6$, approximate dimensions 0.091 mm × 0.199 mm × 0.231 mm, was used for the X-ray crystallographic analysis. The X-ray intensity data were measured (λ = 1.54178 Å).

The total exposure time was 21.07 hours. The frames were integrated with the Bruker SAINT software package using a narrow-frame algorithm. The integration of the data using a monoclinic unit cell yielded a total of 2758 reflections to a maximum θ angle of 66.58° (0.84 Å resolution), of which 2758 were independent (average redundancy 1.000, completeness = 99.8%, R_{int} = 7.85%, R_{sig} = 2.41%) and 2699 (97.86%) were greater than 2σ(F^2). The final cell constants of a = 5.9970 Å, b = 38.7490 Å, c = 6.8193 Å, α = 90.000°, β = 104.368°, γ = 90.000°, volume = 1535.1Å^3, are based upon the refinement of the XYZ-centroids of 9820 reflections above 20 σ(I) with 4.553° < 2θ < 133.0°.Data were corrected for absorption effects using the Multi-Scan method (SADABS). The ratio of minimum to maximum apparent transmission was 0.847.

The structure was solved and refined using the Bruker SHELXTL Software Package, with Z = 2 for the formula unit, $C_{29}H_{49}O_8$. The final anisotropic full-matrix least-squares refinement on F^2 with 417 variables converged at R1 = 10.33%, for the observed data and wR2 = 31.63% for all data. The goodness-of-fit was 1.671. The largest peak in the final difference electron density synthesis was 1.537 e$^-$/Å^3 and the largest hole was -1.110 e$^-$/Å^3 with an RMS deviation of 0.118 e$^-$/Å^3. On the basis of the final model, the calculated density was 1.132 g/cm^3 and F(000), 574 e$^-$.

Crystallographic data have been deposited with the Cambridge Crystallograhic Data Centre (CCDC#2099282). Copies of the data can be obtained free of charge on application to the CCDC, 12 Union Road, Cambridge CB21EZ, UK (fax: (+44)-1223-336-033; e-mail: deposit@ccdc.cam.ac.uk).

Table S1. Sample and crystal data for **Compound 5** ((25R)-Antcin K).

**Identification code**	L178
**Chemical formula**	$C_{29}H_{44}O_6$
**Formula weight**	525.68 g/mol
**Temperature**	100(2) K
**Wavelength**	1.54178 Å
**Crystal size**	0.091 x 0.199 x 0.231 mm
**Crystal system**	monoclinic
**Unit cell dimensions**	a = 5.9970 Å α = 90.000°
	b = 38.7490 Å β = 104.368°
	c = 6.8193 Å. γ = 90.000°
**Volume**	1535.1 $Å^3$
**Z**	2
**Density (calculated)**	1.132 g/cm^3
**Absorption coefficient**	0.657 mm^{-1}
**F(000)**	574

Table S2. Data collection and structure refinement for **Compound 5** ((25R)-Antcin K).

**Theta range for data collection**	4.56 to 66.58°
**Reflections collected**	2758
**Independent reflections**	2758 [R(int) = 0.0785]
**Coverage of independent reflections**	99.80%
**Absorption correction**	Multi-Scan
**Structure solution technique**	direct methods
**Structure solution program**	XT, VERSION 2018/2
**Refinement method**	Full-matrix least-squares on F^2
**Structure solution program**	SHELXS-2018/3 (Sheldrick 2018)
**Function minimized**	Σ w(F_o^2 - F_c^2)^2
**Data / restraints / parameters**	2758 / 263 / 417
**Goodness-of-fit on F^2**	1.671
**Final R indices**	2699 data; I>2σ(I). R1 = 0.1033, wR2 = 0.3119

	all data. R1 = 0.1049, wR2 = 0.3163
**Weighting scheme**	$w=1/[\sigma^2(F_o^2)+(0.2000P)^2]$, where $P=(Fo^2+2Fc^2)/3$
**Largest diff. peak and hole**	1.537 and -1.110 eÅ^{-3}
**R.M.S. deviation from mean**	0.118 eÅ^{-3}

Table S3. Atomic coordinates and equivalent isotropic atomic displacement parameters (Å2) for L178. U(eq) is defined as one third of the trace of the orthogonalized Uij tensor.

x/a	y/b	z/c	U(eq)	
O1	0.9008(6)	0.06395(10)	0.8920(6)	0.0221(8)
O2	0.3715(6)	0.05672(10)	0.0579(6)	0.0223(8)
O3	0.9808(7)	0.95019(10)	0.4568(6)	0.0281(9)
O4	0.2792(6)	0.92375(10)	0.7902(6)	0.0248(8)
C1	0.6982(8)	0.99341(13)	0.1503(7)	0.0183(10)
C2	0.9191(8)	0.01521(13)	0.2274(8)	0.0182(10)
C3	0.8683(8)	0.05384(13)	0.2275(8)	0.0193(10)
C4	0.7452(8)	0.06715(14)	0.0193(8)	0.0196(10)
C5	0.5221(8)	0.04707(13)	0.9298(8)	0.0185(10)
C6	0.5772(9)	0.00750(13)	0.9381(8)	0.0187(11)
C7	0.3696(9)	0.98501(14)	0.8436(8)	0.0207(11)
C8	0.4545(9)	0.94931(14)	0.7983(8)	0.0205(10)
C9	0.6631(8)	0.93704(13)	0.9554(8)	0.0180(10)
C10	0.7654(8)	0.95599(13)	0.1190(8)	0.0179(10)
C11	0.9402(9)	0.93909(14)	0.2837(8)	0.0219(11)
C12	0.0598(9)	0.90732(14)	0.2381(8)	0.0242(11)
C13	0.8856(9)	0.88238(13)	0.1081(8)	0.0218(11)
C14	0.7678(9)	0.90306(14)	0.9159(8)	0.0211(11)
C15	0.6316(11)	0.87531(16)	0.7744(10)	0.0324(13)
C16	0.7982(12)	0.84432(18)	0.8129(11)	0.0403(15)
C17	0.9882(11)	0.85165(16)	0.0167(10)	0.0318(13)
C19	0.5514(9)	0.99404(14)	0.3059(8)	0.0219(11)
C18	0.7047(10)	0.87074(15)	0.2188(9)	0.0280(12)

C20	0.0568(12)	0.81878(16)	0.1406(12)	0.0426(16)
C21	0.2095(12)	0.82524(18)	0.3530(12)	0.0436(17)
C29	0.4126(8)	0.05947(14)	0.7140(8)	0.0210(11)
C22	0.194(4)	0.7944(4)	0.035(3)	0.0656(14)
C23	0.297(3)	0.7631(3)	0.165(2)	0.0664(13)
C24	0.456(3)	0.7408(3)	0.0805(18)	0.0671(12)
C25	0.623(2)	0.7184(3)	0.2304(19)	0.0676(12)
C26	0.510(3)	0.6877(3)	0.310(2)	0.0681(13)
C27	0.784(3)	0.7382(4)	0.398(2)	0.0693(19)
C28	0.459(3)	0.7420(4)	0.881(2)	0.0678(13)
O5	0.456(2)	0.6936(3)	0.4750(18)	0.0692(14)
O6	0.416(2)	0.6661(3)	0.198(2)	0.0665(13)
C22X	0.160(6)	0.7947(4)	0.001(4)	0.0656(14)
C23X	0.138(4)	0.7559(4)	0.040(3)	0.0663(13)
C24X	0.210(3)	0.7322(4)	0.893(2)	0.0674(13)
C25X	0.429(3)	0.7121(4)	0.976(2)	0.0674(12)
C26X	0.402(3)	0.6883(4)	0.147(3)	0.0675(12)
C27X	0.644(3)	0.7337(5)	0.028(3)	0.0683(14)
C28X	0.069(3)	0.7267(5)	0.706(3)	0.070(2)
O5X	0.283(3)	0.6617(3)	0.088(2)	0.0676(16)
O6X	0.479(3)	0.6944(4)	0.319(2)	0.0681(13)
O1S	0.2157(7)	0.61934(11)	0.3817(6)	0.0300(9)
C1S	0.0383(12)	0.63579(18)	0.4525(11)	0.0419(16)
O2S	0.5864(8)	0.61152(11)	0.6899(7)	0.0359(10)
C2S	0.6970(18)	0.6392(3)	0.8150(16)	0.070(3)

b. X-ray crystallographic data for **Compound 10** ((25R)-Antcin A):

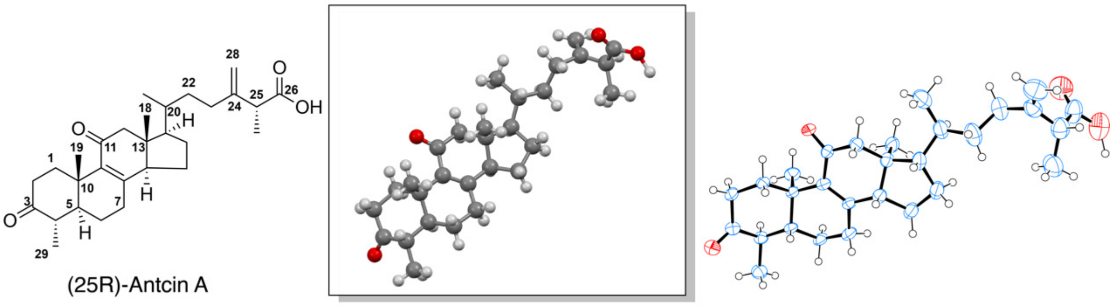

Crystals of **Compound 10** ((25R)-Antcin A): suitable for X-ray analysis were obtained by slow evaporation from MeOH. A colorless Plate-like specimen of $C_{29}H_{42}O_4$, approximate dimensions 0.074 mm x 0.156 mm x 0.314 mm, was used for the X-ray crystallographic analysis. The X-ray intensity data were measured (λ = 1.54178 Å).

The total exposure time was 19.55 hours. The frames were integrated with the Bruker SAINT software package using a narrow-frame algorithm. The integration of the data using a monoclinic unit cell yielded a total of 34101 reflections to a maximum θ angle of 66.73° (0.84 Å resolution), of which 4550 were independent (average redundancy 7.495, completeness = 99.9%, R_{int} = 5.96%, R_{sig} = 3.38%) and 4000 (87.91%) were greater than 2σ(F^2). The final cell constants of a = 9.6573(5) Å, b = 7.6029(4) Å, c = 17.9965(9) Å, β = 100.691(3)°, volume = 1298.43(12) Å^3, are based upon the refinement of the XYZ-centroids of 142 reflections above 20 σ(I) with 5.006° < 2θ < 49.49°. Data were corrected for absorption effects using the Multi-Scan method (SADABS). The ratio of minimum to maximum apparent transmission was 0.818.

The structure was solved and refined using the Bruker SHELXTL Software Package, using the space group P 1 21 1, with Z = 2 for the formula unit, $C_{29}H_{42}O_4$. The final anisotropic full-matrix least-squares refinement on F^2 with 374variables converged at R1 = 6.98%, for the observed data and wR2 = 19.21% for all data. The goodness-of-fit was 1.069. The largest peak in the final difference electron density synthesis was 0.640 e$^-$/Å^3 and the largest hole was -0.436 e$^-$/Å^3 with an RMS deviation of 0.057 e$^-$/Å^3. On the basis of the final model, the calculated density was 1.163g/cm^3 and F(000), 496 e$^-$.

Crystallographic data have been deposited with the Cambridge Crystallograhic Data Centre (CCDC# 2099280). Copies of the data can be obtained free of charge on application to the CCDC, 12 Union Road, Cambridge CB21EZ, UK (fax: (+44)-1223-336-033; e-mail: deposit@ccdc.cam.ac.uk).

Table S4. Sample and crystal data for **Compound 10** ((25R)-Antcin A).

**Identification code**	L250

**Chemical formula**	$C_{29}H_{42}O_4$
**Formula weight**	454.62 g/mol
**Temperature**	100(2) K
**Wavelength**	1.54178 Å
**Crystal size**	0.074 x 0.156 x 0.314 mm
**Crystal system**	monoclinic
**Unit cell dimensions**	a = 9.6573(5) Å α = 90.000°
	b = 7.6029(4) Å β = 100.691(3)°
	c = 17.9965(9) Å γ = 90.000°
**Volume**	1298.43(12) $Å^3$
**Z**	2
**Density (calculated)**	1.163 g/cm^3
**Absorption coefficient**	0.593 mm^{-1}
**F(000)**	496

Table S5. Data collection and structure refinement for **Compound 10** ((25R)-Antcin A).

**Theta range for data collection**	2.50 to 66.73°
**Reflections collected**	34101
**Independent reflections**	4550 [R(int) = 0.0596]
**Coverage of independent reflections**	99.9%
**Absorption correction**	Multi-Scan
**Structure solution technique**	direct methods
**Structure solution program**	XT, VERSION 2018/2
**Refinement method**	Full-matrix least-squares on F^2
**Structure solution program**	SHELXS-2018/3 (Sheldrick 2018)
**Function minimized**	Σ w(F_o^2 - F_c^2)^2
**Data / restraints / parameters**	4550 / 239 / 374
**Goodness-of-fit on F^2**	1.069
**Final R indices**	4000 data; I>2σ(I). R1 = 0.0698, wR2 = 0.1803
	all data. R1 = 0.0800, wR2 = 0.1921

**Weighting scheme**	w=1/[$\sigma^2(F_o^2)$+(0.0972P)^2+1.1378P], where P=(Fo^2+2Fc^2)/3
**Largest diff. peak and hole**	0.640 and -0.436 eÅ^{-3}
**R.M.S. deviation from mean**	0.057 eÅ^{-3}

Table S6. Atomic coordinates and equivalent isotropic atomic displacement parameters (Å2) for L178.

U(eq) is defined as one third of the trace of the orthogonalized Uij tensor.

	x/a	y/b	z/c	U(eq)
O1	0.0638(3)	0.5572(4)	0.8264(2)	0.0401(8)
O2	0.3795(4)	0.2031(5)	0.1345(2)	0.0454(9)
C1	0.1614(5)	0.5502(6)	0.8803(3)	0.0333(10)
C2	0.2175(5)	0.7111(6)	0.9266(3)	0.0330(10)
C3	0.2031(5)	0.6741(5)	0.0091(3)	0.0281(9)
C4	0.2387(5)	0.8320(6)	0.0610(3)	0.0365(11)
C5	0.1993(5)	0.7961(6)	0.1371(3)	0.0386(11)
C6	0.2235(4)	0.6110(6)	0.1656(3)	0.0300(10)
C7	0.1918(5)	0.5759(6)	0.2430(3)	0.0367(11)
C8	0.1955(6)	0.7243(8)	0.3004(3)	0.0466(13)
C9	0.1965(6)	0.6250(9)	0.3752(3)	0.0515(14)
C10	0.2174(6)	0.4265(8)	0.3600(3)	0.0455(13)
C11	0.2819(5)	0.4304(6)	0.2875(3)	0.0378(11)
C12	0.2598(6)	0.2684(6)	0.2355(3)	0.0400(12)
C13	0.3065(5)	0.3102(6)	0.1611(3)	0.0353(11)
C14	0.2657(4)	0.4812(6)	0.1235(3)	0.0303(10)
C15	0.2881(4)	0.5094(6)	0.0424(3)	0.0295(10)
C16	0.2288(5)	0.3518(6)	0.9923(3)	0.0332(10)
C17	0.2304(6)	0.3798(6)	0.9081(3)	0.0387(11)
C18	0.1447(6)	0.8783(7)	0.8925(3)	0.0454(13)
C19	0.4385(5)	0.4768(8)	0.3050(3)	0.0450(13)
C20	0.4477(4)	0.5314(7)	0.0439(3)	0.0403(12)
C21	0.2981(7)	0.3266(9)	0.4303(3)	0.0543(15)
C22	0.3230(10)	0.1322(10)	0.4140(4)	0.077(2)
C23	0.2195(7)	0.3374(12)	0.4967(3)	0.0666(19)

C24	0.3096(8)	0.2919(11)	0.5725(4)	0.0679(18)
O3	0.4879(7)	0.4020(14)	0.7242(5)	0.088(2)
O4	0.3744(11)	0.6047(14)	0.7778(5)	0.082(3)
C25	0.2425(8)	0.2798(12)	0.6410(4)	0.0549(19)
C26	0.1844(11)	0.1218(15)	0.6556(7)	0.076(3)
C27	0.2325(10)	0.4318(14)	0.6860(5)	0.065(2)
C28	0.1645(14)	0.5966(15)	0.6443(6)	0.081(3)
C29	0.3805(11)	0.4768(16)	0.7322(7)	0.069(2)
O3A	0.476(3)	0.320(4)	0.7624(17)	0.096(4)
O4A	0.393(4)	0.538(4)	0.7954(18)	0.082(4)
C25A	0.215(3)	0.384(4)	0.6249(12)	0.074(4)
C26A	0.092(3)	0.510(4)	0.6228(17)	0.071(5)
C27A	0.235(3)	0.336(3)	0.7080(13)	0.065(3)
C28A	0.239(3)	0.139(3)	0.7154(15)	0.064(4)
C29A	0.379(3)	0.425(4)	0.742(2)	0.074(4)

c. X-ray crystallographic data for **Compound 11** (Versisponic acid D):

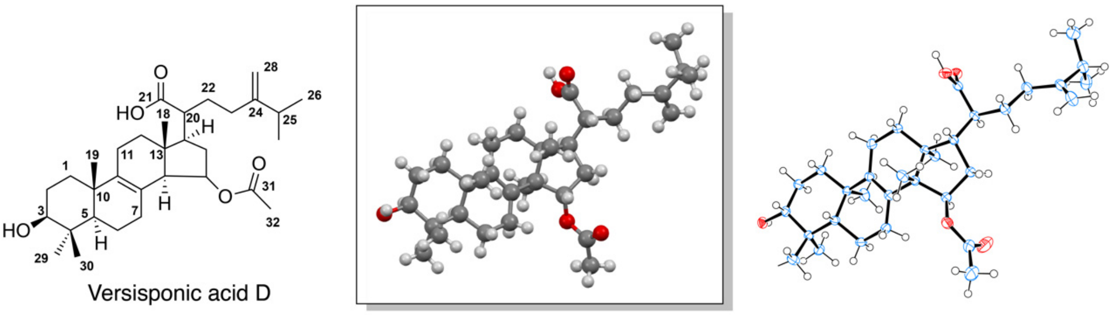

Crystals of **Compound 11** (Versisponic acid D): suitable for X-ray analysis were obtained by slow evaporation from MeOH. A specimen of $C_{33}H_{52}O_{5.12}$, approximate dimensions 0.017 mm x 0.034 mm x 0.085 mm, was used for the X-ray crystallographic analysis. The X-ray intensity data were measured (λ = 1.54178 Å).

The total exposure time was 16.91 hours. The frames were integrated with the Bruker SAINT software package using a narrow-frame algorithm. The integration of the data using an orthorhombic unit cell yielded a total of 69462reflections to a maximum θ angle of 70.33° (0.82 Å resolution), of

which 5631 were independent (average redundancy 12.336, completeness = 99.5%, R_{int} = 6.70%, R_{sig} = 2.43%) and 5171 (91.83%) were greater than 2σ(F^2).The final cell constants of a = 6.0024(2) Å, b = 21.8413(8) Å, c = 22.5731(9) Å, volume = 2959.34(19) $Å^3$, are based upon the refinement of the XYZ-centroids of 9844 reflections above 20 σ(I) with 5.630° < 2θ < 140.1°. Data were corrected for absorption effects using the Multi-Scan method (SADABS). The ratio of minimum to maximum apparent transmission was 0.902. The calculated minimum and maximum transmission coefficients (based on crystal size) are 0.6658 and 0.7480.

The structure was solved and refined using the Bruker SHELXTL Software Package, using the space group P 21 21 21, with Z = 4 for the formula unit, $C_{33}H_{52}O_{5.12}$. The final anisotropic full-matrix least-squares refinement on F^2 with 397 variables converged at R1 = 4.17%, for the observed data and wR2 = 11.62% for all data. The goodness-of-fit was 1.043. The largest peak in the final difference electron density synthesis was 0.325 $e^-/Å^3$ and the largest hole was -0.272 $e^-/Å^3$ with an RMS deviation of 0.045 $e^-/Å^3$. On the basis of the final model, the calculated density was 1.191g/cm^3 and F(000), 1164 e^-.

Crystallographic data have been deposited with the Cambridge Crystallograhic Data Centre (CCDC# 2099279). Copies of the data can be obtained free of charge on application to the CCDC, 12 Union Road, Cambridge CB21EZ, UK (fax: (+44)-1223-336-033; e-mail: deposit@ccdc.cam.ac.uk).

Table S7. Sample and crystal data for **Compound 11** (Versisponic acid D).

**Identification code**	L242
**Chemical formula**	$C_{33}H_{52}O_{5.12}$
**Formula weight**	530.74 g/mol
**Temperature**	100(2) K
**Wavelength**	1.54178 Å
**Crystal size**	0.017 x 0.034 x 0.085 mm
**Crystal system**	orthorhombic
**Unit cell dimensions**	a = 6.0024(2) Å α = 90.000°
	b = 21.8413(8) Å β = 90.000°
	c = 22.5731(9) Å. γ = 90.000°
**Volume**	2959.34(19) $Å^3$
**Z**	4

**Density (calculated)**	1.191 g/cm^3
**Absorption coefficient**	0.616 mm^{-1}
**F(000)**	1164

Table S8. Data collection and structure refinement for **Compound 11** (Versisponic acid D).

**Theta range for data collection**	2.81 to 70.33°
**Reflections collected**	69462
**Independent reflections**	5631 [R(int) = 0.0670]
**Coverage of independent reflections**	99.5%
**Absorption correction**	Multi-Scan
**Structure solution technique**	direct methods
**Structure solution program**	XT, VERSION 2018/2
**Refinement method**	Full-matrix least-squares on F^2
**Structure solution program**	SHELXS-2018/3 (Sheldrick 2018)
**Function minimized**	Σ w(F_o^2 - F_c^2)^2
**Data / restraints / parameters**	5631 / 46 / 397
**Goodness-of-fit on F^2**	1.043
**Final R indices**	5171 data; I>2σ(I). R1 = 0.0417, wR2 = 0.1100
	all data. R1 = 0.0473, wR2 = 0.1162
**Weighting scheme**	w=1/[$\sigma^2(F_o^2)+(0.0674P)^2+1.0383P$], where P=($Fo^2+2Fc^2$)/3
**Largest diff. peak and hole**	0.325 and -0.272 eÅ^{-3}
**R.M.S. deviation from mean**	0.045 eÅ^{-3}

Table S9. Atomic coordinates and equivalent isotropic atomic displacement parameters (Å2) for L178. U(eq) is defined as one third of the trace of the orthogonalized Uij tensor.

	**x/a**	**y/b**	**z/c**	**U(eq)**
O1	0.6283(3)	0.53458(8)	0.88938(8)	0.0244(4)
O2	0.4970(4)	0.79367(9)	0.22479(8)	0.0385(6)
O4	0.4278(3)	0.51593(8)	0.38584(9)	0.0271(4)
O5	0.1106(3)	0.56659(9)	0.36887(8)	0.0256(4)

C1	0.6665(4)	0.61743(11)	0.96450(11)	0.0198(5)
C2	0.5625(4)	0.55476(11)	0.94786(10)	0.0200(5)
C3	0.6064(5)	0.50452(11)	0.99278(11)	0.0242(6)
C4	0.5146(5)	0.52323(11)	0.05299(11)	0.0230(5)
C5	0.6222(4)	0.58198(11)	0.07667(10)	0.0182(5)
C6	0.5100(4)	0.60297(11)	0.13432(11)	0.0188(5)
C7	0.4122(5)	0.55612(13)	0.17252(11)	0.0262(6)
C8	0.3274(4)	0.57448(11)	0.23378(10)	0.0199(5)
C9	0.4594(4)	0.62903(11)	0.25703(10)	0.0166(5)
C10	0.3810(4)	0.65993(11)	0.31530(10)	0.0185(5)
C11	0.4911(5)	0.72440(11)	0.31071(11)	0.0231(5)
C12	0.5586(5)	0.73330(11)	0.24526(11)	0.0236(6)
C13	0.4348(4)	0.68281(11)	0.21158(11)	0.0183(5)
C14	0.5269(4)	0.66377(12)	0.15156(11)	0.0197(5)
C15	0.6121(5)	0.71128(11)	0.11259(11)	0.0241(5)
C16	0.7013(5)	0.69276(12)	0.05251(12)	0.0264(6)
C17	0.5959(4)	0.63325(11)	0.02962(10)	0.0190(5)
C18	0.5670(5)	0.66559(13)	0.92248(11)	0.0286(6)
C19	0.9201(4)	0.61645(13)	0.95497(12)	0.0258(6)
C20	0.8663(5)	0.56819(14)	0.09373(12)	0.0286(6)
C21	0.7053(4)	0.60902(12)	0.26376(11)	0.0219(5)
C22	0.1916(5)	0.70331(13)	0.20206(11)	0.0257(6)
O3	0.8505(15)	0.8251(3)	0.2592(3)	0.0538(17)
C23	0.6690(15)	0.8359(3)	0.2352(3)	0.0335(17)
C24	0.599(2)	0.8986(3)	0.2164(3)	0.055(2)
O3A	0.763(3)	0.8368(6)	0.2673(7)	0.059(3)
C23A	0.585(3)	0.8457(5)	0.2407(6)	0.045(3)
C24A	0.477(3)	0.9014(5)	0.2225(5)	0.044(3)
C25	0.4396(4)	0.62632(11)	0.37345(10)	0.0198(5)
C26	0.3301(4)	0.56385(12)	0.37641(10)	0.0206(5)
C27	0.3703(5)	0.66397(12)	0.42838(11)	0.0232(5)
C28	0.4503(5)	0.63565(13)	0.48616(11)	0.0279(6)

C29	0.4285(5)	0.67512(13)	0.54110(11)	0.0268(6)
C30	0.3380(5)	0.73066(14)	0.54068(13)	0.0322(6)
C31	0.5220(5)	0.64953(13)	0.59841(12)	0.0314(6)
C32	0.7729(5)	0.63935(15)	0.59498(14)	0.0362(7)
C33	0.4025(7)	0.59103(15)	0.61718(14)	0.0430(8)
O1W	0.088(5)	0.9409(9)	0.2386(7)	0.060(7)

d. X-ray crystallographic data for **Compound 22** ((25R)-Antcin C):

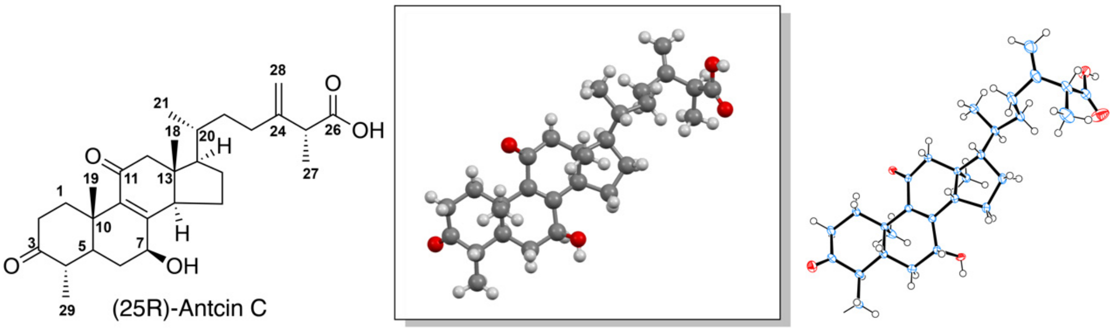

Crystals of **Compound 22** ((25R)-Antcin C): suitable for X-ray analysis were obtained by slow evaporation from MeOH. A specimen of $C_{29}H_{42}O_5$, approximate dimensions 0.261 mm × 0.565 mm × 0.574 mm, was used for the X-ray crystallographic analysis. The X-ray intensity data were measured (λ = 1.54184 Å).

The total exposure time was 15.63 hours. The frames were integrated with the Bruker SAINT software package using a narrow-frame algorithm. The integration of the data using a monoclinic unit cell yielded a total of 24220 reflections to a maximum θ angle of 66.58° (0.84 Å resolution), of which 4512 were independent (average redundancy 5.368, completeness = 99.9%, R_{int} = 3.41%, R_{sig} = 2.18%) and 4472 (99.11%) were greater than $2\sigma(F^2)$. The final cell constants of a = 9.5620 Å, b = 7.8918 Å, c = 17.0478 Å, α = 90.000°, β = 98.086°, γ = 90.000°, volume = 1273.7 Å^3, are based upon the refinement of the XYZ-centroids of 36 reflections above 20 σ(I) with 12.38° < 2θ < 53.15°. Data were corrected for absorption effects using the Multi-Scan method (SADABS). The ratio of minimum to maximum apparent transmission was 0.865.

The structure was solved and refined using the Bruker SHELXTL Software Package, with Z = 2 for the formula unit, $C_{29}H_{42}O_5$. The final anisotropic full-matrix least-squares refinement on F^2 with 320 variables converged at R1 = 2.84%, for the observed data and wR2 = 7.20% for all data. The goodness-of-fit was 1.033. The largest peak in the final difference electron density synthesis was 0.168

$e^-/Å^3$ and the largest hole was -0.145 $e^-/Å^3$ with an RMS deviation of 0.033 $e^-/Å^3$. On the basis of the final model, the calculated density was 1.223 g/cm^3 and F(000), 512 e^-.

Crystallographic data have been deposited with the Cambridge Crystallograhic Data Centre (CCDC# 2099281). Copies of the data can be obtained free of charge on application to the CCDC, 12 Union Road, Cambridge CB21EZ, UK (fax: (+44)-1223-336-033; e-mail: deposit@ccdc.cam.ac.uk).

Table S10. Sample and crystal data for **Compound 22** ((25R)-Antcin C).

**Identification code**	L174
**Chemical formula**	$C_{29}H_{42}O_5$
**Formula weight**	470.62 g/mol
**Temperature**	100(2) K
**Wavelength**	1.54184 Å
**Crystal size**	0.261 x 0.565 x 0.574 mm
**Crystal system**	monoclinic
**Unit cell dimensions**	a = 9.5620 Å α = 90.000°
	b = 7.8918 Å β = 98.086°
	c = 17.0478 Å. γ = 90.000°
**Volume**	1273.7 $Å^3$
**Z**	2
**Density (calculated)**	1.223 g/cm^3
**Absorption coefficient**	0.650 mm^{-1}
**F(000)**	512

Table S11. Data collection and structure refinement for **Compound 22** ((25R)-Antcin C).

**Theta range for data collection**	2.62 to 66.58°
**Reflections collected**	24220
**Independent reflections**	4512 [R(int) = 0.0341]
**Coverage of independent reflections**	99.9%
**Absorption correction**	Multi-Scan
**Structure solution technique**	direct methods

**Structure solution program**	XT, VERSION 2018/2
**Refinement method**	Full-matrix least-squares on F^2
**Structure solution program**	SHELXS-2018/3 (Sheldrick 2018)
**Function minimized**	Σ w(F_o^2 - F_c^2)^2
**Data / restraints / parameters**	4512 / 1 / 320
**Goodness-of-fit on F^2**	1.033
**Final R indices**	4472 data; I>2σ(I). R1 = 0.0284, wR2 = 0.0716
	all data. R1 = 0.0294, wR2 = 0.0720
**Weighting scheme**	w=1/[σ^2(F_o^2)+(0.0388P)^2+0.2886P], where P=(Fo^2+2Fc^2)/3
**Largest diff. peak and hole**	0.168 and -0.145 eÅ^{-3}
**R.M.S. deviation from mean**	0.033 eÅ^{-3}

Table S12. Atomic coordinates and equivalent isotropic atomic displacement parameters (Å2) for L178. U(eq) is defined as one third of the trace of the orthogonalized Uij tensor.

	x/a	y/b	z/c	U(eq)
O1	0.98061(15)	0.5820(2)	0.16782(8)	0.0292(3)
O2	0.61943(15)	0.89870(19)	0.80886(8)	0.0252(3)
O3	0.63842(14)	0.21618(17)	0.87157(7)	0.0207(3)
O4	0.63809(16)	0.31064(19)	0.25861(9)	0.0291(3)
O5	0.6602(2)	0.5868(2)	0.28017(13)	0.0503(5)
C1	0.71413(18)	0.5230(2)	0.96091(10)	0.0160(3)
C2	0.7888(2)	0.3737(2)	0.00853(11)	0.0235(4)
C3	0.8054(3)	0.4003(3)	0.09894(11)	0.0304(5)
C4	0.8723(2)	0.5691(3)	0.12185(10)	0.0208(4)
C5	0.79811(19)	0.7191(2)	0.07951(11)	0.0186(4)
C6	0.79680(18)	0.6867(2)	0.98897(10)	0.0160(4)
C7	0.7416(2)	0.8391(2)	0.93966(11)	0.0232(4)
C8	0.74004(18)	0.8143(2)	0.85072(10)	0.0159(3)
C9	0.73374(18)	0.6324(2)	0.82246(10)	0.0150(4)
C10	0.71978(17)	0.5008(2)	0.87183(10)	0.0146(3)
C11	0.69334(19)	0.3285(2)	0.83609(10)	0.0164(4)

C12	0.7283(2)	0.2957(2)	0.75401(10)	0.0182(4)
C13	0.67560(18)	0.4445(2)	0.69987(10)	0.0160(4)
C14	0.75323(19)	0.6018(2)	0.73692(10)	0.0167(4)
C15	0.7229(2)	0.7385(2)	0.67263(11)	0.0207(4)
C16	0.7224(2)	0.6365(2)	0.59478(11)	0.0199(4)
C17	0.72222(18)	0.4447(2)	0.61636(10)	0.0173(4)
C18	0.63596(19)	0.3347(2)	0.55248(10)	0.0191(4)
C19	0.6844(2)	0.3533(3)	0.47032(10)	0.0220(4)
C20	0.8343(2)	0.2876(3)	0.46616(11)	0.0298(5)
C21	0.8623(2)	0.2559(3)	0.38208(12)	0.0288(5)
C22	0.8571(2)	0.4059(3)	0.32591(11)	0.0269(4)
C23	0.7081(2)	0.4480(3)	0.28712(11)	0.0240(4)
C25	0.5573(2)	0.5322(3)	0.97226(12)	0.0275(4)
C26	0.8632(2)	0.8868(3)	0.11054(11)	0.0263(4)
C27	0.51453(18)	0.4618(2)	0.69464(10)	0.0186(4)
C28	0.6331(2)	0.1469(3)	0.57611(11)	0.0230(4)
C29	0.8871(2)	0.1017(3)	0.35739(13)	0.0369(5)
C30	0.9319(3)	0.5654(4)	0.36268(14)	0.0391(6)

Copies NMR SPECTRA

A ^1H NMR, DMSO-d6, 500 MHz

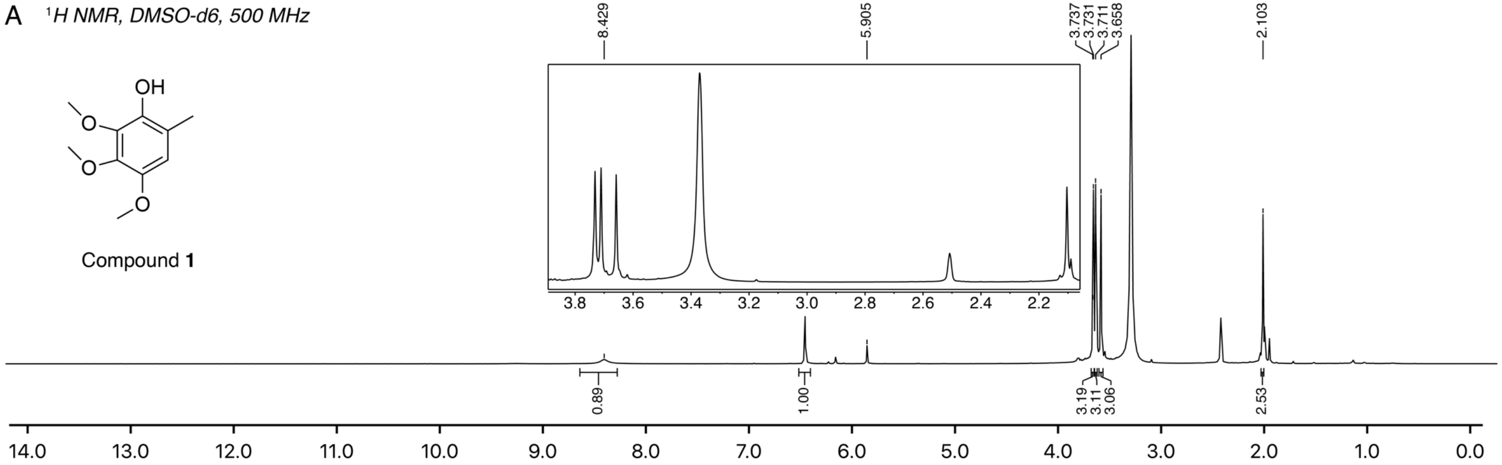

B ^{13}C NMR, DMSO-d6, 126 MHz

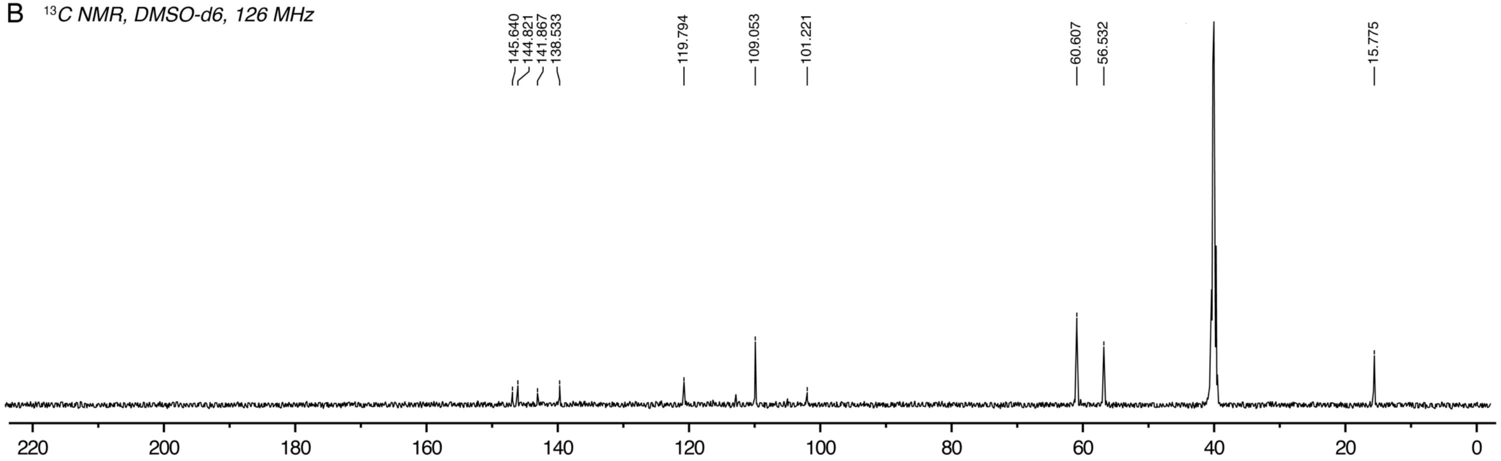

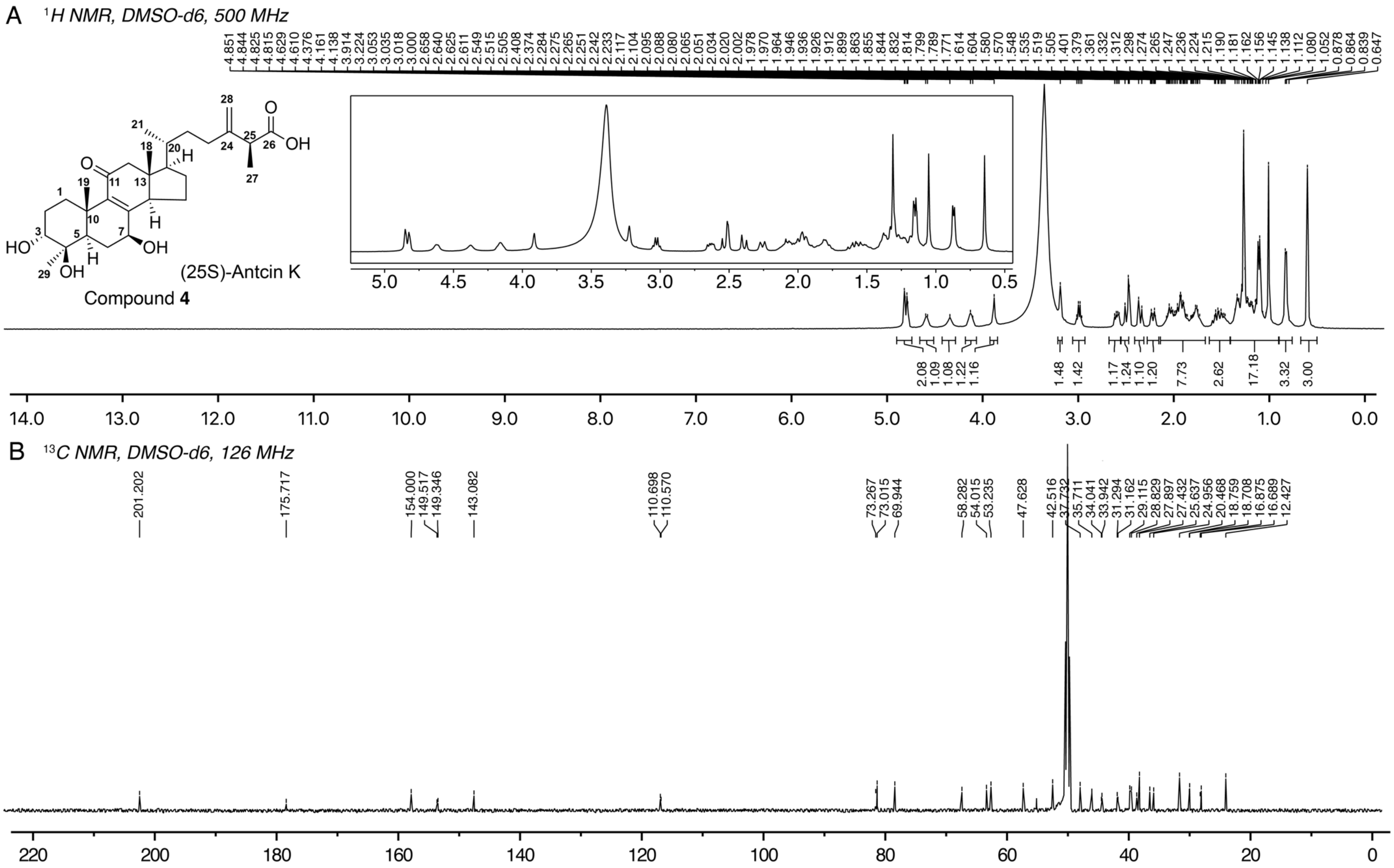

A
1H NMR, DMSO-d6, 500 MHz
4.851 4.844 4.825 4.815 4.629 4.610 4.376 4.161 4.138 3.914 3.224 3.053 3.035 3.018 3.000 2.658 2.640 2.625 2.611 2.549 2.515 2.505 2.408 2.374 2.284 2.275 2.265 2.251 2.242 2.233 2.117 2.104 2.095 2.088 2.080 2.065 2.051 2.034 2.020 2.002 1.978 1.970 1.964 1.946 1.936 1.926 1.912 1.899 1.863 1.855 1.844 1.832 1.814 1.799 1.789 1.771 1.614 1.604 1.580 1.570 1.548 1.535 1.519 1.505 1.401 1.379 1.361 1.332 1.312 1.298 1.274 1.265 1.247 1.236 1.224 1.215 1.190 1.181 1.162 1.156 1.145 1.138 1.112 1.080 1.052 0.878 0.864 0.839 0.647
(25S)-Antcin K
Compound 4
2.08 1.09 1.08 1.22 1.16 1.48 1.42 1.17 1.24 1.10 1.20 7.73 2.62 17.18 3.32 3.00
14.0 13.0 12.0 11.0 10.0 9.0 8.0 7.0 6.0 5.0 4.0 3.0 2.0 1.0 0.0
B
13C NMR, DMSO-d6, 126 MHz
201.202 175.717 154.000 149.517 149.346 143.082 110.698 110.570 73.267 73.015 69.944 58.282 54.015 53.235 47.628 42.516 37.732 35.711 34.041 33.942 31.294 31.162 29.115 28.829 27.897 27.432 25.637 24.956 20.468 18.759 18.708 16.875 16.689 12.427
220 200 180 160 140 120 100 80 60 40 20 0

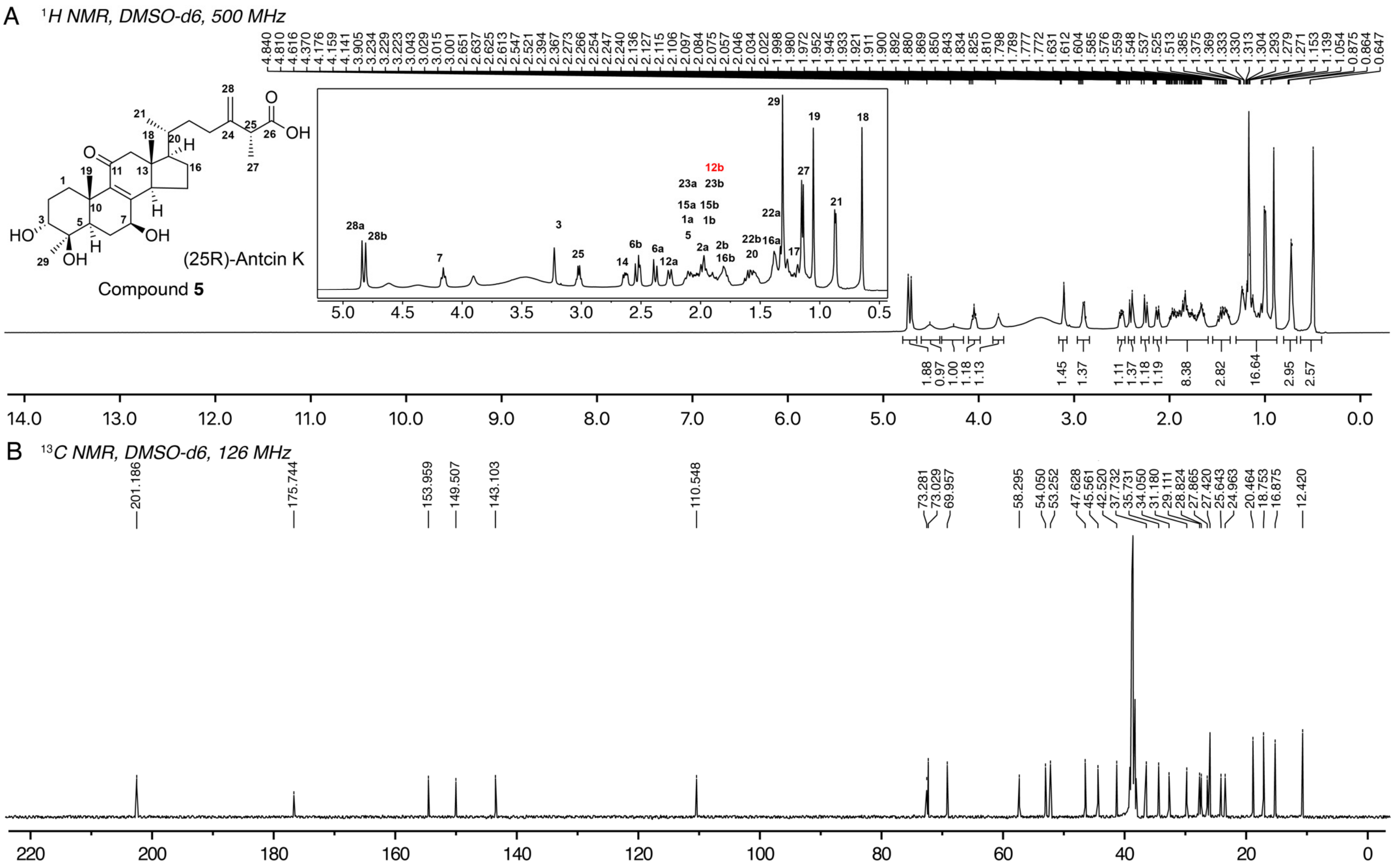

A
¹H NMR, DMSO-d6, 500 MHz
4.840 4.810 4.616 4.370 4.176 4.159 4.141 3.905 3.234 3.229 3.223 3.043 3.029 3.015 3.001 2.651 2.637 2.625 2.613 2.547 2.521 2.394 2.367 2.273 2.266 2.254 2.247 2.240 2.136 2.127 2.115 2.106 2.097 2.084 2.075 2.057 2.046 2.034 2.022 1.998 1.980 1.972 1.952 1.945 1.933 1.921 1.911 1.900 1.892 1.880 1.869 1.850 1.843 1.834 1.825 1.810 1.798 1.789 1.777 1.772 1.631 1.612 1.604 1.585 1.576 1.559 1.548 1.537 1.525 1.513 1.385 1.375 1.369 1.333 1.330 1.313 1.304 1.293 1.279 1.271 1.153 1.139 1.054 0.875 0.864 0.647
(25R)-Antcin K
Compound 5
28a 28b 7 3 25 14 6b 6a 12a 23a 15a 1a 5 12b 23b 15b 1b 2a 2b 16b 22b 20 16a 22a 29 17 27 19 21 18
5.0 4.5 4.0 3.5 3.0 2.5 2.0 1.5 1.0 0.5
1.88 0.97 1.00 1.18 1.13 1.45 1.37 1.11 1.37 1.18 1.19 8.38 2.82 16.64 2.95 2.57
14.0 13.0 12.0 11.0 10.0 9.0 8.0 7.0 6.0 5.0 4.0 3.0 2.0 1.0 0.0
B
¹³C NMR, DMSO-d6, 126 MHz
201.186 175.744 153.959 149.507 143.103 110.548 73.281 73.029 69.957 58.295 54.050 53.252 47.628 45.561 42.520 37.732 35.731 34.050 31.180 29.111 28.824 27.865 27.420 25.643 24.963 20.464 18.753 16.875 12.420
220 200 180 160 140 120 100 80 60 40 20 0

A ^{1}H NMR, DMSO-d6, 500 MHz

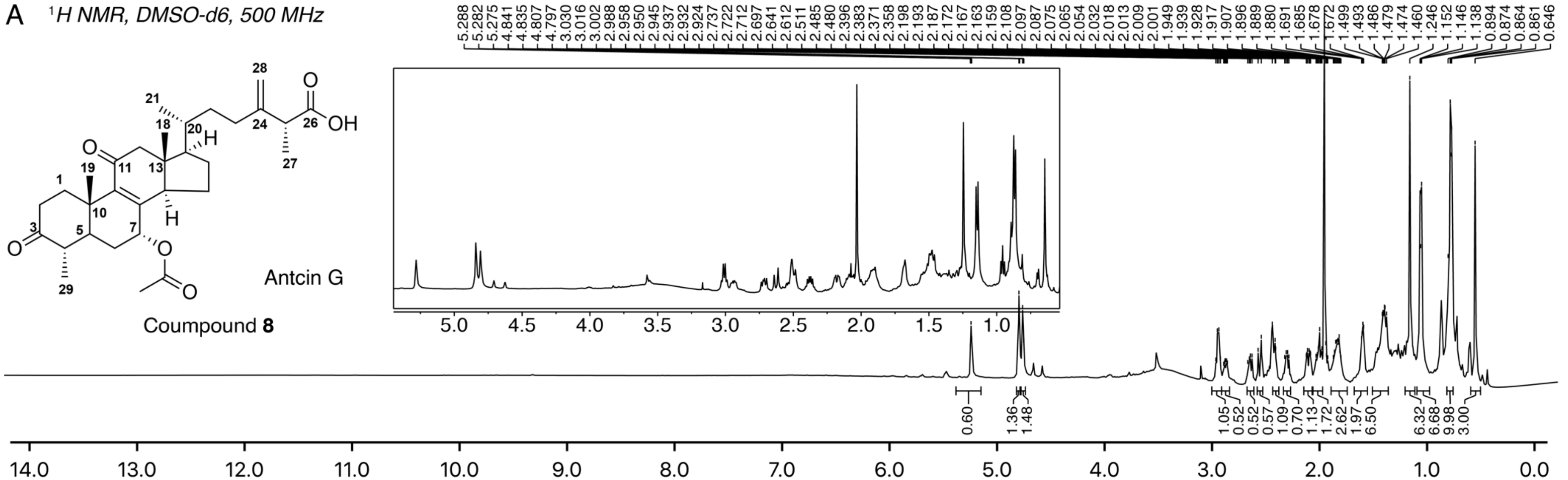

B ^{13}C NMR, DMSO-d6, 126 MHz

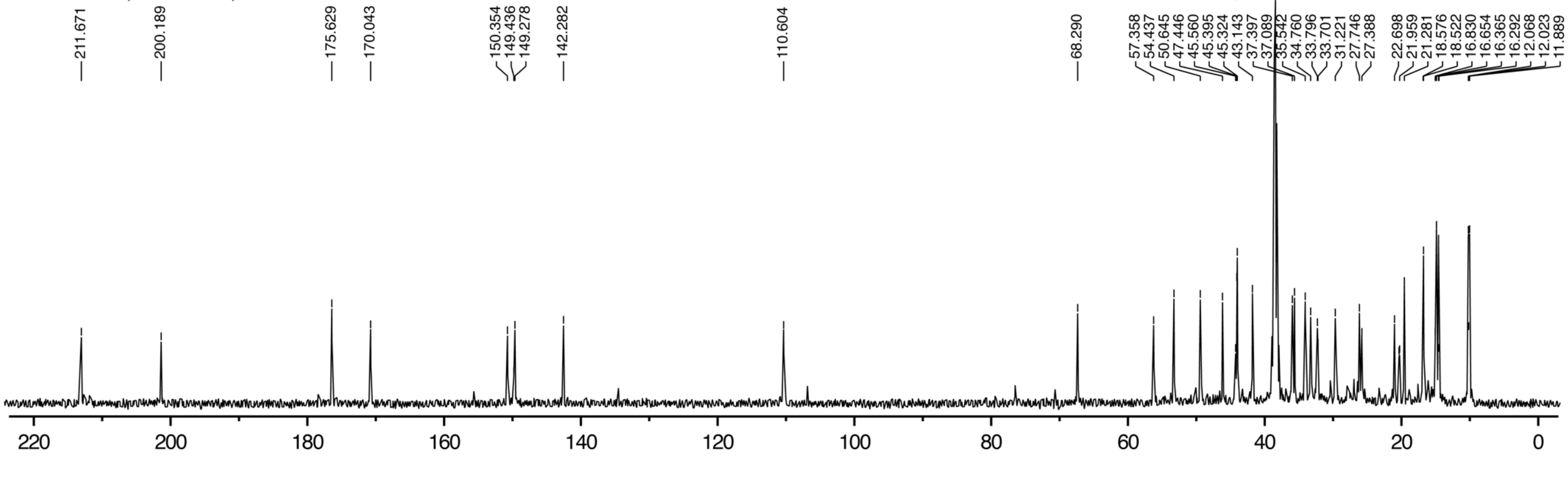

A ^{1}H NMR, DMSO-d6, 500 MHz

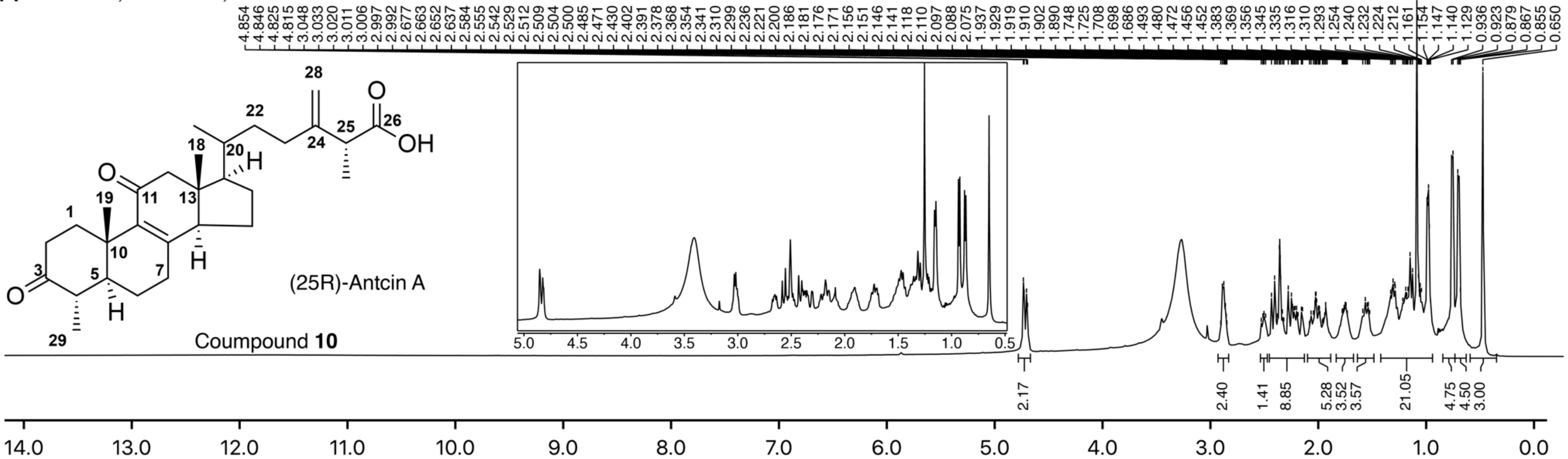

B ^{13}C NMR, DMSO-d6, 126 MHz

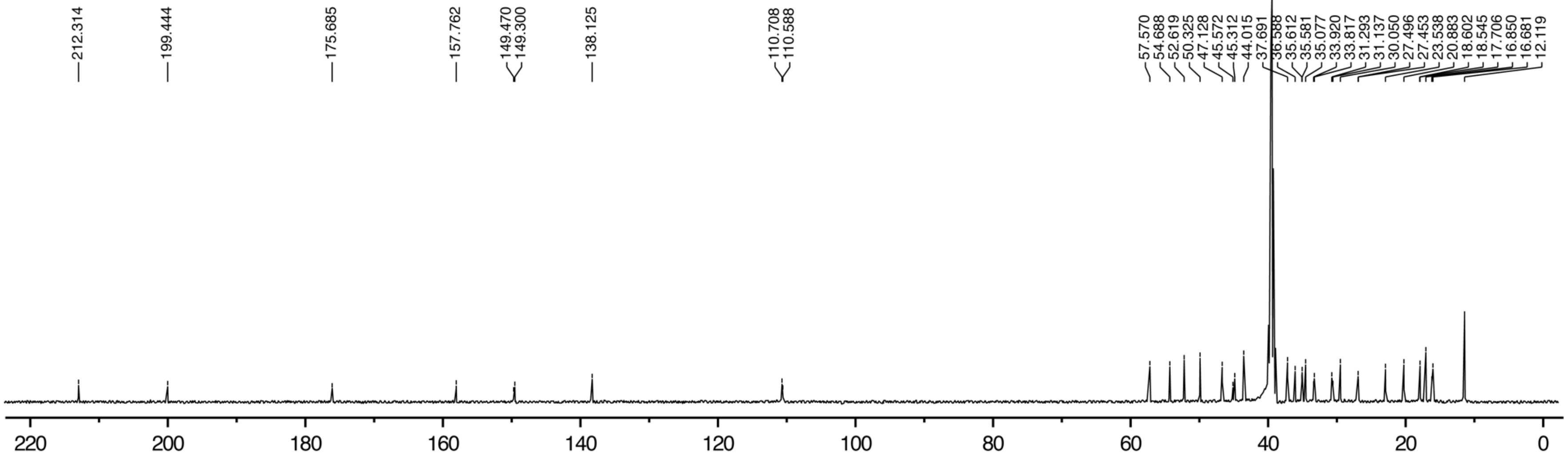

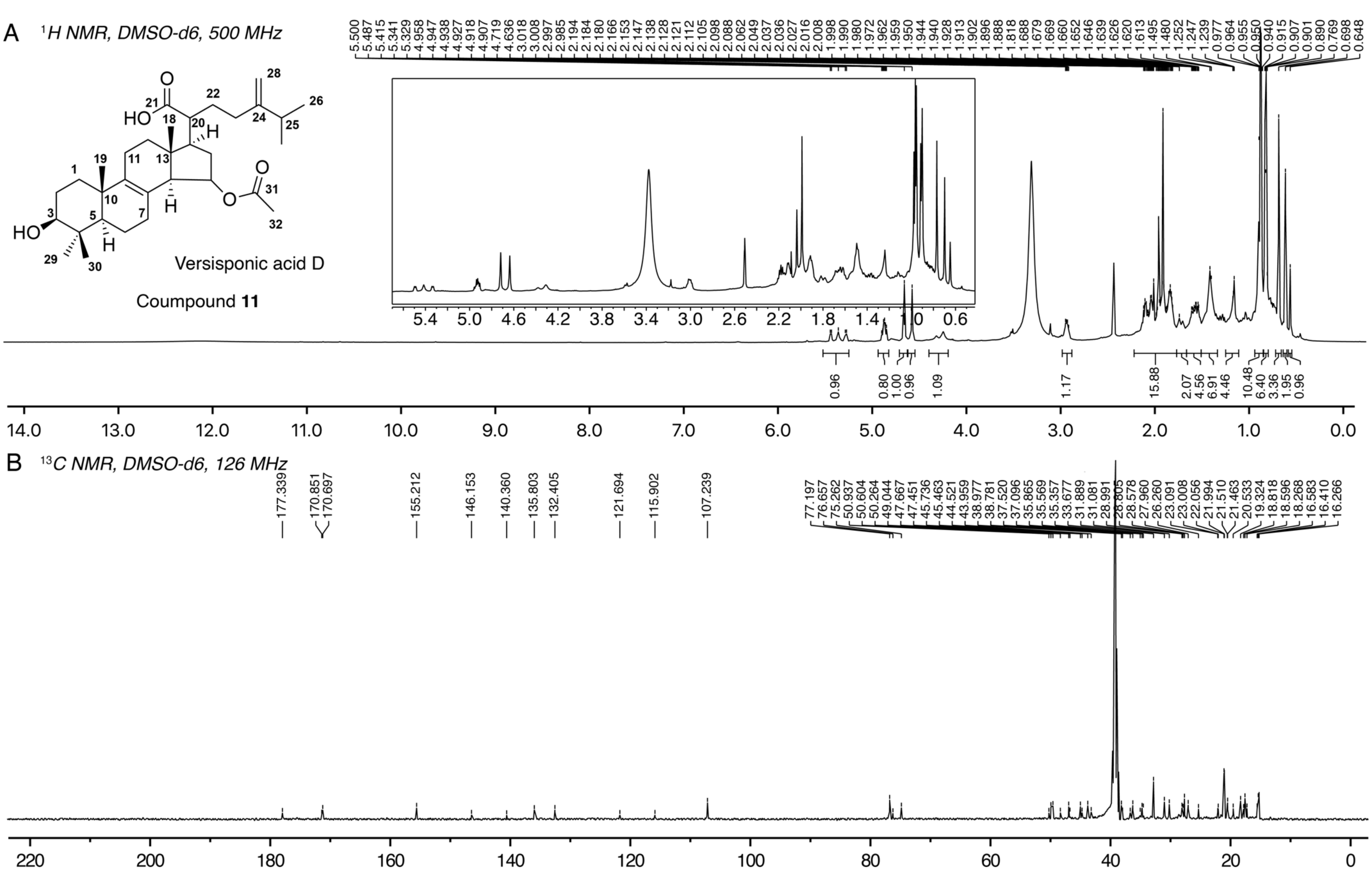
A
1H NMR, DMSO-d6, 500 MHz
5.500 5.487 5.415 5.341 5.329 4.958 4.947 4.938 4.927 4.918 4.907 4.719 4.636 3.018 3.008 2.997 2.985 2.194 2.184 2.180 2.166 2.153 2.147 2.138 2.128 2.121 2.112 2.105 2.098 2.088 2.062 2.049 2.037 2.036 2.027 2.016 2.008 2.008 1.998 1.990 1.980 1.972 1.962 1.959 1.950 1.944 1.940 1.928 1.913 1.902 1.896 1.888 1.818 1.688 1.679 1.669 1.660 1.652 1.646 1.639 1.626 1.620 1.613 1.495 1.480 1.252 1.247 1.239 0.977 0.964 0.955 0.950 0.940 0.915 0.907 0.901 0.890 0.769 0.698 0.648
Versisponic acid D
Coumpound 11
0.96 0.80 1.00 0.96 1.09 1.17 15.88 2.07 4.56 6.91 4.46 10.48 6.40 3.36 1.95 0.96
B
13C NMR, DMSO-d6, 126 MHz
177.339 170.851 170.697 155.212 146.153 140.360 135.803 132.405 121.694 115.902 107.239
77.197 76.657 75.262 50.937 50.604 50.264 49.044 47.667 47.451 45.736 45.463 44.521 43.959 38.977 38.781 37.520 37.096 35.865 35.569 35.357 33.677 31.889 31.081 28.991 28.805 28.578 27.960 26.260 23.091 23.008 22.056 21.994 21.510 21.463 20.533 19.324 18.818 18.596 18.268 16.583 16.410 16.266

A ^{1}H NMR, DMSO-d6, 500 MHz

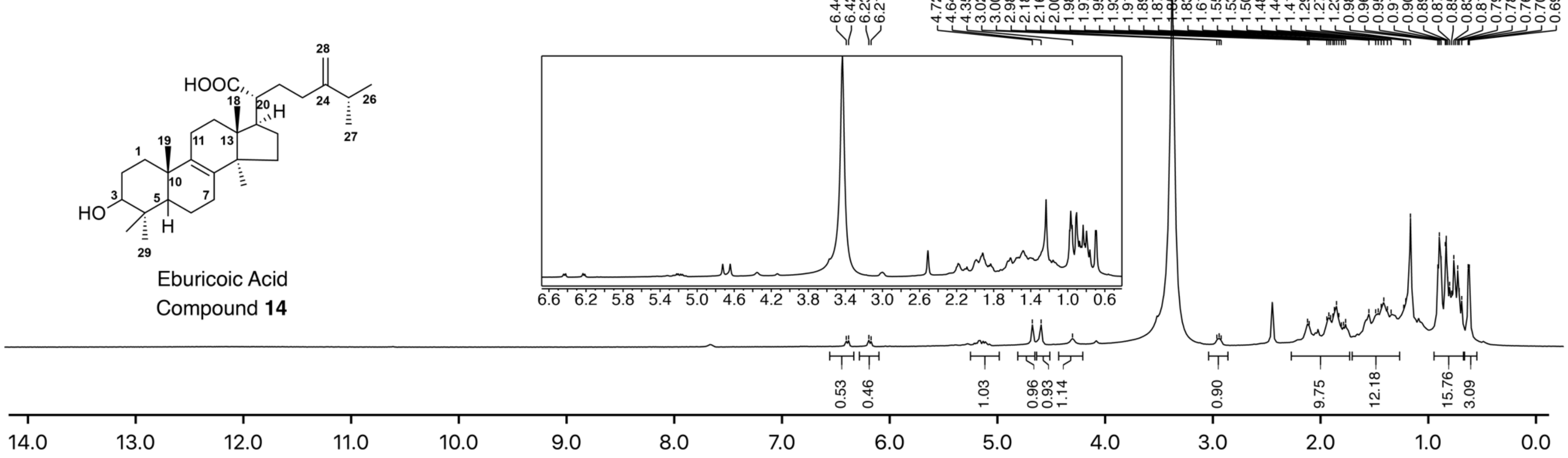

Eburicoic Acid
Compound **14**

B ^{13}C NMR, DMSO-d6, 126 MHz

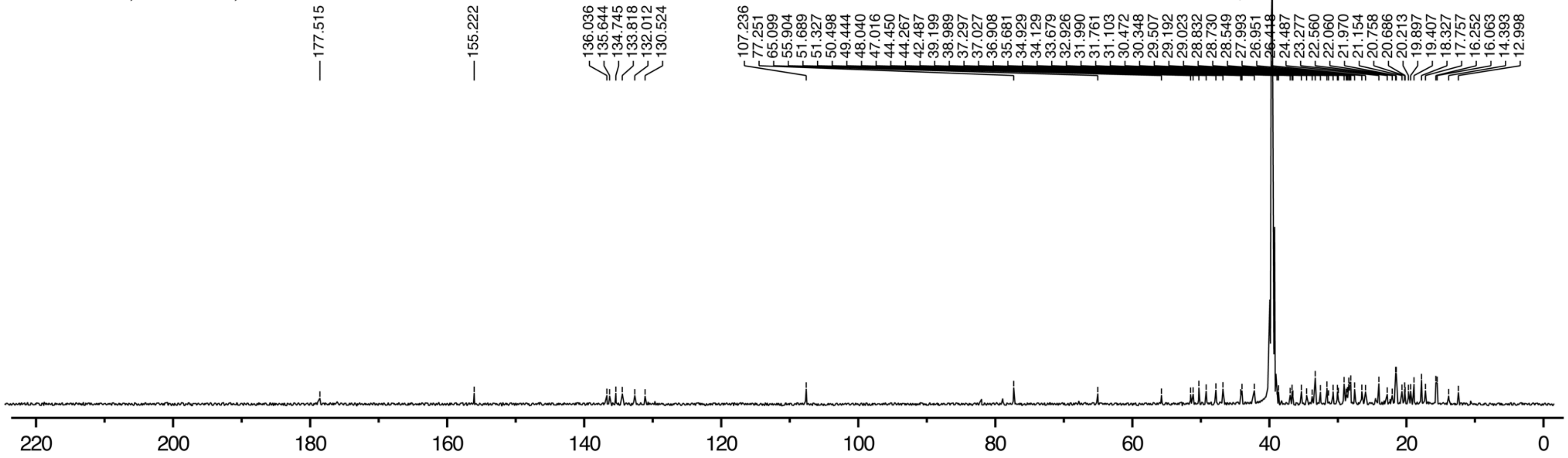

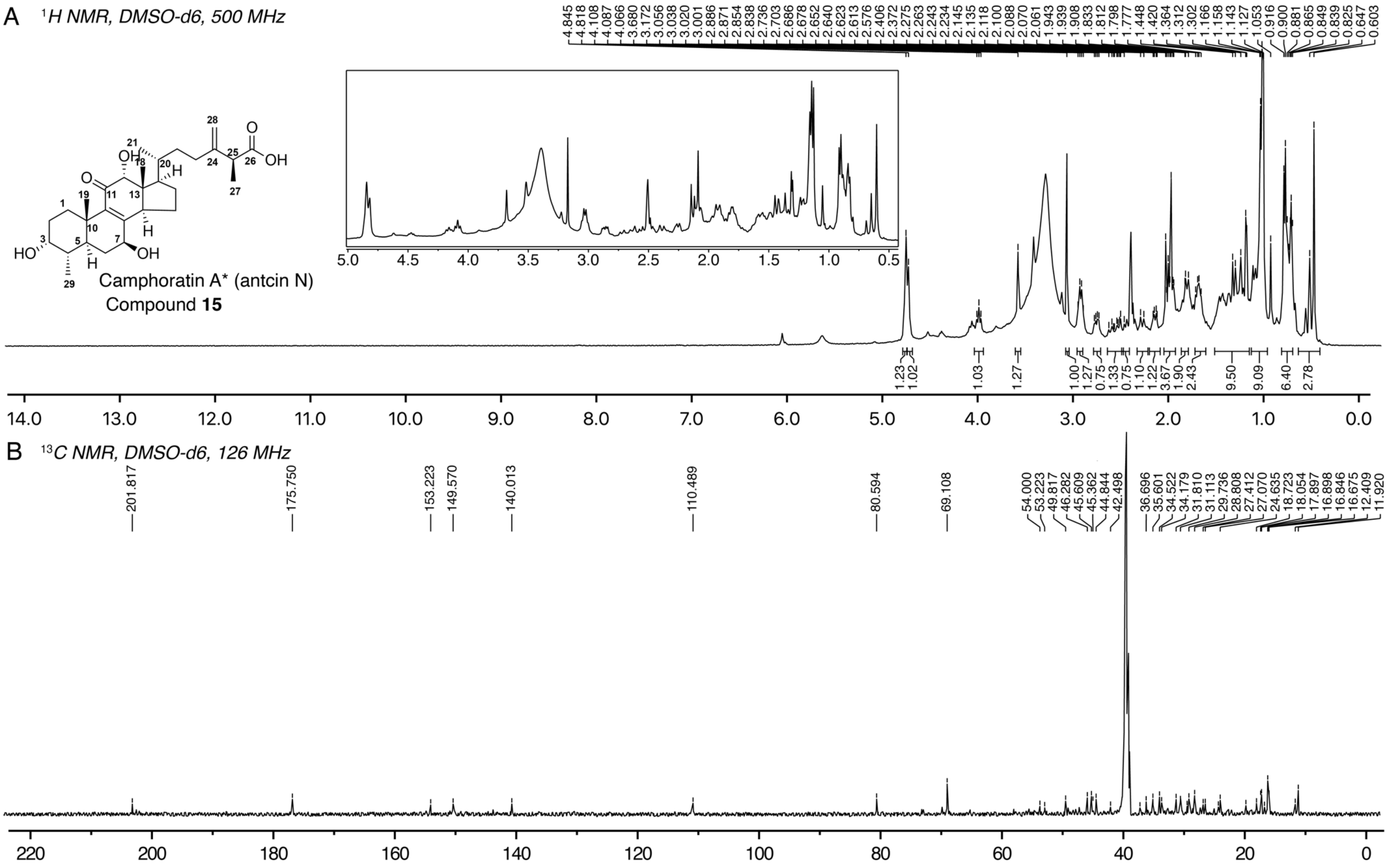
A
¹H NMR, DMSO-d6, 500 MHz
Camphoratin A* (antcin N)
Compound **15**
4.845 4.818 4.108 4.087 4.066 3.680 3.172 3.056 3.038 3.020 3.001 2.886 2.871 2.854 2.838 2.736 2.703 2.686 2.678 2.652 2.640 2.623 2.613 2.576 2.406 2.372 2.275 2.263 2.243 2.234 2.145 2.135 2.118 2.100 2.088 2.070 2.061 1.943 1.939 1.908 1.833 1.812 1.798 1.777 1.448 1.420 1.364 1.312 1.302 1.166 1.158 1.143 1.127 1.053 0.916 0.900 0.881 0.865 0.849 0.839 0.825 0.647 0.603
1.23 1.02 1.03 1.27 1.00 1.27 0.75 1.33 0.75 1.10 1.22 3.67 1.90 2.43 9.50 9.09 6.40 2.78
B
¹³C NMR, DMSO-d6, 126 MHz
201.817 175.750 153.223 149.570 140.013 110.489 80.594 69.108 54.000 53.223 49.817 46.282 45.609 45.362 44.844 42.498 36.696 35.601 34.522 34.179 31.810 31.113 29.736 28.808 27.412 27.070 24.635 18.723 18.054 17.897 16.846 16.675 12.409 11.920

A ^{1}H NMR, DMSO-d6, 500 MHz

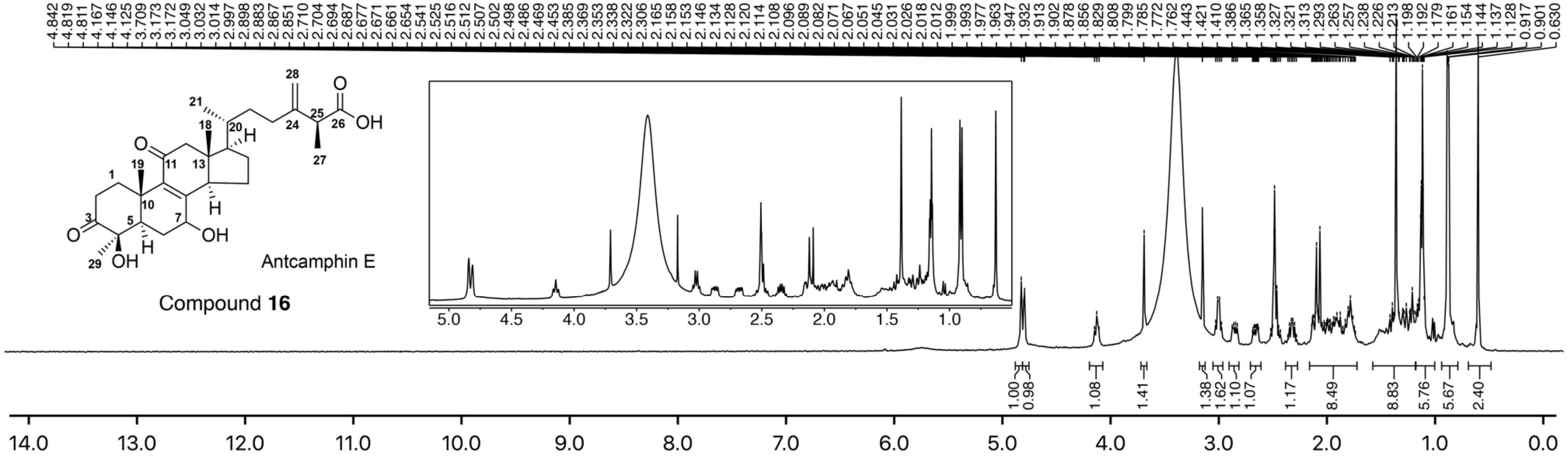

B ^{13}C NMR, DMSO-d6, 126 MHz

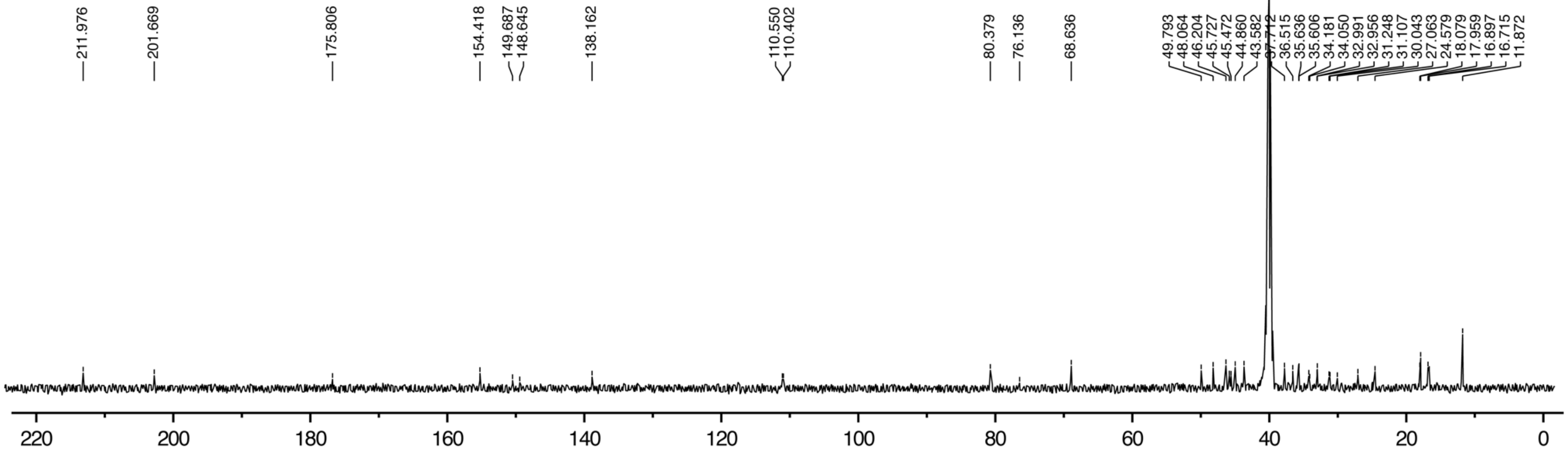

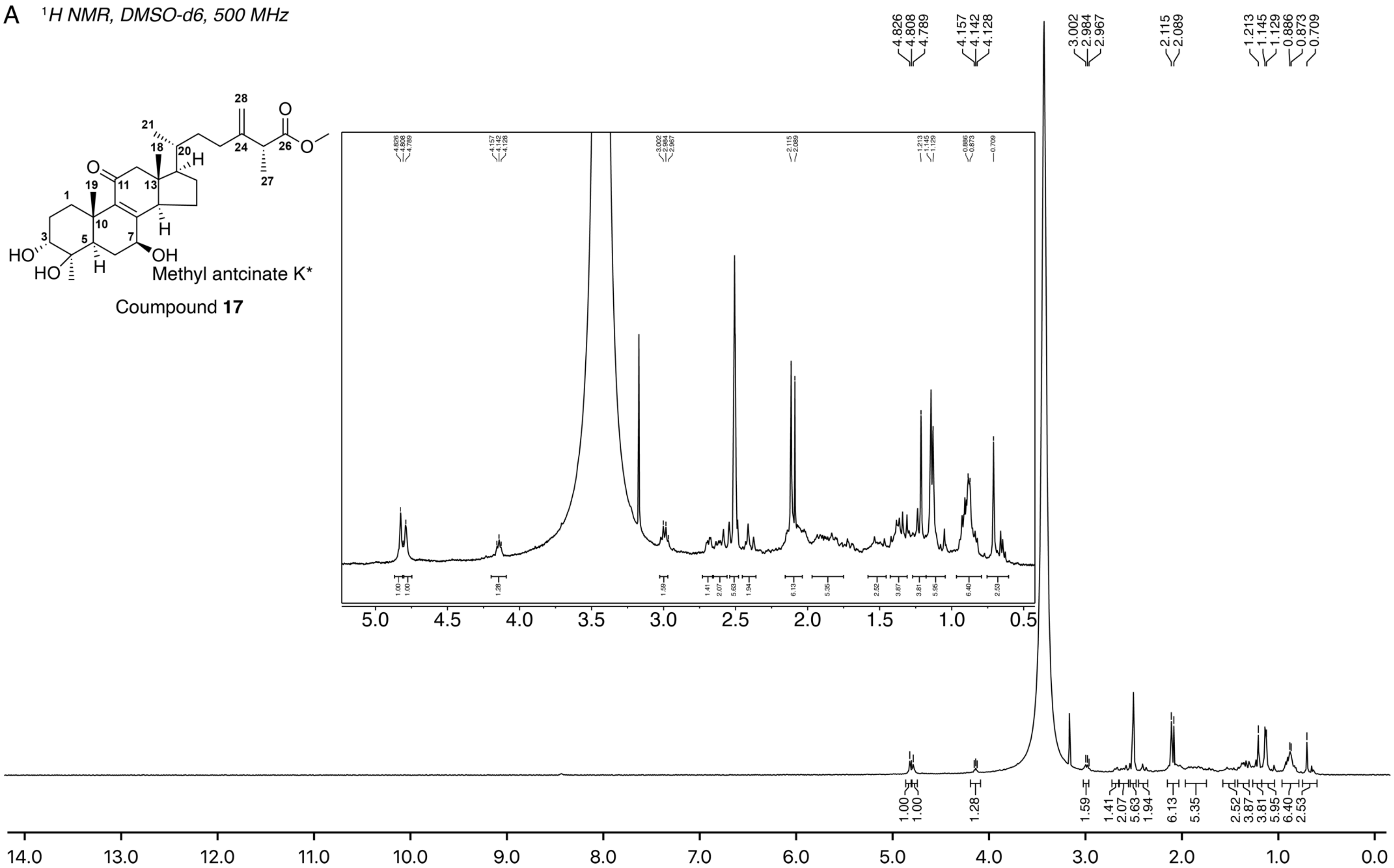
A
¹H NMR, DMSO-d6, 500 MHz
Methyl antcinate K*
Coumpound 17
4.826
4.808
4.789
4.157
4.142
4.128
3.002
2.984
2.967
2.115
2.089
1.213
1.145
1.129
0.886
0.873
0.709
1.00
1.00
1.28
1.59
1.41
2.07
5.63
1.94
6.13
5.35
2.52
3.87
3.81
5.95
6.40
2.53
14.0
13.0
12.0
11.0
10.0
9.0
8.0
7.0
6.0
5.0
4.0
3.0
2.0
1.0
0.0

A [1]*H NMR, DMSO-d6, 500 MHz*

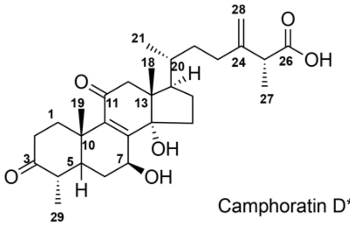

Camphoratin D*

Coumpound **18**

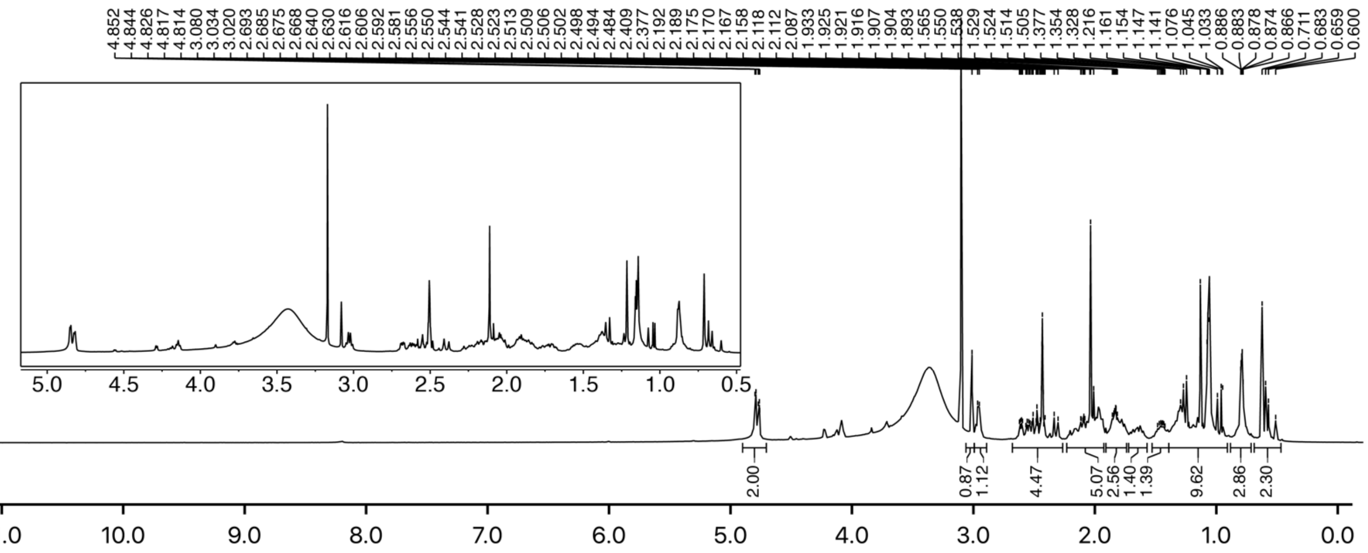

B [13]*C NMR, DMSO-d6, 126 MHz*

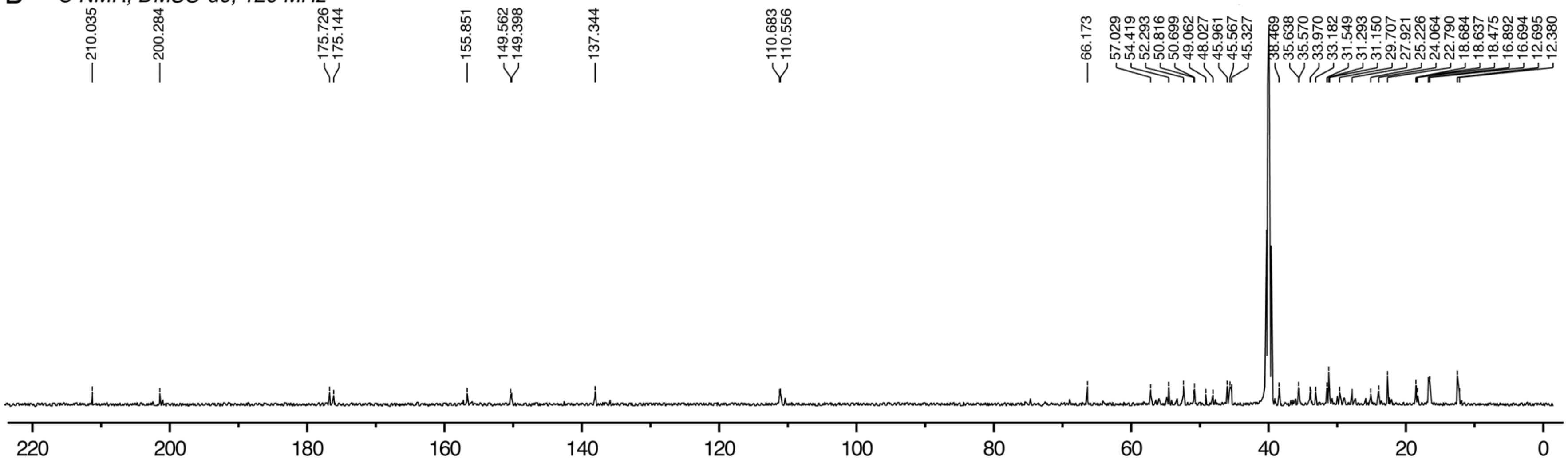

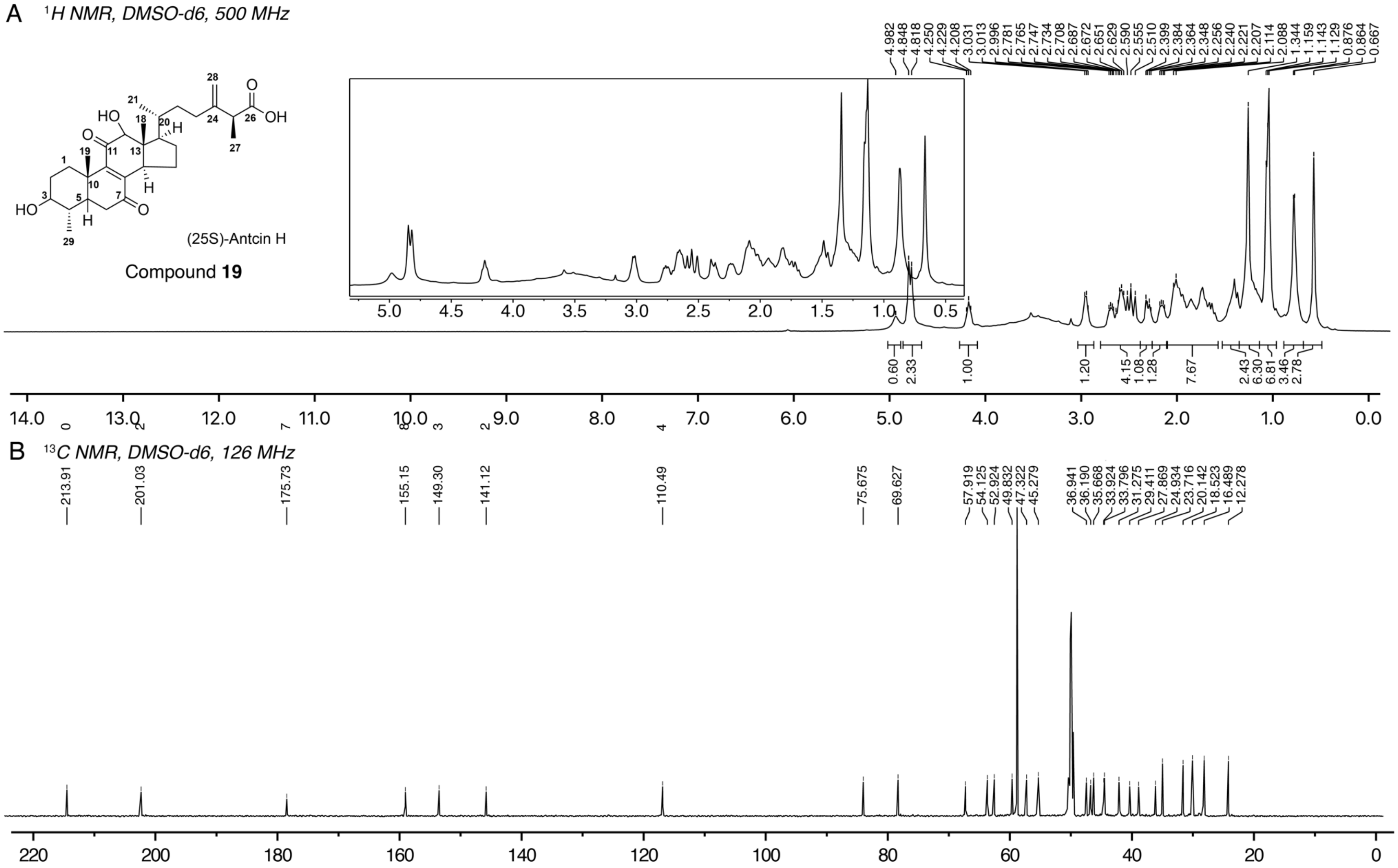

A
¹H NMR, DMSO-d6, 500 MHz
(25S)-Antcin H
Compound 19
4.982 4.848 4.818 4.250 4.229 4.208 3.031 3.013 2.996 2.781 2.765 2.747 2.734 2.708 2.687 2.672 2.651 2.629 2.590 2.555 2.510 2.399 2.384 2.364 2.348 2.256 2.240 2.221 2.207 2.114 2.088 1.344 1.159 1.143 1.129 0.876 0.864 0.667
0.60 2.33 1.00 1.20 4.15 1.08 1.28 7.67 2.43 6.30 6.81 3.46 2.78
B
¹³C NMR, DMSO-d6, 126 MHz
213.91 201.03 175.73 155.15 149.30 141.12 110.49 75.675 69.627 57.919 54.125 52.924 49.832 47.322 45.279 36.941 36.190 35.668 33.924 33.796 31.275 29.411 27.869 24.934 23.716 20.142 18.523 16.489 12.278

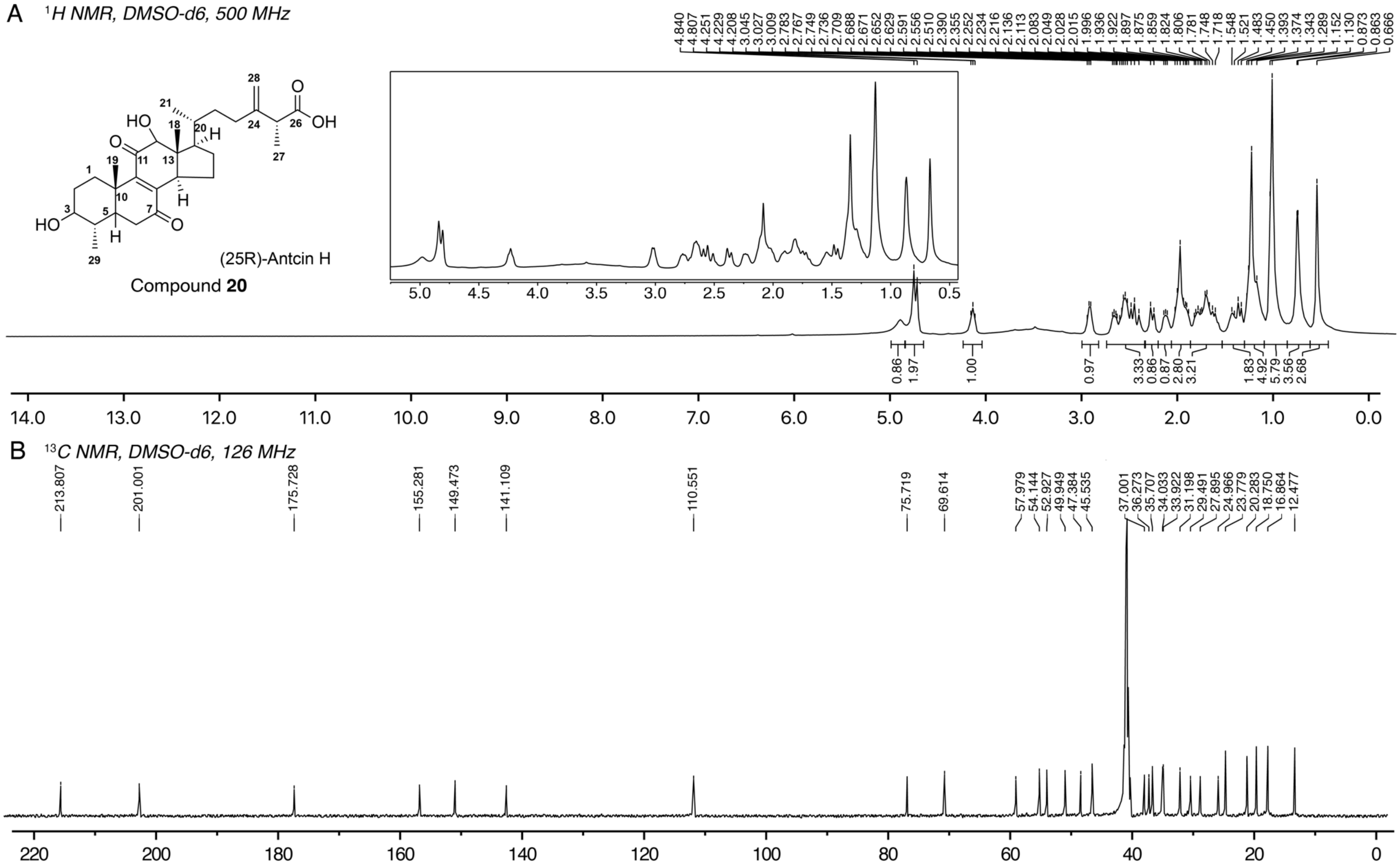

A
¹H NMR, DMSO-d6, 500 MHz
(25R)-Antcin H
Compound 20
4.840 4.807 4.251 4.229 4.208 3.045 3.027 3.009 2.783 2.767 2.749 2.736 2.709 2.688 2.671 2.652 2.629 2.591 2.556 2.510 2.390 2.355 2.252 2.234 2.216 2.136 2.113 2.083 2.049 2.028 2.015 1.996 1.936 1.922 1.897 1.875 1.859 1.824 1.806 1.781 1.748 1.718 1.548 1.521 1.483 1.450 1.393 1.374 1.343 1.289 1.152 1.130 0.873 0.863 0.666
0.86 1.97 1.00 0.97 3.33 0.86 0.87 2.80 3.21 1.83 4.92 5.79 3.56 2.68
B
¹³C NMR, DMSO-d6, 126 MHz
213.807 201.001 175.728 155.281 149.473 141.109 110.551 75.719 69.614 57.979 54.144 52.927 49.949 47.384 45.535 37.001 36.273 35.707 34.033 33.922 31.198 29.491 27.895 24.966 23.779 20.283 18.750 16.864 12.477

A

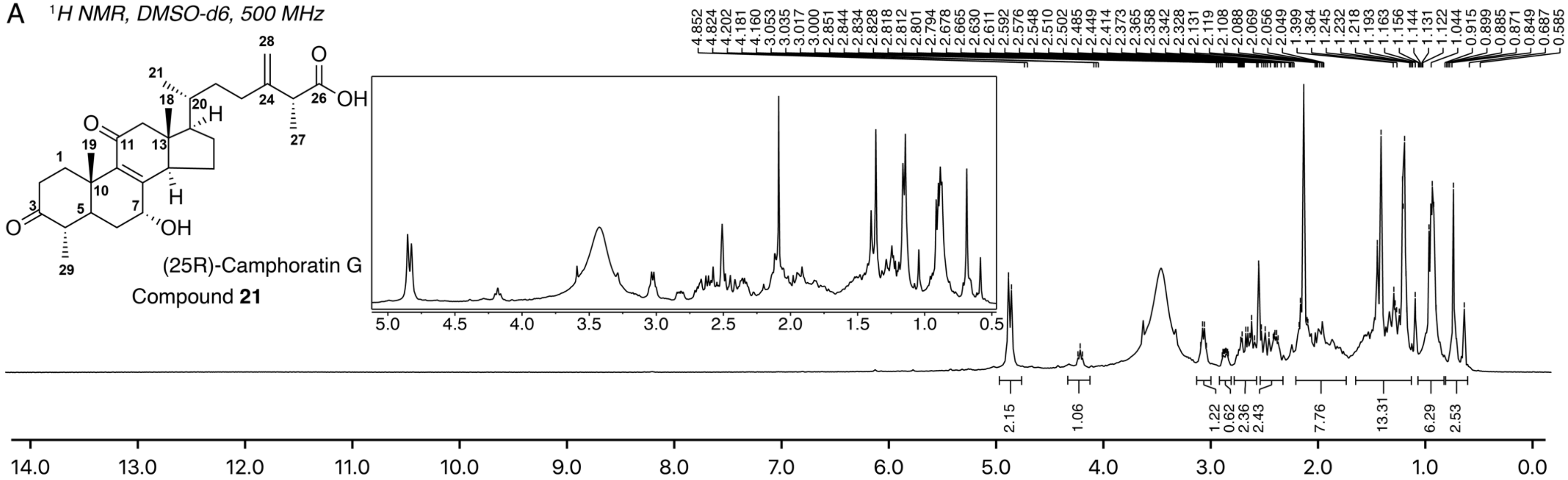
1H NMR, DMSO-d6, 500 MHz
28
21
18
20
24
26
OH
O
H
O
19
11
13
1
10
H
3
5
7
OH
O
29
27
(25R)-Camphoratin G
Compound 21
4.852 4.824 4.202 4.181 4.160 3.053 3.035 3.017 3.000 2.851 2.844 2.834 2.828 2.818 2.812 2.801 2.794 2.678 2.665 2.630 2.611 2.592 2.576 2.548 2.510 2.502 2.485 2.449 2.414 2.373 2.365 2.358 2.342 2.328 2.131 2.119 2.108 2.088 2.069 2.056 2.049 1.399 1.364 1.245 1.232 1.218 1.193 1.163 1.156 1.144 1.131 1.122 1.044 0.915 0.899 0.885 0.871 0.849 0.687 0.585
2.15 1.06 1.22 0.62 2.36 2.43 7.76 13.31 6.29 2.53
5.0 4.5 4.0 3.5 3.0 2.5 2.0 1.5 1.0 0.5
14.0 13.0 12.0 11.0 10.0 9.0 8.0 7.0 6.0 5.0 4.0 3.0 2.0 1.0 0.0

B

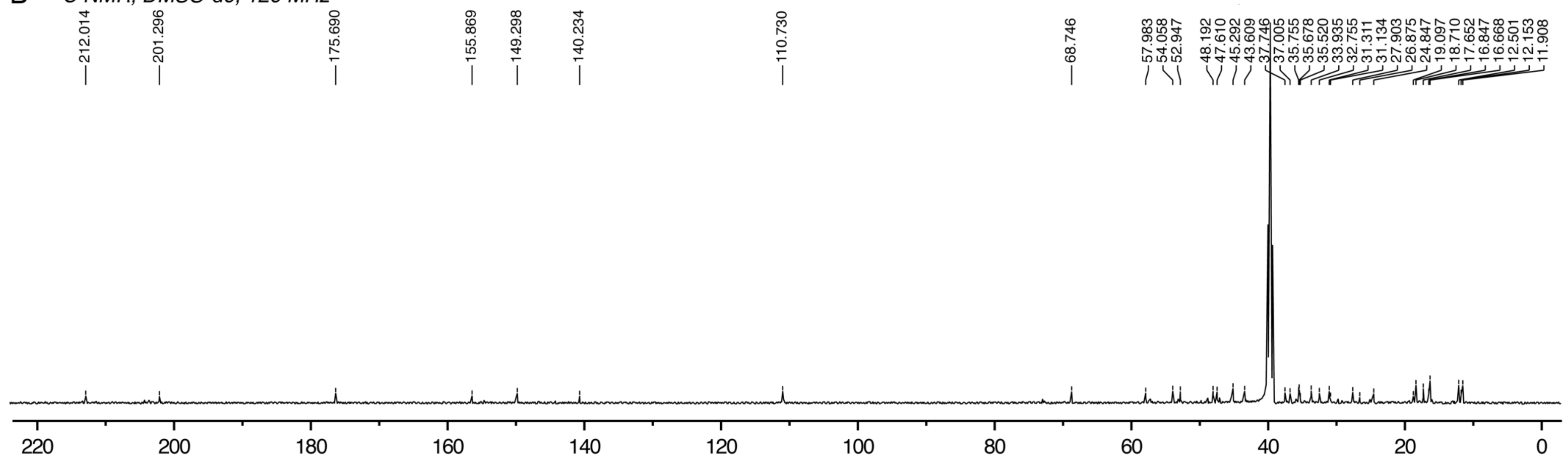
13C NMR, DMSO-d6, 126 MHz
212.014 201.296 175.690 155.869 149.298 140.234 110.730 68.746 57.983 54.058 52.947 48.192 47.610 45.292 43.609 37.746 37.005 35.755 35.678 35.520 33.935 32.755 31.311 31.134 27.903 26.875 24.847 19.097 18.710 17.652 16.847 16.668 12.501 12.153 11.908
220 200 180 160 140 120 100 80 60 40 20 0

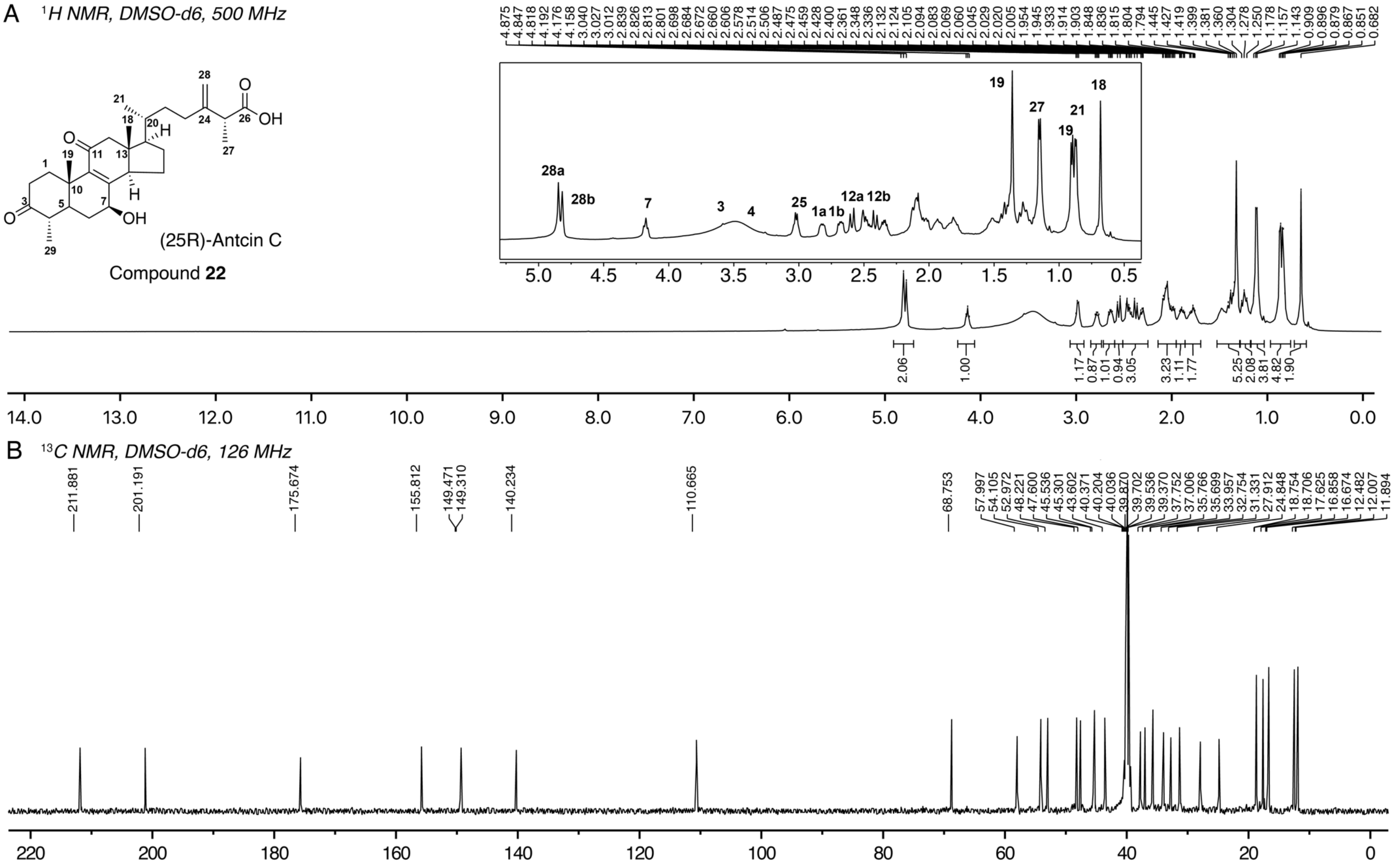

A
1H NMR, DMSO-d6, 500 MHz
4.875 4.847 4.818 4.192 4.176 4.158 3.040 3.027 3.012 2.839 2.826 2.813 2.801 2.698 2.684 2.672 2.660 2.606 2.578 2.514 2.506 2.487 2.475 2.459 2.428 2.400 2.361 2.348 2.336 2.132 2.124 2.105 2.094 2.083 2.069 2.060 2.045 2.029 2.020 2.005 1.954 1.945 1.933 1.914 1.903 1.848 1.836 1.815 1.804 1.794 1.445 1.427 1.419 1.399 1.381 1.360 1.304 1.278 1.250 1.178 1.157 1.143 0.909 0.896 0.879 0.867 0.851 0.682
(25R)-Antcin C
Compound 22
28a 28b 7 3 4 25 1a 1b 12a 12b 19 27 21 19 18
2.06 1.00 1.17 0.87 1.01 0.94 3.05 3.23 1.11 1.77 5.25 2.08 3.81 4.82 1.90
B
13C NMR, DMSO-d6, 126 MHz
211.881 201.191 175.674 155.812 149.471 149.310 140.234 110.665 68.753 57.997 54.105 52.972 48.221 47.600 45.536 45.301 43.602 40.371 40.204 40.036 39.870 39.702 39.536 39.370 37.752 37.006 35.766 35.699 33.957 32.754 31.331 27.912 24.848 18.754 18.706 17.625 16.858 16.674 12.482 12.007 11.894

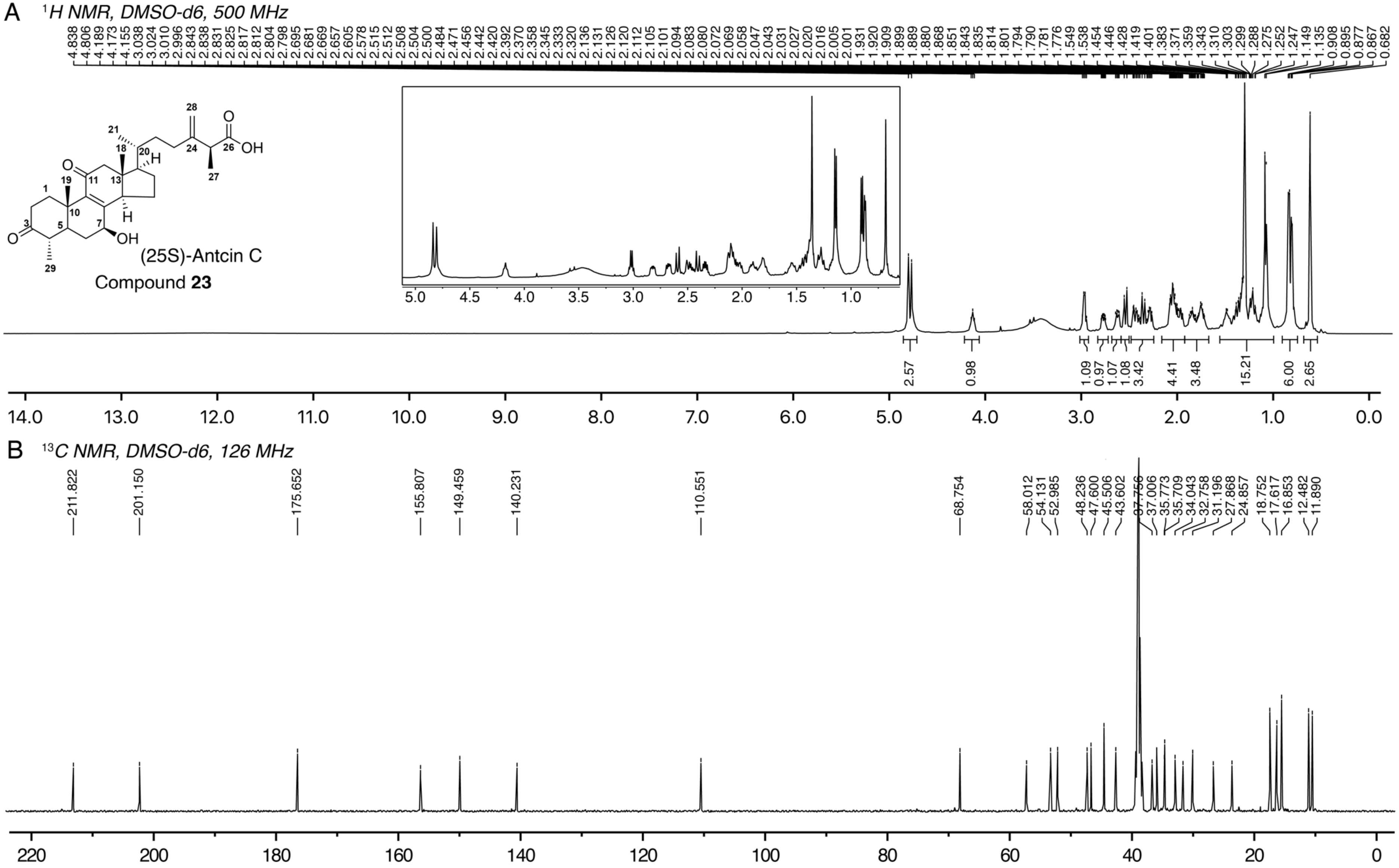
A
1H NMR, DMSO-d6, 500 MHz
4.838 4.806 4.189 4.173 4.155 3.038 3.024 3.010 2.996 2.843 2.838 2.831 2.825 2.817 2.812 2.804 2.798 2.695 2.681 2.669 2.657 2.605 2.578 2.515 2.512 2.508 2.504 2.500 2.484 2.471 2.456 2.442 2.420 2.392 2.370 2.358 2.345 2.333 2.320 2.136 2.131 2.126 2.120 2.112 2.105 2.101 2.094 2.083 2.080 2.072 2.069 2.058 2.047 2.043 2.031 2.027 2.020 2.016 2.005 2.001 1.931 1.920 1.909 1.899 1.889 1.880 1.868 1.851 1.843 1.835 1.814 1.801 1.794 1.790 1.781 1.776 1.549 1.538 1.454 1.446 1.428 1.419 1.401 1.383 1.371 1.359 1.343 1.310 1.303 1.299 1.288 1.275 1.252 1.247 1.149 1.135 0.908 0.895 0.877 0.867 0.682
28
21
18
20
H
24
26
O
OH
27
O
11
13
19
1
10
H
3
5
7
O
OH
29
(25S)-Antcin C
Compound 23
5.0 4.5 4.0 3.5 3.0 2.5 2.0 1.5 1.0
2.57 0.98 1.09 0.97 1.07 1.08 3.42 4.41 3.48 15.21 6.00 2.65
14.0 13.0 12.0 11.0 10.0 9.0 8.0 7.0 6.0 5.0 4.0 3.0 2.0 1.0 0.0
B
13C NMR, DMSO-d6, 126 MHz
211.822 201.150 175.652 155.807 149.459 140.231 110.551 68.754 58.012 54.131 52.985 48.236 47.600 45.506 43.602 37.756 37.006 35.773 35.709 34.043 32.758 31.196 27.868 24.857 18.752 17.617 16.853 12.482 11.890
220 200 180 160 140 120 100 80 60 40 20 0

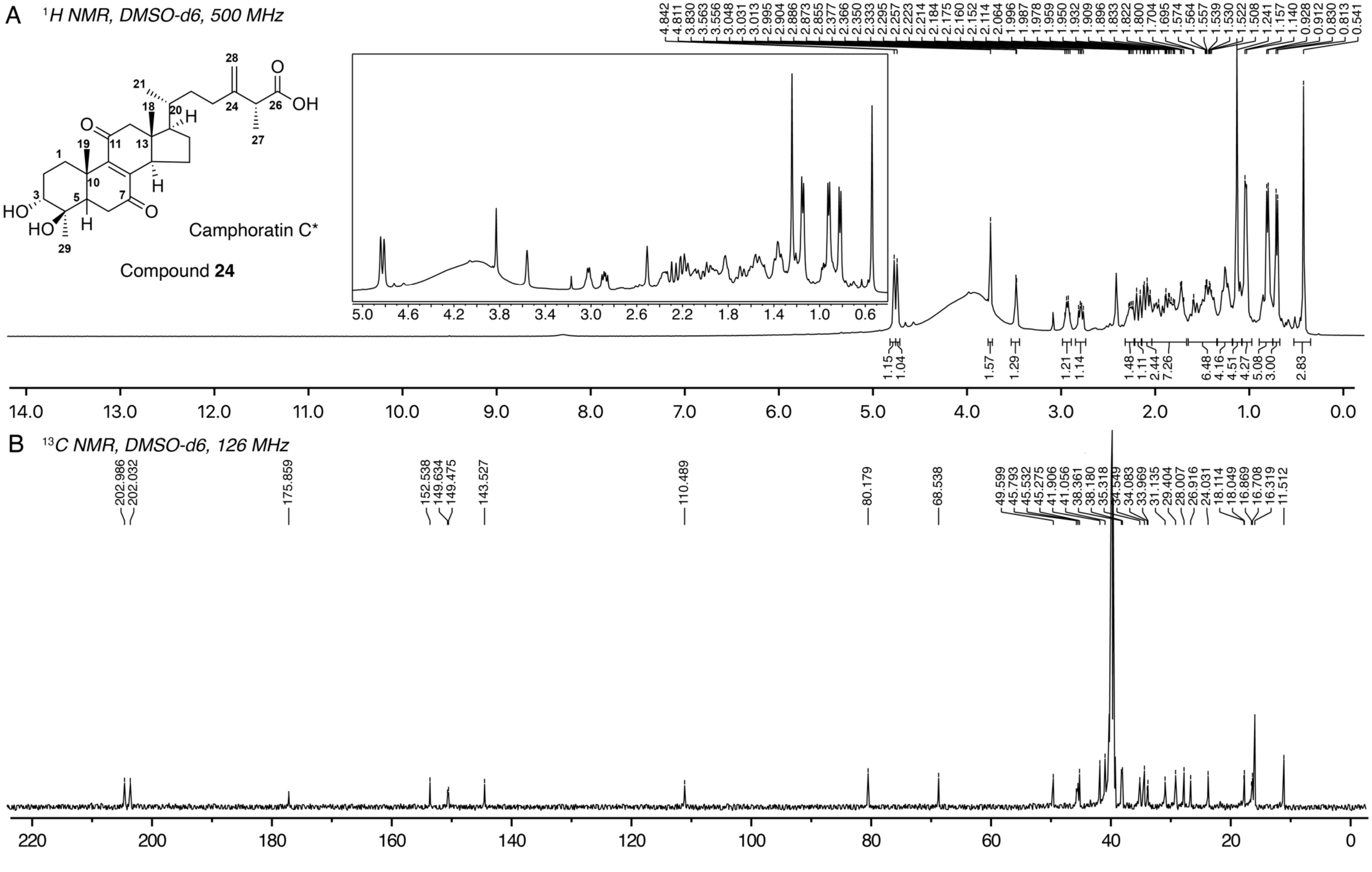
A
1H NMR, DMSO-d6, 500 MHz
Camphoratin C*
Compound 24
4.842 4.811 3.830 3.563 3.556 3.048 3.031 3.013 2.995 2.904 2.886 2.873 2.855 2.377 2.366 2.350 2.333 2.295 2.257 2.223 2.214 2.184 2.175 2.160 2.152 2.114 2.064 1.996 1.987 1.978 1.959 1.950 1.932 1.909 1.896 1.833 1.822 1.800 1.704 1.695 1.574 1.564 1.557 1.539 1.530 1.522 1.508 1.241 1.157 1.140 0.928 0.912 0.830 0.813 0.541
1.15 1.04 1.57 1.29 1.21 1.14 1.48 1.11 2.44 7.26 6.48 4.16 4.51 4.27 5.08 3.00 2.83
B
13C NMR, DMSO-d6, 126 MHz
202.986 202.032 175.859 152.538 149.634 149.475 143.527 110.489 80.179 68.538 49.599 45.793 45.532 45.275 41.906 41.056 38.361 38.180 35.318 34.549 34.083 33.969 31.135 29.404 28.007 26.916 24.031 18.114 18.049 16.869 16.708 16.319 11.512

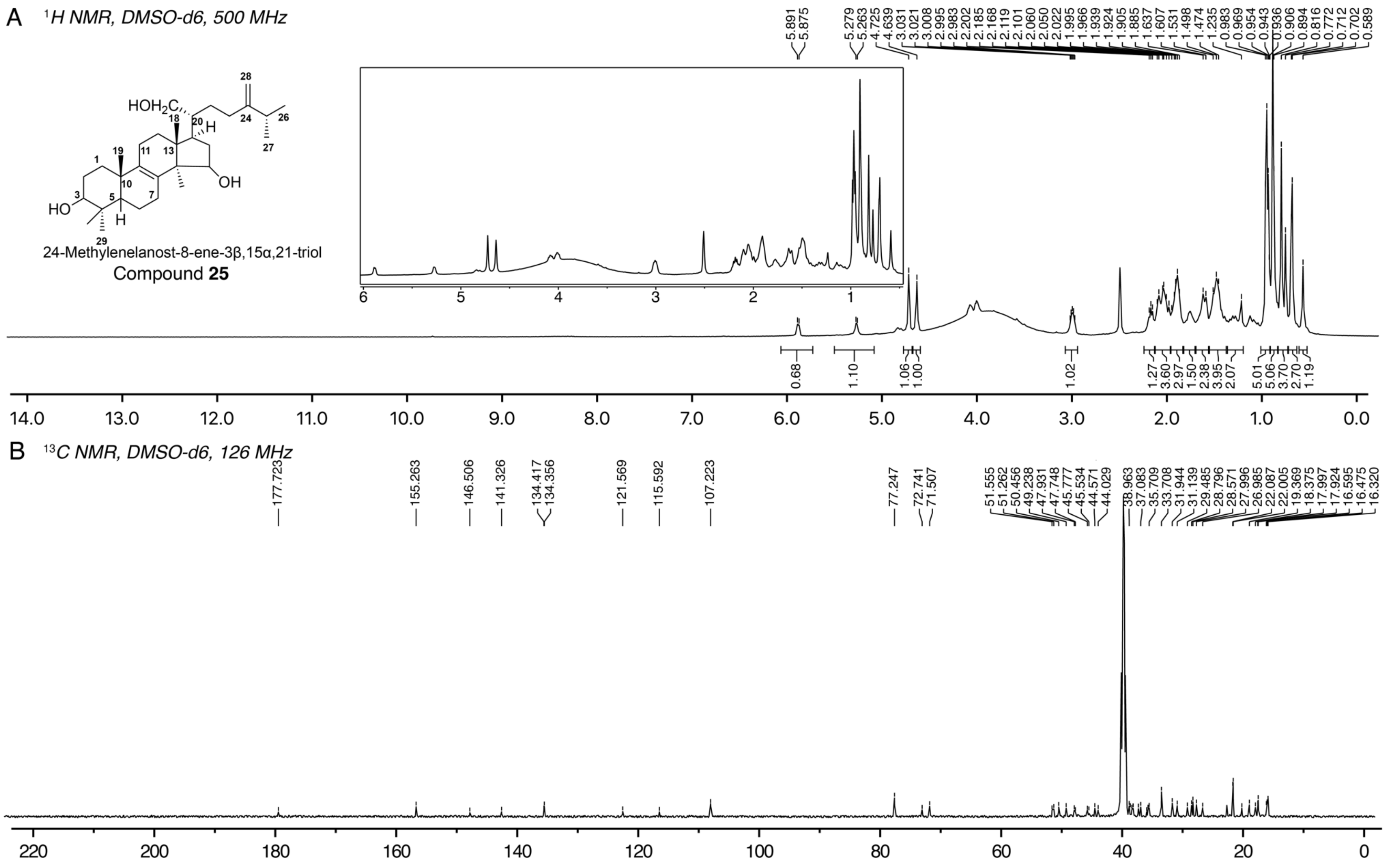
A
1H NMR, DMSO-d6, 500 MHz
5.891
5.875
5.279
5.263
4.725
4.639
3.031
3.021
3.008
2.995
2.983
2.202
2.185
2.168
2.119
2.101
2.060
2.050
2.022
1.995
1.966
1.939
1.924
1.905
1.885
1.637
1.607
1.531
1.498
1.474
1.235
0.983
0.969
0.954
0.943
0.936
0.906
0.894
0.816
0.772
0.712
0.702
0.589
HOH2C
28
24
26
27
18
20
H
19
11
13
1
10
3
5
7
HO
H
OH
29
24-Methylenelanost-8-ene-3β,15α,21-triol
Compound 25
0.68
1.10
1.06
1.00
1.02
1.27
3.60
2.97
1.50
2.38
3.95
2.07
5.01
5.06
3.70
2.70
1.19
14.0
13.0
12.0
11.0
10.0
9.0
8.0
7.0
6.0
5.0
4.0
3.0
2.0
1.0
0.0
B
13C NMR, DMSO-d6, 126 MHz
177.723
155.263
146.506
141.326
134.417
134.356
121.569
115.592
107.223
77.247
72.741
71.507
51.555
51.262
50.456
49.238
47.931
47.748
45.777
45.534
44.571
44.029
38.963
37.083
35.709
33.708
31.944
31.139
29.485
28.796
28.571
27.996
26.985
22.087
22.005
19.369
18.375
17.997
17.924
16.595
16.475
16.320
220
200
180
160
140
120
100
80
60
40
20
0

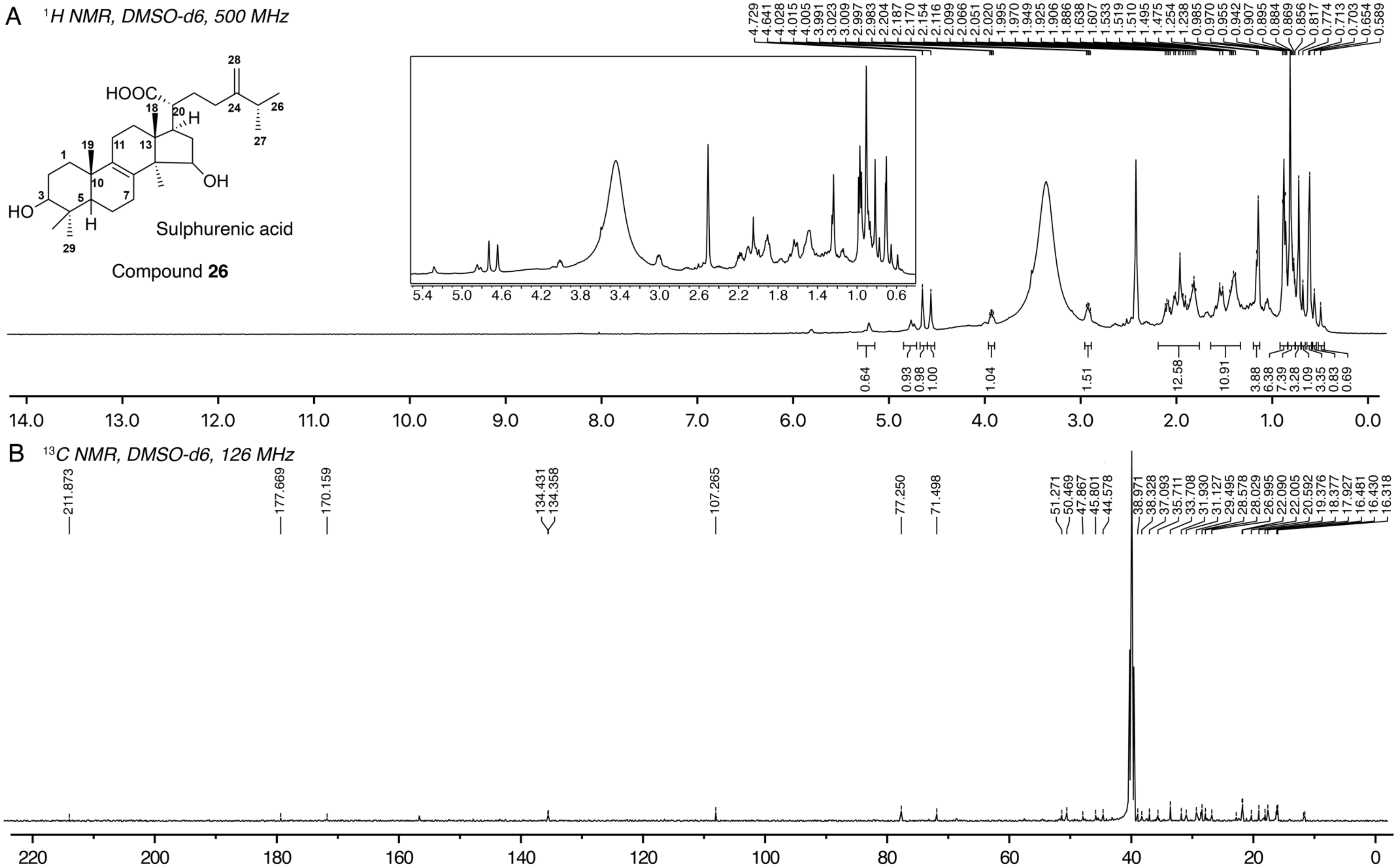
A
1H NMR, DMSO-d6, 500 MHz
Sulphurenic acid
Compound 26
4.729 4.641 4.028 4.015 4.005 3.991 3.023 3.009 2.997 2.983 2.204 2.187 2.170 2.154 2.116 2.099 2.066 2.051 2.020 1.995 1.970 1.949 1.925 1.906 1.886 1.638 1.607 1.533 1.519 1.510 1.495 1.475 1.254 1.238 0.985 0.970 0.955 0.942 0.907 0.895 0.884 0.869 0.856 0.817 0.774 0.713 0.703 0.654 0.589
0.64 0.93 0.98 1.00 1.04 1.51 12.58 10.91 3.88 6.38 7.39 3.28 1.09 3.35 0.83 0.69
B
13C NMR, DMSO-d6, 126 MHz
211.873 177.669 170.159 134.431 134.358 107.265 77.250 71.498 51.271 50.469 47.867 45.801 44.578 38.971 38.328 37.093 35.711 33.708 31.930 31.127 29.495 28.578 28.029 26.995 22.090 22.005 20.592 19.376 18.377 17.927 16.481 16.430 16.318

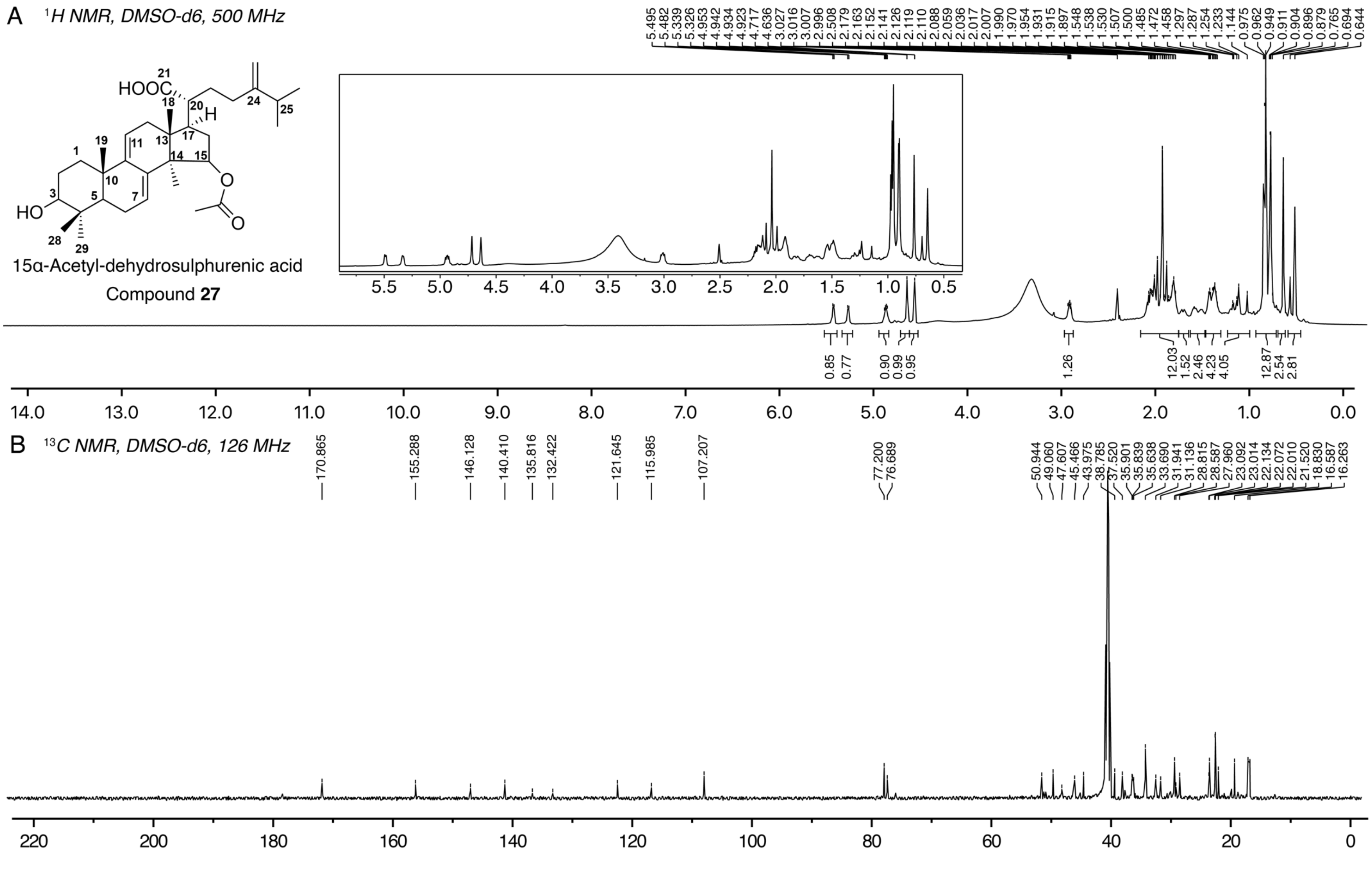
A
^{1}H NMR, DMSO-d6, 500 MHz
HOOC
21
18
20
H
24
25
19
13
17
1
11
14
15
10
3
5
7
HO
O
O
28
29
15α-Acetyl-dehydrosulphurenic acid
Compound **27**
5.495 5.482 5.339 5.326 4.953 4.942 4.934 4.923 4.717 4.636 3.027 3.016 3.007 2.996 2.508 2.179 2.163 2.152 2.141 2.126 2.119 2.110 2.088 2.059 2.036 2.017 2.007 1.990 1.970 1.954 1.931 1.915 1.897 1.548 1.538 1.530 1.507 1.500 1.485 1.472 1.458 1.297 1.287 1.254 1.233 1.144 0.975 0.962 0.949 0.911 0.904 0.896 0.879 0.765 0.694 0.644
5.5 5.0 4.5 4.0 3.5 3.0 2.5 2.0 1.5 1.0 0.5
0.85 0.77 0.90 0.99 0.95 1.26 12.03 1.52 2.46 4.23 4.05 12.87 2.54 2.81
14.0 13.0 12.0 11.0 10.0 9.0 8.0 7.0 6.0 5.0 4.0 3.0 2.0 1.0 0.0
B
^{13}C NMR, DMSO-d6, 126 MHz
170.865 155.288 146.128 140.410 135.816 132.422 121.645 115.985 107.207 77.200 76.689 50.944 49.060 47.607 45.466 43.975 38.785 37.520 35.901 35.839 35.638 33.690 31.941 31.136 28.815 28.587 27.960 23.092 23.014 22.134 22.072 22.010 21.520 18.830 16.587 16.263
220 200 180 160 140 120 100 80 60 40 20 0

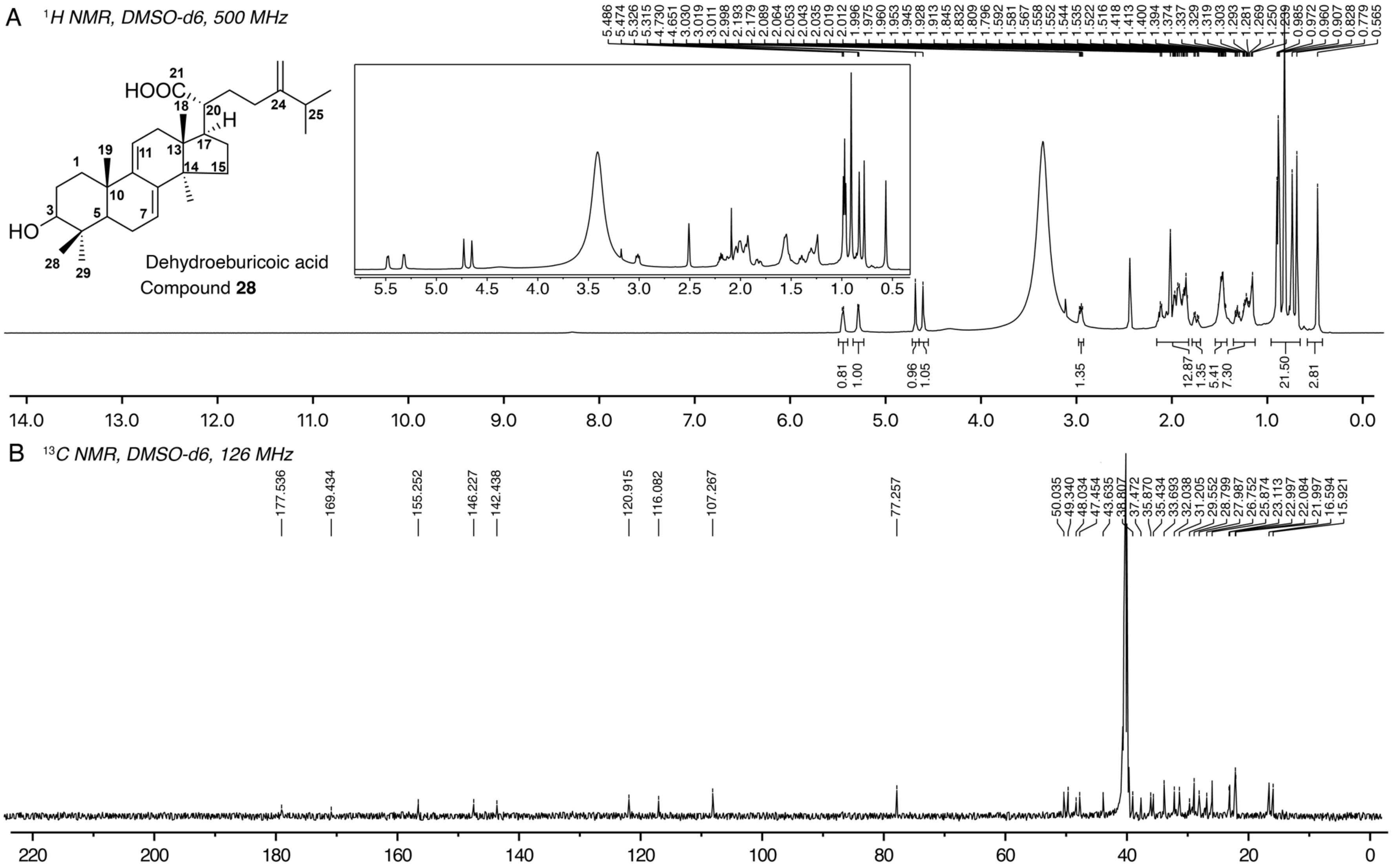

A
¹H NMR, DMSO-d6, 500 MHz
Dehydroeburicoic acid
Compound 28
HOOC
HO
B
¹³C NMR, DMSO-d6, 126 MHz
177.536
169.434
155.252
146.227
142.438
120.915
116.082
107.267
77.257
